# Effectiveness of bullying prevention-associated interventions among children and adolescents: an umbrella review of systematic review and meta-analysis

**DOI:** 10.1186/s12916-026-04974-x

**Published:** 2026-06-17

**Authors:** Zi-Wei Li, Li Lu, Xiao-Dong Qin, Rong Li, Zhongliang Zhou, Shou Liu, Qi Liu, Sha Lai, Qing Shen

**Affiliations:** 1 Institute of Health Management and Policy, School of Public Policy and Administration, Xi’an Jiaotong University, Xi’an, China; 2 System Behavior and Management Laboratory, Philosophy and Social Sciences Laboratory of the Ministry of Education, Xi’an Jiaotong University, Xi’an, China; 3 Department of Public Health, Medical College, Qinghai University, Xining, China; 4 School of Management, Xi’an Jiaotong University, Xi’an, China; 5 Clinical Research Center for Mental Disorders, Shanghai Pudong New Area Mental Health Center, Tongji University School of Medicine, Shanghai, China; 6 Institute for Advanced Study, Tongji University, Shanghai, China; 7 Unit of Integrative Epidemiology, Institute of Environmental Medicine, Karolinska Institutet, Stockholm, Sweden

**Keywords:** Bullying, Prevention, Intervention, Umbrella review, Meta-analysis, Effectiveness

## Abstract

**Background:**

Bullying represents a critical global health concern among children and adolescents, emphasizing an urgent need for effective prevention interventions. Current meta-analyses frequently focus on single prevention interventions or outcomes, limiting the generalizability of findings for cross-intervention comparisons. We aimed to perform an umbrella review to provide an overview of the effectiveness of bullying prevention interventions.

**Methods:**

Potential literature was searched from PubMed, Web of Science, Embase, and EBSCO from their respective inception to October 22, 2024. Three reviewers independently selected meta-analysis of bullying prevention intervention that reported any type of prevention intervention and assessed outcomes in children and adolescents. Following PRISMA guidelines, three reviewers independently extracted data and evaluated study quality using AMSTAR-2. All effect estimates were converted to Cohen’s d for cross-domain comparisons.

**Results:**

4740 studies were identified with 30 meta-analyses included. Studies reporting statistical significance all demonstrated beneficial effects of bullying prevention interventions. Included meta-analyses evaluated 17 distinct prevention interventions in alleviating bullying via assessment on three main outcomes with 49 distinct sub-outcomes, i.e., behavior change (36 sub-outcomes, the most frequent target), mental health (9 sub-outcomes), and attitude change (4 sub-outcomes). 30 studies were categorized into five categories based on the leading role of prevention interventions: family-based (*n* = 2), school-based (*n* = 10), community-based (*n* = 2), healthcare-based (*n* = 5), and collaborative (*n* = 11). Cohen’s d values ranged from −0.99 to 1.14, with the music-based intervention exhibiting the largest observed point estimate (Cohen’s d = −0.99, 95% confidence interval [CI]: −1.42 to −0.56), which was also the largest among interventions targeting behavior change. Martial arts presented largest observed point estimate among collaborative prevention interventions, while digital health intervention, anti-aggression program, and parent-related intervention showed the largest observed point estimates among community-based, school-based, and family-based prevention interventions, respectively. For mental health, sports intervention exhibited the largest observed point estimate. And for attitude change, anti-bullying intervention presented the largest estimate, consistent with results from RCT-only subgroup.

**Conclusions:**

Bullying prevention interventions may be associated with improvements in positive behavior change, attitude change, and mental health, with substantial variations in effectiveness. These findings regarding comparative efficacy may inform the strategic selection of preventive interventions in future evaluation and implementation efforts.

**PROSPERO registration number:**

CRD42024601712

## Background

Bullying, a common form of violence towards children and adolescents [1], was defined as unwanted aggressive behaviors by another youth or group of youths who were not siblings or current dating partners [2]. Bullying usually occurs repeatedly and involves a perceived or observed power imbalance favoring the perpetrators [2–4]. Bullying is becoming a multifaceted challenge to public safety globally and a threat to mental and physical well-being of the victims [5, 6]. The World Health Organization (WHO) reported an estimated prevalence of 6 and 11% of adolescents ever engaged in bullying perpetration and victimization [7]. The prevalence of bullying victimization among existing studies varied from 11.1 to 42.2% [8–10], and the prevalence of bullying perpetration varied from 10.4 to 24.4% [8, 11, 12]. Exposure to bullying exerts a long-term adverse effect on multiple detrimental outcomes among children and adolescents [13], such as externalizing behavior, aggressive behavior, anger, hostility, and mental health problems, etc. [14–16]. The alarmingly high prevalence of bullying, and its likely increasing trend during and after the pandemic [17, 18], along with the persistent detrimental consequences, underscore an urgent need for interventions to prevent bullying, especially among vulnerable groups exposed during childhood and adolescence.

Ecosystem theory posits that individual development is shaped by a set of interacting nested systems including microsystem, mesosystem, exosystem, and macrosystem, which encompass contexts ranging from proximal environments (e.g., family, school) to distal societal structures (e.g., social institutions and healthcare systems), with developmental outcomes further modulated by the interactive collaboration across these hierarchical system levels [19]. Bullying, notably, manifests precisely within this complex ecosystem, and the existing bullying prevention interventions mainly take actions from five leading roles, i.e., family (e.g., parenting programs) [20], school (e.g., anti-bullying interventions) [15], community (e.g., community-based programs) [21], healthcare (e.g., cognitive behavioral interventions) [22], and collaborative (e.g., educational interventions) [23]. Social cognitive theory (SCT) postulates reciprocal interactions among personal factors (e.g., cognitive abilities, outcomes expectations, and physical and mental functioning), behavioral, and environmental factors [24, 25]. Guided by this theoretical framework [24, 25], and considering the complexity and multidimensionality inherent in evaluating the effectiveness of bullying prevention interventions, the present study defined three outcome dimensions: behavior change (e.g., bullying victimization/perpetration, bystander, etc.) [15], attitude change (e.g., school climate, etc.) [15], and mental health improvement (e.g., depression symptoms, etc.) [20]. These dimensions align with the core SCT constructs of behavioral responses, internal cognitive-affective states, and psychological functioning, which were posited to underpin the efficacy of prevention interventions. However, the generalizability of existing evidence was limited by the heterogeneity of methodological approaches employed, such as variations in effect estimates, study settings and statistical methodologies [15, 26], which complicated the interpretation and cross-study comparison of these findings. Moreover, most existing meta-analyses mainly focused on one specific type of prevention intervention or on limited outcomes [15, 27, 28], leaving a lack of a comprehensive summary of findings from these meta-analyses.

Although existing studies have indicated the effectiveness of bullying prevention interventions among children and adolescents, a broad overview capturing “who leads” “what works” “how to classify” “which is superior” “where effect” is still pending. A previous umbrella review has examined targeted violence prevention interventions in general populations [29], however, to date, there is no umbrella review to provide a comprehensive overview of the effectiveness of bullying prevention interventions and the full range of related outcomes among children and adolescents. Therefore, we conducted this exposure-wide umbrella review to 1) systematically examine the effectiveness of bullying prevention interventions on children and adolescents; 2) compare the effectiveness of bullying prevention interventions by different sub-prevention interventions and sub-outcomes.

## Methods

We conducted the literature search following the Preferred Reporting Items for Systematic Reviews and Meta-Analyses (PRISMA) guidelines [30, 31] (Additional file 1: Table S1) and Joanna Briggs Institute (JBI) methodological guidance [32]. The research protocol was registered in the International Prospective Register of Systematic Reviews (PROSPERO; registration number: CRD42024601712). It should be emphasized that a systematic review, ideally, should be conducted in strict adherence to its registered and published protocol. While protocol registration is pivotal for advancing the methodological rigor of systematic reviews, it should not be regarded as a rigid mandate that precludes iterative refinement [33]. In contrast, to further improve the transparency of the review process, all modifications to the original protocol must be explicitly documented and substantiated with clear, evidence-based justifications [33–35]. A summary of discrepancies between the present review and its published protocol is provided in Additional file 1: Table S2 [15, 36–39].

### Search strategies

Four English databases including PubMed, Web of Science (WoS), Embase, and EBSCO were searched from database inception to October 22, 2024, by three authors (ZWL, RL and XDQ) individually.

The same combination of the search terms was employed to search the title and abstract of potential articles in the four databases: (child* OR adolescent OR teen* OR youth) AND (bullying OR anti-bullying OR “school bullying” OR cyberbullying OR victimization OR “bullying victimization” OR cyber-victimization OR abuse OR violence OR aggression) AND (intervention OR prevention OR treatment OR therapy OR curriculum) AND (“systematic review” OR meta-analysis). Detailed search strategies for each database are presented in Additional file 1: Table S3. Further, we manually screened the reference lists of included meta-analyses or systematic reviews for additional inclusion of relevant studies.

### Study selection

Studies were included if they met the following inclusion criteria: (a) systematic review and meta-analysis published in English academic journals with full-text available; (b) involving bullying behaviors in children (<18 years) and adolescents, the latter was defined as individuals aged 10–24 years since it was noted that this age range aligns more closely with adolescent growth and popular understandings of this life phase, which could facilitate extended investments across diverse contexts [36]. Children were defined as individuals aged < 18 years [37]; (c) focusing on any type of bullying prevention intervention; (d) meta-analyses synthesizing data of studies with a comparison or control group without prevention interventions or with alternative prevention interventions; (e) assessing outcomes following the bullying prevention interventions, such as behavior change, attitude change, and mental health; and (f) providing Cohen’s d or other measures [e.g., mean difference (MD), relative risk (RR), odds ratios (OR), Hedges’ g, standardized mean difference (SMD)] that can be converted to Cohen’s d, since it was widely reported in existing reviews [15, 38].

Meta-analyses without providing an explicit list or specific information (such as first author, and publication information) of included original studies were excluded. Studies without meta-analyzed data (e.g., systematic reviews, meta-reviews) were excluded since we intended to synthesize quantitative comparisons and summarize quality of evidence. In addition, studies focusing on children and adolescents with specific clinically diagnosed disorders (e.g., autism spectrum disorder, attention-deficit/ hyperactivity disorder, etc.) were not included, as the present study aimed to center on the general pediatric and adolescent population. To prevent overlap, the meta-analysis with the largest number of original studies was included [29]. Examples of excluded meta-analyses are shown in Additional file 1: Table S4 [40], and an example of the details of one excluded and one included meta-analysis is shown in Additional file 1: Table S5 [40, 41]. When the meta-analysis presented pooled estimates for multiple outcomes, each value was used for each outcome and included separately in our study. To be noted, the value of outcome that was from one specific original study in the included meta-analyses were not included and presented in the current review (e.g., parents’ discussion with children about bullying, children’s empathy, feeling safe at school) [20, 42], as meta-analysis requires at least two studies to be statistically combined [43, 44].

ZWL, RL, and XDQ independently screened titles and abstracts during the initial searching phase, followed by a comprehensive full-text review to identify eligible studies.

### Data extraction and quality assessment

Data were extracted independently by the same three researchers using a standardized Excel form (see Additional file 1: Tables S6  and S7 for details). Extracted data encompassed study characteristics, including first author, year of publication, type of intervention, type of outcome, number of included original studies, number of databases searched, number of participants included (intervention group/ control group), date range of databases search, and range of publication year of included meta-analyses. In addition, statistical information was extracted, comprising effect estimates, confidence intervals, prediction intervals, and level of heterogeneity.

A Measurement Tool to Assess Systematic Reviews Version 2 (AMSTAR-2) [45] was used to evaluate the quality of included meta-analyses by the same authors independently. This instrument includes 16 items with responses as no, partial yes (in some items), and yes for each item. In the current umbrella review, we rated the quality of included meta-analyses as high, moderate, low, or critically low based on 7 critical items [45]. The details of measured items and quality assessment standards are shown in Additional file 1: Table S8 [45].

Any discrepancies in the process of literature searching, study selection, data extraction, and quality assessment were resolved through consensus discussion with LL.

### Analysis of the degree of overlap in studies

The degree of overlap among primary studies was calculated using the Corrected Covered Area (CCA), with overlap defined as slight (CCA = 0–5%), moderate (6–10%), high (11–15%), and very high (>15%) [46]; the formula is: $CCA=\frac{N-r}{rc-r}$. N represents the total number of primary studies from all included meta-analyses (including duplicate counting), r (rows) denotes the number of unique primary studies after excluding duplicates, and c (columns) is the number of included meta-analyses.

To further assess overlap and identify review pairs with high overlap [39], we calculated the proportion of primary studies from one review that were present in another review and excluded those with primary study overlap exceeding 40%. In addition, CCA can be evaluated in subsets, i.e., comparing overlap between only two reviews [39].

### Data summary

Following JBI guidance for umbrella reviews, no reanalyzes of effect estimates were attempted [32]. Consistent with the approach employed in one previous study [47], we identified the type of prevention intervention (prevention intervention subgroup and outcome subgroup) in terms of both how the meta-analysis authors named it and our own categorization. Three authors (ZWL, RL, and XDQ) assessed the credibility of evidence for effect estimates reported in the meta-analyses by applying the GRADE criteria (Grading of Recommendations, Assessment, Development, and Evaluations) [48], which encompasses five domains: (1) risk of bias in individual studies, (2) inconsistency, (3) indirectness, (4) imprecision, and (5) publication bias [49]. In addition, three criteria were proposed to enhance credibility: (1) substantial effect estimates without plausible confounders, (2) a dose-response relationship, and (3) a conclusion that all plausible residual confounding would further support inferences regarding the exposure effect [49]. We graded the strength of evidence as high, moderate, low, or very low.

To ensure consistency and facilitate direct comparisons [50], all reported effect estimates from the included meta-analyses were converted to Cohen’s d [15, 38] using the following conversion protocols: 1) for studies reported MD, we first calculated the Cohen’s d (via Campbell Collaboration, www.campbellcollaboration.org/calculator) using means, standard deviations, and sample sizes of each included original studies [51], and the pooled Cohen’s d for the corresponding meta-analysis was aggregated from the Cohen’s d from each of the original studies; 2) for studies reported pooled RR, it was first converted to OR and then to Cohen’s d [43, 44] via Campbell Collaboration; 3) for studies reported pooled OR, it was directly transformed to Cohen’s d [43] via Campbell Collaboration; 4) for studies reported pooled Hedges’ g, it was converted to Cohen’s d using existing formula, i.e., $Hedges{\prime} g=Cohen{\prime} s\,d\times (1-\frac{3}{4({n}_{1}+{n}_{2})-9})$ [52]; and 5) for studies reported pooled SMD, we referred it to Cohen’s d [52, 53].

In our study, pooled Cohen’s d < 0 indicated an improvement or alleviation of the negative bullying behaviors, negative attitudes, or mental health problems by the prevention intervention, while a positive value of Cohen’s d indicated a deterioration of measured outcomes (apart from self-control and social functioning that with a positive value indicates beneficial effectiveness). To ensure consistency, we multiplied effect estimates by −1 in some included studies, where a prevention intervention was beneficial and was originally presented with a value greater than 0 [54, 55]. In addition, we identified inconsistency and corrected the direction of reported effect estimates or their 95% confidence intervals (95% CI) for children’s depression and negative parenting in one study [20], which was confirmed with the first author of that study via email.

When meta-analyses reported effect estimates for both end of intervention and follow-up [15, 56], both short-term and long-term [57], or both involving untreated controls (Effect size 1, ES1) and involving either a treated control or no control (Effect size 2, ES2) [27], we displayed the effect estimates for the end of intervention, short-term and ES1 in forest plot, the complete versions are presented in Additional file 1: Tables S6 and S7. Given that RCTs yield the highest level of evidentiary certainty, we performed a subgroup summary restricted exclusively to RCTs. Sensitivity analyses were conducted by stratifying the AMSTAR-2 quality ratings (High & Moderate vs. Low & Critically Low; and four groups separately), to evaluate the robustness of the primary findings. Forest plots were generated to comprehensively visualize the study results usings R version 4.4.1 via RStudio.

## Results

### Study characteristics

**Fig. 1:**
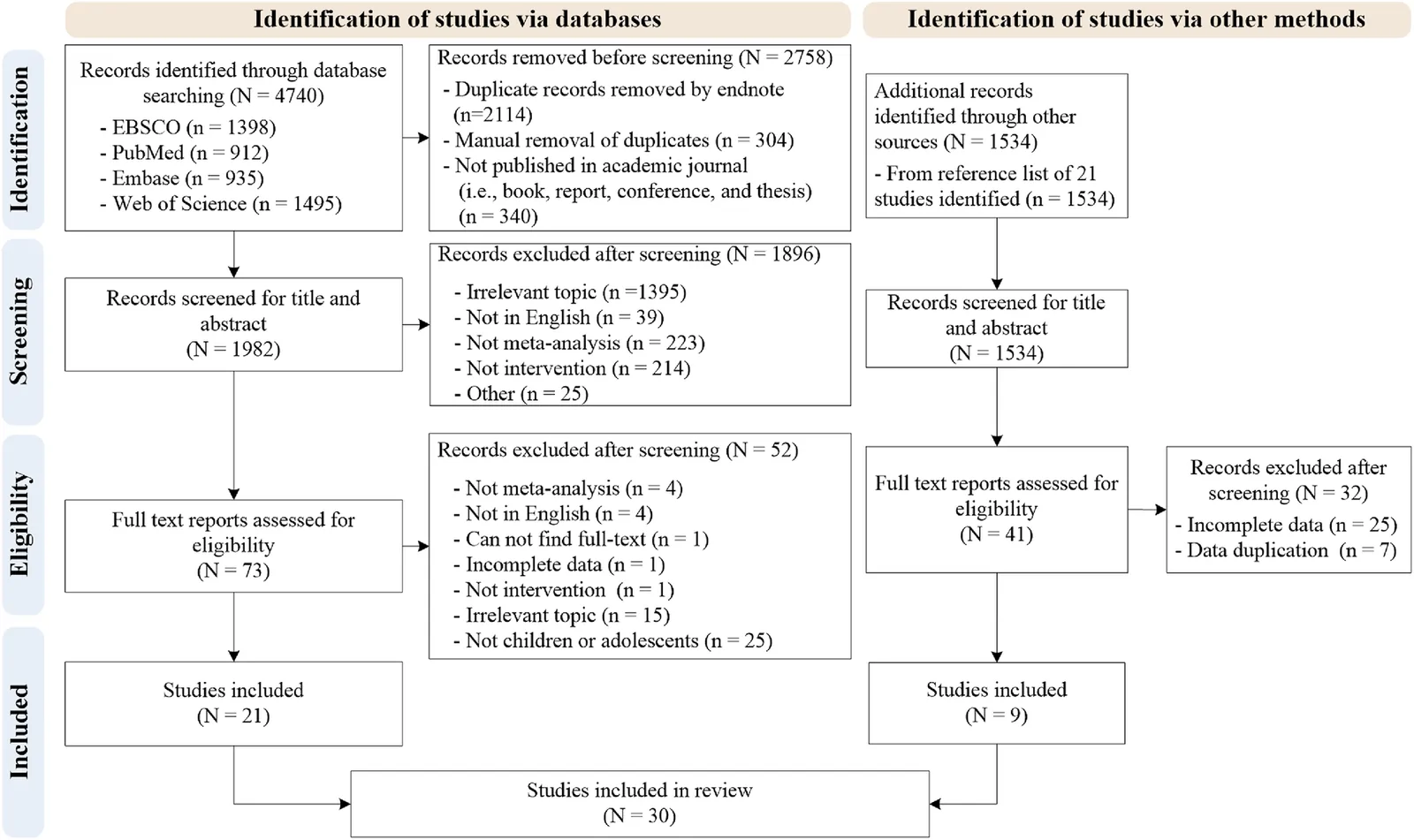
Flowchart of study selection

**Fig. 2:**
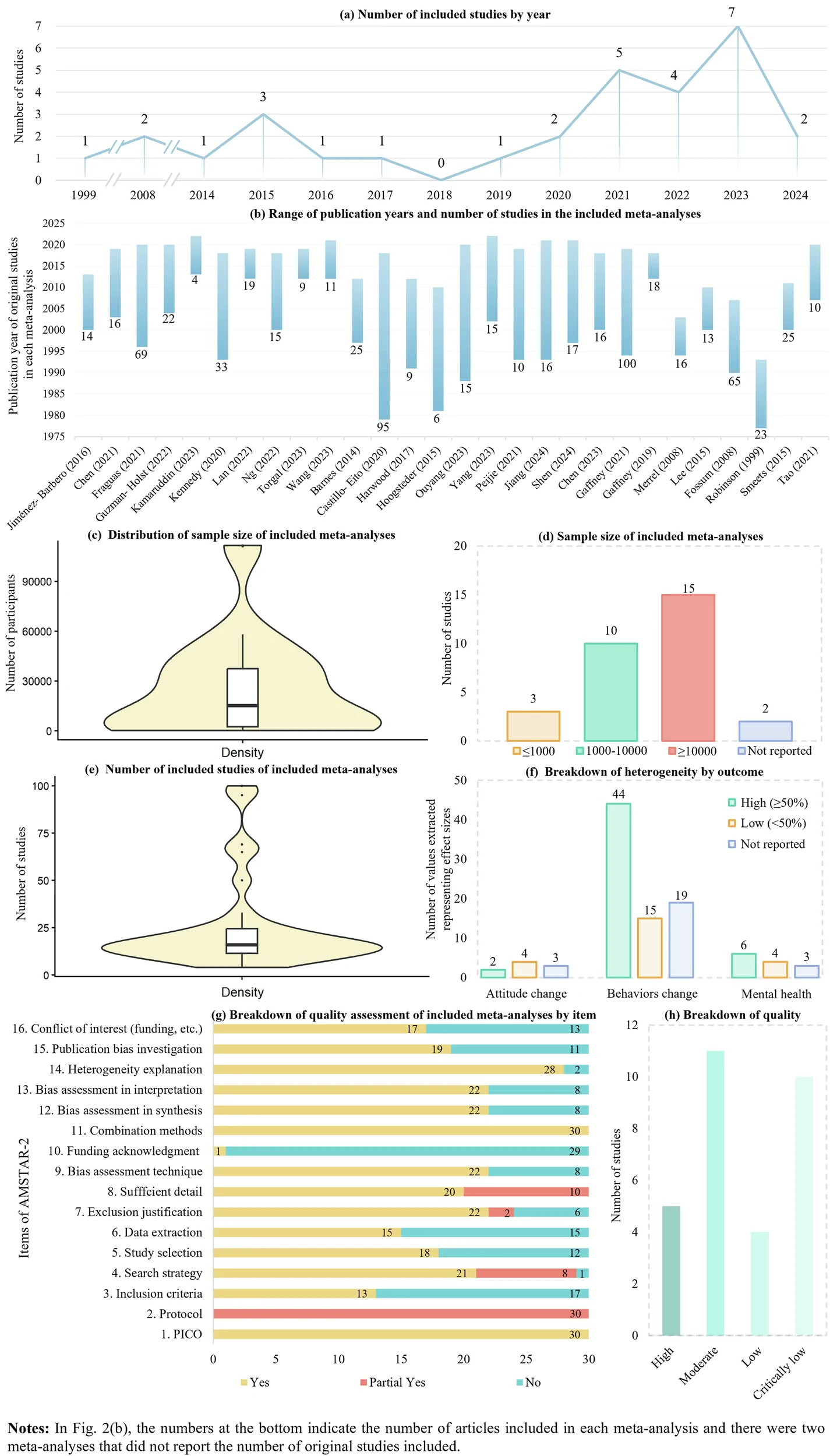
Descriptions and quality assessment of included meta-analyses

A total of 4740 articles were identified through initial searching, 1982 studies remained after removing the duplicates and papers not published in academic journals. After screening titles and abstracts, the full texts of 73 articles were assessed for inclusion, yielding 21 meta-analyses. By screening the reference lists of the 21 studies, 9 meta-analyses were additionally identified (Fig. 1). The overall degree of overlap between reviews was slight (CCA = 0.695%). The degree of overlap across all subsets was less than 40%, and no meta-analyses were excluded on the basis of overlapping primary studies. We included 30 quantitative meta-analyses (Table 1) incorporating 766 original studies for this umbrella review. The included meta-analyses were published between 1999 and 2024, and the original studies included in them dated from 1977 to 2022, with the most extensive time span observed between 1979–2018 [28]. The number of participants in the included meta-analyses of the present study ranged from 164 to 111,659, with half of the included meta-analyses involving over 10,000 participants (Fig. 2), and the age range of participants was 2–23 years (Additional file 1: Table S6).

**Table 1:** Characteristics of the 30 included meta-analyses

No.	Study (Year of publication)	Name of intervention	Name of outcome	Number of studies	Number of sources searched	Sample of participants (I /C)	Date range of databases search	Range of publication year of included original studies
1	Jiménez- Barbero (2016) [26]	Anti-bullying school programs	Bullying or school violence frequency, victimization frequency, attitudes favoring bullying or school violence, attitudes against bullying or school violence, school climate	14	7 databases (Medline, Tripdatabase, Cochrane, Academy Search Premier, PsycINFO, ERIC, and PsycARTICLES)	30,934 (16,243/14,691)	2000.1.1–2015.5.31	2000–2013
2	Chen (2021) [20]	Parenting programs	Overall bullying, children’s depression, parents’ discussion with children about bullying, children’s empathy, positive parenting, negative parenting	16	7 databases (ERIC, MEDLINE, PsycINFO, Sociological Abstracts, Social Service Abstracts, and PubMed)	46,361	Up to 2019.3	2003–2019
3	Fraguas (2021) [15]	School anti-bullying interventions	Overall bullying, bullying perpetration, bullying exposure, cyberbullying, attitudes that discourage bullying, attitudes that encourage bullying, mental health problems, school climate	69	3 databases (Ovid MEDLINE, ERIC [Eric.ed.gov], and PsycInfo)	111,659	Up to 2020.2	1996–2020
4	Guzman- Holst (2022) [61]	Antibullying interventions	Overall internalizing symptoms, depression, anxiety	22	9 databases (PsycINFO, Web of Science, ERIC, SCOPUS, CINAHL, Medline, Embase, ProQuest, and Cochrane Librar)	58,091	1983.1–2021.5	2004–2020
5	Hensums (2023) [58]	School‑based anti‑bullying interventions	Bullying victimization, bullying perpetration	10	4 databases (PsycINFO, Medline, Web of Science, and ERIC)	39,793	Up to 2019.1	NA
6	Kamaruddin (2023) [93]	Cyberbullying intervention	Cyberbullying perpetration frequency, cyberbullying victimization frequency	4	10 databases (Cambridge Journal Online, EBSCOhost, ERIC, IEEE XPLORE, Oxford Journal Online, ProQuest Dissertations and Theses, PubMed [Medline], Science Direct, Scopus, and SpringerLink)	3273 (1802/1471) 2954 (1623/1331)	1995–2022	2013–2022
7	Kennedy (2020) [62]	Bullying prevention programs	Relational bullying victimization, physical bullying victimization, verbal bullying victimization	33	3 databases (PsycInfo, Academic Search Ultimate, and ERIC)	NA	1990–2018	1993–2018
8	Lan (2022) [57]	Anti-cyberbullying educational programs	Cyber aggression, cyber victimization	19	17 databases (Academic Search Complete, Business Source Complete, British Education Index, Communication & Mass Media Complete, Criminal Justice Abstracts, CINAHL Plus, Educational Resource Information Center [ERIC], MEDLINE, Australian Education Index [AEI], PsycARTICLES, PsycINFO, Sociological abstracts, Education Database, Scopus, Cochrane Library, PubMed, and Web of Science)	31,924	Up to 2020.3.31	2012–2019
9	Ng (2022) [23]	Educational interventions	Bullying victimization frequency, bullying perpetration frequency, cyberbullying victimization frequency, cyberbullying perpetration frequency	15	6 databases (PubMed, Embase, PsycINFO, Cumulative Index to Nursing and Allied Health Literature, Google Scholar, and ProQuest Dissertations and Theses)	35,694	Up to 2019.6.30	2000–2018
10	Torgal (2023) [67]	School-based cyberbullying prevention programs	Cyber-bystander behavior	9	17 databases (Academic Search Complete, Education Full Text, ERIC, National Criminal Development Services Group, Inc., Justice Reference Service Abstracts, ProQuest Criminal Justice, ProQuest Dissertations and Theses, ProQuest Education Journals, ProQuest Social Science Journals, PsycINFO, PubMed [Medline], Social Sciences Abstracts, Australian Education Index, British Education Index, CBCA Education, Canadian Research Index, and the Bibliography of Nordic Criminology)	2434	Up to 2019.8	2012–2019
11	Wang (2023) [56]	Parent-related interventions	Cyberbullying victimization frequency, cyberbullying perpetration frequency	11	7 databases (EBSCO, ERIC, PubMed, PsycINFO, Scopus, Web of Science, and ProQuest Dissertations and Theses)	15,143 14,716	Up to 2021.5.27	2012–2021
12	Polanin (2022) [94]	Cyberbullying prevention and intervention programming	Cyberbullying perpetration, cyberbullying victimization, bullying perpetration frequency, bullying victimization	50	15 databases (Academic Search Complete, Education Full Text, ERIC, National Criminal Justice Reference Service Abstracts, ProQuest Criminal Justice, ProQuest Dissertations and Theses, ProQuest Education Journals, ProQuest Social Science Journals, PsycINFO, PubMed [Medline], and Social Sciences Abstracts, CrimDoc, Grey Literature Database [Canadian], Social Care Online [UK], and the Social Science Research Network eLibrary)	45,371	Up to 2019.8.5	NA
13	Barnes (2014) [22]	Cognitive behavioral interventions	Aggressive behaviors	25	7 databases (PsycNet [PsycInfo], JSTOR, ERIC in CSA, Education Full Text [Wilson Web], GALE, EBSCOhost, and GoogleScholar)	30,309	1992–2012	1997–2012
14	Castillo- Eito (2020) [28]	Psychosocial interventions	Aggression	95	3 databases (Web of Science, Scopus and PsycINFO)	111,151 (57,742/ 53,409)	Up to 2019.1	1979–2018
15	Harwood (2017) [14]	Martial arts	Problematic externalizing behavior	9	3 databases (PsycNET, Google Scholar and Web of Science)	507	1980–2015.11	1991–2012
16	Hoogsteder (2015) [60]	Individually oriented cognitive behavioral treatment	Overall several aggressive problems	6	10 databases (PsychINFO, MEDLINE, ERIC, Picarta, International Bibliografy of Social Sciences, Adlib, Sciencedirect, Springerlink, Proquest Dissertation abstracts, and Google Scholar)	164	1980–2011	1981–2010
17	Ouyang (2023) [95]	Physical activity interventions	Total aggression by others-report, total aggression by self-report, physical aggression, verbal aggression, anger, hostility, indirect aggression	15	8 databases (PubMed, PsycINFO, Embase, Web of Science, Ovid, Wan Fang, CNKI, and VIP)	2003	Up to 2020.6	1988–2020
18	Yang (2023) [16]	Sports intervention	Overall aggression, hostility, anger	15	5 databases (Pubmed, Web of Science, Cochrane library, Embase and Scopus)	1632 (833/799)	Up to 2022.10.12	2002–2022
19	Ye (2021) [59]	Music-based intervention	Overall aggression, aggressive behavior, self-control, aggression propensity, hyperactivity- impulsivity, prosocial behavior	10	3 databases (PubMed [MEDLINE], Ovid-Embase, and the Cochrane Central Register of Controlled Trials)	3465	Up to 2020.3	1993–2019
20	Jiang (2024) [21]	Community-based programs	Aggression	16	12 databases (EBSCO, ERIC, PubMed, PsycINFO, MEDLINE, EMBASE, Scopus, Web of Science, ProQuest Dissertations and Theses, the China National Knowledge, Wanfang Databases, and China Science and Technology Journal Database)	2585 (1386/1199)	Up to 2023.7.7	1993–2021
21	Shen (2024) [66]	School-based programs	Aggressive behaviors	17	12 databases (EBSCO, ERIC, PubMed, PsycINFO, MEDLINE, EMBASE, Scopus, Web of Science, ProQuest Dissertations and Theses, China Knowledge Network Database of Journals and Dissertations, Wanfang Database of Journals and Dissertations, and Wipo Database of Journals)	5124 (3565/2564)	Up to 2022.10.14	1997–2021
22	Chen (2023) [63]	Digital health interventions	Overall bullying, cyberbullying, bullying, combined bullying, cyberbullying perpetration, bullying perpetration, cyberbullying victimization, bullying victimization, cyberbullying bystander, bullying bystander, bullying bully–victim dual roles	16	6 databases (PsycINFO, Social Service Abstracts, Sociological Abstracts, MEDLINE, ERIC, and EMBASE)	55,473	Up to 2021.1.31	2000–2018
23	Gaffney (2021) [40]	School‐based programs	Bullying perpetration, bullying victimization	100	7 databases (Web of Science, PscyhINFO, EMBASE, DARE, ERIC, Google Scholar, and Scopus)	NA	NA	1994–2019
24	Gaffney (2019) [96]	Cyberbullying intervention and prevention programs	Cyberbullying perpetration, cyberbullying victimization	18	4 databases (Web of Science, Scopus, PsychINFO, PsychARTICLES, Google Scholar, DARE, ERIC, and ProQuest)	36,708	2000–2017.12	2012–2018
25	Merrel (2008) [42]	School Bullying Intervention Programs	Bullying others, positive attitude toward bullying, being bullied, witnessed bullying, intervene to stop bullying, bullies were talked to by adult, teacher action/response, ignore/refuse to join bullying, reported bullying or likely to, sympathy for victims, positive interactions with peers, staff appropriate responses to bullying, identified victims, identified aggressors	16	2 databases (PsycINFO and ERIC)	15,386	1980–2004	1994–2003
26	Lee (2015) [97]	School-based anti-bullying programs	Bullying victimization	13	5 databases (MEDLINE, PsycInfo, PubMed, the Education Resource Information Center, and the Cochrane Database)	19,619	1990.1–2010.1	2000–2010
27	Fossum (2008) [27]	Psychosocial interventions	Aggressive behaviors, aggressive behaviors	65	1 database (PsychINFO)	4971	1987–2008.1	1990–2007
28	Robinson (1999) [86]	Cognitive behavior modification	Hyperactivity-impulsivity and aggression, hyperactivity-impulsivity, aggression	23	3 databases (ERIC, PsycLIT, and Dissertation Abstracts)	1132	1967–1995	1977–1993
29	Smeets (2015) [85]	Cognitive behavior therapy	Aggressive behaviors	25	4 databases (PubMed, PsycINFO, and EMBASE)	2302	2000.1–2013.4	2000–2011
30	Tao (2021) [65]	Mindfulness-based interventions	Aggressive	10	4 databases (PsycINFO, CINAHL, MEDLINE, and Web of Science)	706	Up to 2020.6.23	2007–2020

Twenty-nine meta-analyses synthesized the respective effect estimates of their included original studies, and one used an individual participant data (IPD) meta-analytic approach [58]. Twelve out of the thirty studies used Cohen’s d to report the effect estimates, five with Hedges’ g, seven with SMD, four with OR, one using MD, and one incorporated both RR and SMD. We extracted 100 values representing prevention intervention outcomes and their 95% CIs (one study with 14 values without reporting 95% CI [42]) representing effect estimates. There were 2 meta-analyses without restrictions on study designs, and 23 of thirty meta-analyses were experimental or quasi-experimental studies or RCTs. We found 5 meta-analyses restricted solely to randomized clinical trials (RCTs) or cluster RCTs (c-RCTs) [15, 23, 26] (Additional file 1: Table S6), and another meta-analysis did not provide confidence intervals of included studies but provided statistical significance [42]. Additional file 1: Table S7 displays the originally reported effect estimates of included meta-analyses.

### Quality and credibility of included meta-analyses

Quality ratings based on AMSTAR-2 criteria revealed substantial heterogeneity, with nearly half of the 30 studies rated as low quality, 11 (36.67%) as moderate, and 5 of 30 (16.67%) as high quality (Additional file 1: Table S9; Fig. 2(g) and Fig. 2(h)). The most commonly unmet critical items (more than 50%) were the absence of explicit inclusion criteria (56.67% [17/30]) and the lack of funding acknowledgment (96.67% [29/30]).

There was large heterogeneity across primary studies, of the 100 effect estimates extracted by outcome variables, 52 (52.00%) exhibited high heterogeneity (*I²* ≥ 50%), 23 (23.00%) showed low heterogeneity (*I²* < 50%), and 25 (25.00%) had no reported heterogeneity. The behavior change domain had the highest heterogeneity, with 56.41% (44/78) of effect estimates showing high heterogeneity, compared with 22.22% (2/9) in attitude change and 46.15% (6/13) in mental health (Fig. 2(f)). Based on information availability, 15 included meta-analyses reported no publication bias, 6 reported the presence of publication bias and 9 were not assessed for publication bias. Among the 23 experimental or quasi-experimental studies or RCTs, only 7 reported information on blinding.

GRADE assessment of the credibility of evidence for the 100 effect estimates revealed that the majority (53/100) were supported by very low strength of evidence, while the remaining effect estimates were supported by low (27/100), moderate (17/100), and high strength of evidence (3/100), respectively (Additional file 1: Table S10).

### Main findings

**Fig. 3:**
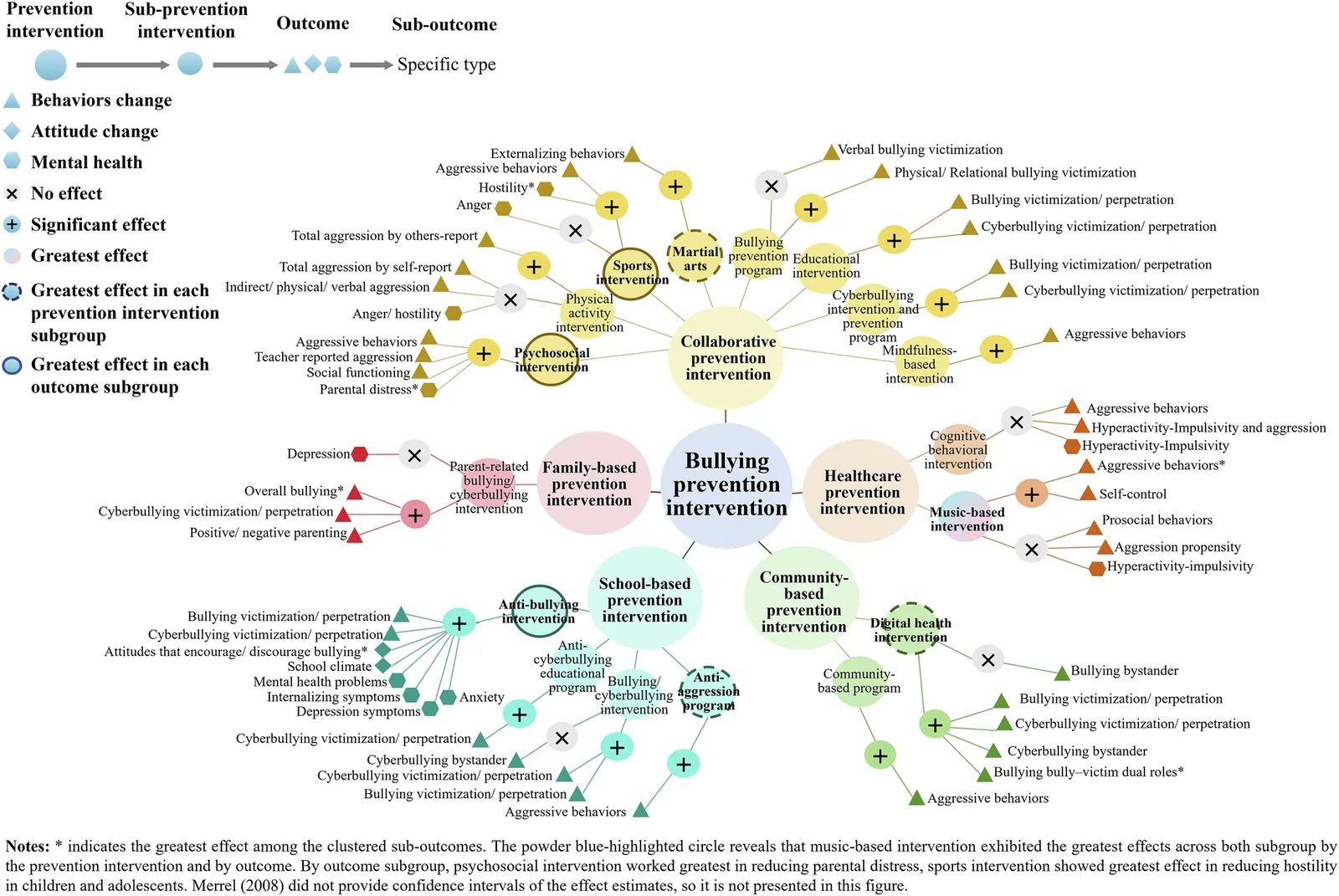
Outcomes and bullying prevention interventions of included meta-analyses

The included meta-analyses mainly evaluated 17 bullying prevention interventions of 49 distinct sub-outcomes, which we systematically summarized as outcomes with three perspectives, i.e., behavior change (73.5%, 36/49), mental health (18.4%, 9/49), and attitude change (8.2%, 4/49). Most studies have focused on assessing behavior change, such as bullying victimization, bullying perpetration and aggressive behaviors. In addition, the prevention intervention programs of included meta-analyses were also characterized by a role-oriented design, which were grouped into five types, namely, family-based (two studies), school-based (ten), community-based (two), healthcare (five), and collaborative prevention interventions (eleven). Given the heterogeneity across existing studies, coupled with the prevalence of ill-defined implementation processes, inconsistent classifications and ambiguous descriptions, the same three authors independently categorized the clustered collections of prevention interventions, with discrepancies resolved through discussion with a psychiatrist. The details of the classification of bullying prevention interventions and outcomes are illustrated in Fig. 3, Additional file 1: Fig. S1 and Table S11.

**Fig. 4:**
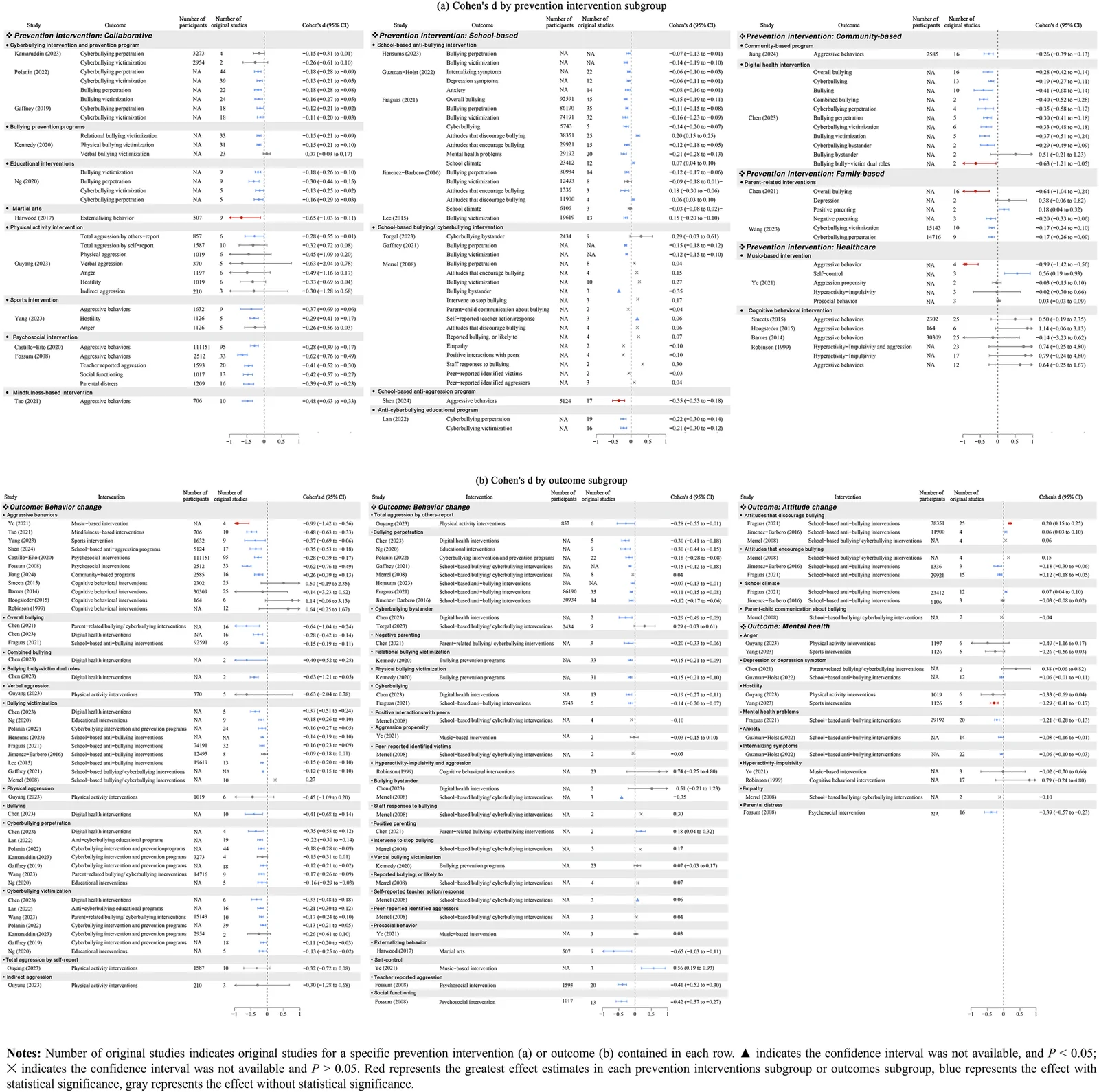
Forest plots of the effectiveness of bullying prevention interventions (a) or outcomes (b)

The included meta-analyses that presented statistical significance all reported beneficial effects of bullying prevention interventions, indicating that these bullying prevention interventions were associated with positive behavior and attitude change, and mental health improvement (Figs. 3 and 4); otherwise, the effect was not statistically significant. The summarized Cohen’s d for the included meta-analyses varied greatly, ranging from −0.99 (−1.42 to −0.56) [59] to 1.14 (−0.06 to 3.13) [60]. Studies reporting collaborative prevention interventions demonstrated the largest varieties on sub-interventions and sub-outcomes, with school-based prevention interventions followed. The original effect measures without transformation by prevention interventions and outcomes are presented in Additional file 1: Tables S12 and S13.

The details of effectiveness in subgroups by prevention intervention are displayed in Fig. 4a, Additional file 1: Tables S14a (complete version) and S14b (simplified version, consistent with forest plot) [14, 20, 27, 28, 40, 54, 55, 61–65]. Among all prevention interventions, the music-based intervention exhibited the largest observed point estimate (Cohen’s d: −0.99, 95% CI: −1.42 to −0.56), which was also the largest among healthcare prevention interventions considering the five collections of prevention interventions. Regarding collaborative prevention interventions, martial arts demonstrated the largest observed point estimate (Cohen’s d: −0.65, 95% CI: −1.03 to −0.11). In terms of community-based and school-based prevention interventions, digital health intervention (Cohen’s d: −0.63, 95% CI: −1.21 to −0.05), and anti-aggression program (Cohen’s d: −0.35, 95% CI: −0.53 to −0.18) presented the largest observed point estimate, respectively. All subgroups of school-based prevention interventions reported small estimates with Cohen’s d ranging from −0.35 (95% CI: −0.53 to −0.18) [66] to 0.29 (95% CI: −0.03 to 0.61) [67]. In view of family-based prevention intervention, parent-related intervention exhibited notable effectiveness in addressing overall bullying with a Cohen’s d of −0.64 (95% CI: −1.04 to −0.24). We did not find statistically significant estimate of cognitive behavioral interventions in any perspective.

Figure 4b, Additional file 1: Tables S15a (complete version) and S15b (simplified version, consistent with Fig. 4b) [14, 20, 27, 28, 40, 54, 55, 61–65] show the details of effectiveness in sub-outcomes. Considering the outcome categories, the music-based intervention exhibited the largest observed point estimate in mitigating aggressive behaviors in the collection of behavior change (Cohen’s d: −0.99). Martial arts demonstrated the second-largest observed point estimate in behavior change, specifically in reducing externalizing behaviors (Cohen’s d: −0.65, 95% CI: −1.03 to −0.11). And parent-related bullying/ cyberbullying interventions represented the third-largest observed point estimate in behavior change, specifically in reducing overall bullying (Cohen’s d: −0.64, 95% CI: −1.04 to −0.24). In view of attitude change, school-based anti-bullying intervention exhibited the largest observed point estimate, specifically in promoting attitudes in discouraging bullying (Cohen’s d: 0.20, 95% CI: 0.15 to 0.25) and encouraging bullying (Cohen’s d: −0.18, 95% CI: −0.30 to −0.06). With respect to mental health outcomes, the sports intervention showed the largest observed point estimate among children and adolescents in mitigating hostility (Cohen’s d: −0.29, 95% CI: −0.41 to −0.17), followed by school-based anti-bullying intervention (Cohen’s d: −0.21, 95% CI: −0.28 to −0.13). In addition, psychosocial interventions exhibited beneficial effects especially in decreasing parental distress (Cohen’s d: −0.39, 95% CI: −0.57 to −0.23). School-based bullying/ cyberbullying intervention did not demonstrate statistically significant effect on improving staff responses to bullying [42].

In addition, subgroup summaries of RCT-only meta-analyses are presented in Additional file 1: Figs. S2 and S3. Across all prevention interventions, educational interventions showed the most pronounced observed point estimate (Cohen’s d = −0.30, 95% CI: −0.44 to −0.15), which was also the largest among collaborative prevention interventions. Regarding school-based prevention interventions, the school-based anti-bullying intervention showed the greatest observed point estimate (Cohen’s d = −0.21, 95% CI: (−0.28 to −0.13). In terms of outcome categories, educational interventions produced the largest observed point estimate in mitigating bullying perpetration in behavior-change outcomes (Cohen’s d = −0.30, 95% CI: −0.44 to −0.15). In view of attitude change, school-based anti-bullying interventions exhibited the largest observed point estimate, specifically in promoting attitudes in discouraging bullying (Cohen’s d: 0.20, 95% CI: 0.15 to 0.25).

### Sensitivity analyses

Details on the effects of prevention interventions stratified by AMSTAR-2 quality ratings (High & Moderate vs. Low & Critically Low) are presented in Additional file 1: Figs. S4 and S5. Among studies with high and moderate AMSTAR-2 ratings, music-based, digital health, anti-aggression, and parent-related interventions consistently exhibited the largest observed point estimates in healthcare-, community-, school-, and family-based prevention interventions, respectively. While mindfulness-based intervention yielded the largest observed point estimate in collaborative prevention interventions (Cohen’s d: −0.48, 95% CI: −0.63 to −0.33). Among studies with Low & Critically Low ratings, martial arts and the school-based anti-bullying intervention exhibited the largest observed point estimates in collaborative and school-based prevention interventions, respectively.

Additional file 1: Figs. S6 and S7 show the effects on sub-outcomes stratified by quality ratings (High & Moderate vs. Low & Critically Low). Among studies with high or moderate ratings, with respect to mitigating aggressive behaviors or attitude change, the music-based intervention and school-based anti-bullying intervention robustly exhibited the largest observed point estimate, respectively. And school-based anti-bullying intervention showed the largest observed point estimate in mitigating mental health problems among children and adolescents (Cohen’s d: −0.21, 95% CI: −0.28 to −0.13). Among studies with Low & Critically Low ratings, martial arts and sports intervention exhibited the largest observed point estimates. Results by stratifying the AMSTAR-2 quality ratings with four groups are presented in Additional file 1: Figs. S8-S15.

## Discussion

This umbrella review included 30 meta-analyses on evaluating the effectiveness of bullying prevention interventions among children and adolescents, synthesizing data from 766 original studies spanning around four decades. We extracted information on assessing 49 distinct sub-outcomes from 17 bullying prevention interventions and found that the included meta-analyses with statistical significance all reported beneficial effects of bullying prevention interventions, improving positive behavior and attitude change, and mental health outcomes. In addition, the existing prevention interventions can be summarized as family-based (two studies), school-based (ten), community-based (two), healthcare (five), and collaborative prevention interventions (eleven). In general, we found evidence suggesting possible effectiveness of several bullying prevention interventions, with variations in effectiveness across subgroups. The pooled Cohen’s d ranged from −0.99 (−1.42 to −0.56) [59] to 1.14 (−0.06 to 3.13) [60]. Results derived from RCT-only subgroup on the efficacy of school-based anti-bullying interventions in improving attitude change were consistent with the main findings.

Our findings indicated that the music-based intervention showed the largest observed point estimate among the identified prevention interventions, specifically in mitigating aggressive behaviors in the collection of behavior change, which aligned with part of findings from pilot trial [68]. Notably, the music-based intervention was rated as moderate quality according to AMSTAR-2, with low credibility of evidence. While the primary findings were generally robust and consistent with those from sensitivity summary by study quality, cautious interpretation of these results is warranted, given the low credibility of the underlying evidence base. As one of the post-interventions of bullying exposure, the music-based intervention is an emerging form of psychotherapy involving music-assisted relaxation, music and imagery, guided imagery, etc. [59], with groove can be treated as a psychological construct and model system [69]. Compared with other prevention interventions, the potential superiority of music-based interventions may be attributed to their multifaceted action mechanisms and high practical feasibility. This advantage drives not from music being a “panacea”, but from its capacity to concurrently modulate multiple mechanisms underlying bullying, including improving social interaction [68], neurological rehabilitation [70], neural encoding of speech [71], brain circuits [72], physical and mental health [73], and reducing internalizing symptoms [74], which may help to reduce the risk of bullying.

While prior studies examined the violence prevention interventions in general population and found beneficial effect of sports-based and anti-bullying interventions targeting children and youth [29], our study summarized the effectiveness of bullying prevention interventions towards children and adolescents and found the largest observed point estimates for martial arts, music-based intervention, digital health intervention, anti-aggression program, and parent-related intervention among the five prevention interventions, respectively. These prevention interventions may intervene bullying from different perspectives. Specifically, martial arts empower participants by channeling energy into self-enhancing power [75], with benefits rooted in self-reflection, ancient philosophies, and breathing techniques [14, 76]. Importantly, martial arts offer a cost-effective intervention, requiring only one instructor for groups of up to 15 participants, while also providing “street-cred” [14]. It should be noted that studies investigating martial arts interventions were characterized by low methodological quality and low evidence credibility, which yielded inconsistent findings between the primary analyses and sensitivity analyses. Correspondingly, in the RCT-only subgroup, educational interventions exhibited the most pronounced observed point estimate among collaborative prevention interventions. These interventions may improve adolescents’ knowledge, practical competence and mental wellbeing to sustain intervention effects [23, 77]. However, this subgroup was only with two available RCTs synthesizing educational interventions [23] and psychosocial interventions [28]. Digital health interventions utilize technology to provide accessible and scalable anti-bullying resources [63], such as webpage-based information and mobile applications [78], which can help to reduce bullying incidence through self-directed learning by osmosis and foster sustainable development among children and adolescents [63]. Despite moderate methodological rigor and evidence credibility, the results of this intervention aligned with the sensitivity analysis, suggesting a degree of robustness. Anti-aggression programs directly target behavioral change through structured strategies, making them highly effective in school [66]. It is important to note that evidence supporting the superiority of anti-aggression program was derived exclusively from high-quality studies with low evidence credibility, which may reduce the robustness of this conclusion. Parent-related interventions could equip caregivers with tools to support their children’s social and emotional development [20]. This highlights that broader, systemic interventions may be important for improving interpersonal relationships, adapting school climate and institutional policies, and potentially generating sustainable long-term outcomes at both individual and societal levels.

Previous assessments on outcomes of bullying prevention interventions mainly focused on evaluating behavior change, attitude change, and mental health. Notably, we found that most bullying prevention interventions primarily target behavior change as a core focus. This underscores that bullying, as a form of unwanted aggressive behavior [2], may be effectively addressed through interventions targeting behavior change, given its actionable and measurable merit. The largest observed point estimates of bullying prevention interventions varied across subgroups, since they targeted unique risk factors and demands within each subgroup. Specifically, we found that music-based intervention exhibited the largest observed point estimate in the subgroup of behavior change. Compared to other prevention interventions, music-based intervention involving only few teachers for a limited period of time, and therefore less expensive but efficient music programs could make music training more accessible for schools [59]. Regarding attitude change, school-based anti-bullying interventions exhibited the largest observed point estimate in discouraging bullying behaviors [15], aimed at imparting cognitive-emotional [79] and social skills [80], improving bystanders’ and bullies’ emotion regulation and increasing empathy for victims [79, 81]. This was supported by findings from both RCT-only subgroup and high-quality studies with high evidence credibility, which further reinforced the robustness of this evidence. Given that children and adolescents spend most of their waking hours in school, anti-bullying interventions may help foster anti-bullying attitudes through continuous and systematic education [81]. In addition, we also found school-based prevention interventions exhibited beneficial values but with relatively small effect estimates, which was consistent with existing study [29]. Sports intervention showed the largest observed point estimate in mitigating hostility related to mental health [16]. However, the evidence supporting this intervention is of low quality with low credibility of evidence, and thus the findings should be interpreted with caution. The social values embedded in the sports such as fair competition and team spirit, could help to foster personal development and fulfilment [82], stimulate the brain to release dopamine [83] and directly alleviate mental health burden [84].

Cognitive behavioral intervention (CBI), involving in behavioral principles, behavior therapy, and cognitive mediation through self-talk, aimed to decrease students’ aggressive behavior [22], while our study did not find beneficial effects in any domain of outcomes [22, 60, 85, 86]. According to previous studies, CBI could decrease adverse effects at the individual level like pain following spinal fusion surgery [87], improve depressed and substance-abusing [88], or anxiety symptoms [89]. However, bullying is a multifaceted problem rooted in complex social dynamics, power imbalances, and environmental factors [2]. For example, bullying involves group behaviors, peer pressure, and social hierarchies [90, 91] that are not easily addressed through individual-focused interventions like CBI. The practical importance of a prevention intervention depends on its cost-benefit ratio [92]. Therefore, to optimize the allocation of resources aimed at alleviating bullying, future studies may consider prioritizing other bullying prevention interventions.

### Limitations

Our study extended the existing literature by comprehensively overview the bullying prevention interventions aiming to promote positive behavior and attitude change, and mental health status. However, some limitations should be acknowledged. First, although inconsistent classifications and descriptions were partly addressed by independent categorization of the prevention interventions by three authors and classifying outcomes and interventions from different aspects, the definitions of bullying prevention interventions and related outcomes were extremely broad, which may still lead to imprecise classification. Second, the specific categories for subgroup stratification were not pre-defined in the registered protocol and these subgroup comparisons are exploratory in nature, which should be interpreted with caution and serve as hypotheses for future research rather than providing confirmatory evidence. Third, methodological differences, such as variations in the statistical models employed for data synthesis across the included meta-analyses, should be considered when conducting cross-study comparisons. Although we converted the extracted effect estimates to Cohen’s d, direct comparisons across bullying prevention interventions should be interpreted with caution, arising from differences in the targeted population (e.g., individuals exposed to bullying vs. the general child and adolescent population), assessment timing (e.g., pre-intervention vs. post-intervention), and high between-study heterogeneity. Consequently, the effectiveness of bullying prevention interventions should be contextualized within the specific types of prevention strategies and outcome categories. Lastly, the study was limited in its power to examine the nuanced details embedded within both the meta-analyses and the original studies; for example, key moderators and follow-up time spans were not further explored.

## Conclusions

In conclusion, bullying prevention interventions may foster behavior change, attitude change, or mental health improvement. Music-based interventions showed the largest observed point estimates, followed by martial arts, parent-related interventions, digital health interventions, and mindfulness-based interventions. The effectiveness of bullying prevention interventions varied across prevention intervention subgroups and outcome subgroups, with RCT-derived evidence supporting robust benefits of school-based anti-bullying interventions in promoting attitude change. This umbrella review provides timely evidence to summarize the effectiveness of various prevention interventions for bullying in a scalable and standardized way, laying guidance for future bullying prevention programs.

## Supplementary information

Additional File 1 **Figures S1-S15**. Fig. S1-[The relationship between bullying prevention intervention and related outcome]. Fig. S2-[The efficacy by prevention intervention subgroups in studies with RCT only]. Fig. S3-[The efficacy by outcome subgroups in studies with RCT only]. Fig. S4-[high or moderate AMSTAR-2 quality rating (prevention intervention subgroups)]. Fig. S5-[low or critically low AMSTAR-2 quality rating (prevention intervention subgroups)]. Fig. S6-[high or moderate AMSTAR-2 quality rating (outcome subgroups)]. Fig. S7-[low or critically low AMSTAR-2 quality rating (outcome subgroups)]. Fig. S8-[high AMSTAR-2 rating (prevention intervention subgroups)]. Fig. S9-[moderate AMSTAR-2 rating (prevention intervention subgroups)]. Fig. S10-[low AMSTAR-2 rating (prevention intervention subgroups)]. Fig. S11-[critically low AMSTAR-2 rating (prevention intervention subgroups)]. Fig. S12-[high AMSTAR-2 rating (outcome subgroups)]. Fig. S13-[moderate AMSTAR-2 rating (outcome subgroups)]. Fig. S14-[low AMSTAR-2 rating (outcome subgroups)]. Fig. S15-[critically low AMSTAR-2 rating (outcome subgroups)]. **Additional File 1: Tables S1-S15**. Table S1-[PRISMA checklist]. Table S2-[Modifications or new addition to the original protocol]. Table S3-[Search strategy]. Table S4-[Examples of excluded meta-analyses]. Table S5-[Examples of details of one excluded and one included meta-analysis]. Table S6-[Specific characteristics of included meta-analyses]. Table S7-[Original effect sizes and related values of included meta-analyses]. Table S8-[16-items of AMSTAR-2]. Table S9-[Results of AMSTAR-2 quality assessment]. Table S10-[GRADE assessment]. Table S11-[Classifications of bullying prevention interventions and outcomes]. Table S12-[The original effect measures without transformation by prevention interventions]. Table S13-[The original effect measures without transformation by outcomes]. Table S14a-[Cohen’s d by prevention intervention subgroup (Complete version)]. Table S14b-[Cohen’s d by prevention intervention subgroup (Simplified version, consistent with forest plot)]. Table S15a-[Cohen’s d by outcome subgroup (Complete version)]. Table S15b-[Cohen’s d by outcome subgroup (Simplified version, consistent with forest plot)]

## Data Availability

This study is an umbrella review and no original data were generated. All data analyzed in this study were obtained from publicly available sources, specifically PubMed, Web of Science, Embase, and EBSCO. These databases are accessible through institutional or individual subscriptions.

## Abbreviations

AMSTAR-2: A Measurement Tool to Assess Systematic Reviews Version 2
CBI: Cognitive behavioral intervention
CCA: Corrected Covered Area
CI: Confidence interval
GRADE: Grading of Recommendations, Assessment, Development, and Evaluations
IPD: Individual participant data
JBI: Joanna Briggs Institute
MD: Mean difference
NA: Not applicable
NR: Not reported
OR: Odds ratios
PRISMA: Preferred Reporting Items for Systematic Reviews and Meta-Analyses
PROSPERO: International Prospective Register of Systematic Reviews
RCT: Randomized clinical trials
RR: Relative risk
SCT: Social cognitive theory
SMD: Standardized mean difference
WHO: World Health Organization
WoS: Web of Science
