# Supplementary material for: Effectiveness of bullying prevention-associated interventions among children and adolescents: an umbrella review of systematic review and meta-analysis
Source: BMC Med. 2026 Jun 17;24:464. doi: 10.1186/s12916-026-04974-x (PMC13520287; doi:10.1186/s12916-026-04974-x)
Supplement: Supplementary file 1 [file 12916_2026_4974_MOESM1_ESM.docx]

**Effectiveness of Bullying Prevention-Associated Interventions among Children and Adolescents: An Umbrella Review of Systematic Review and Meta-analysis**

**Zi-Wei Li, PhD^1^; Li Lu, PhD^1,2*^; Xiao-Dong Qin, MD^1^; Rong Li, MD^1^; Zhongliang Zhou, PhD^1^; Shou Liu, PhD^3^; Qi Liu, PhD^4^; Sha Lai, PhD^1^; Qing Shen, PhD^5,6,7^**

**Additional file 1**

**Supplemental Information**

| **Fig. S1** | The relationship between bullying prevention intervention and related outcome |
| --- | --- |
| **Fig. S2** | The efficacy by prevention intervention subgroups in studies with RCTs only |
| **Fig. S3** | The efficacy by outcome subgroups in studies with RCTs only |
| **Fig. S4** | The effectiveness by prevention intervention subgroups in studies with high or moderate AMSTAR-2 quality rating |
| **Fig. S5** | The effectiveness by prevention intervention subgroups in studies with low or critically low AMSTAR-2 quality rating |
| **Fig. S6** | The effectiveness by outcome subgroups in studies with high or moderate AMSTAR-2 quality rating |
| **Fig. S7** | The effectiveness by outcome subgroups in studies with low or critically low AMSTAR-2 quality rating |
| **Fig. S8** | The effectiveness by prevention intervention subgroups in studies with high AMSTAR-2 rating |
| **Fig. S9** | The effectiveness by prevention intervention subgroups in studies with moderate AMSTAR-2 rating |
| **Fig. S10** | The effectiveness by prevention intervention subgroups in studies with low AMSTAR-2 rating |
| **Fig. S11** | The effectiveness by prevention intervention subgroups in studies with critically low AMSTAR-2 rating |
| **Fig. S12** | The effectiveness by outcome subgroups in studies with high AMSTAR-2 rating |
| **Fig. S13** | The effectiveness by outcome subgroups in studies with moderate AMSTAR-2 rating |
| **Fig. S14** | The effectiveness by outcome subgroups in studies with low AMSTAR-2 rating |
| **Fig. S15** | The effectiveness by outcome subgroups in studies with critically low AMSTAR-2 rating |
| **Table S1** | PRISMA checklist |
| **Table S2** | Modifications or new additions to the original protocol |
| **Table S3** | Search strategy |
| **Table S4** | Examples of excluded meta-analyses |
| **Table S5** | Examples of details of one excluded and one included meta-analysis |
| **Table S6** | Specific characteristics of included meta-analyses |
| **Table S7** | Original effect sizes and related values of included meta-analyses |
| **Table S8** | 16-items of AMSTAR-2 |
| **Table S9** | Results of AMSTAR-2 quality assessment |
| **Table S10** | GRADE assessment of the credibility of evidence |
| **Table S11** | Classifications of bullying prevention interventions and outcomes |
| **Table S12** | The original effect measures without transformation by prevention interventions |
| **Table S13** | The original effect measures without transformation by outcomes |
| **Table S14a** | Cohen's d by prevention intervention subgroup (Complete version) |
| **Table S14b** | Cohen's d by prevention intervention subgroup (Simplified version, consistent with forest plot) |
| **Table S15a** | Cohen's d by outcome subgroup (Complete version) |
| **Table S15b** | Cohen's d by outcome subgroup (Simplified version, consistent with forest plot) |

# Fig. S1. The relationship between bullying prevention interventions and related outcomes

# Fig. S2. The efficacy by prevention intervention subgroups in studies with RCTs only

# Fig. S3. The efficacy by outcome subgroups in studies with RCTs only

# Fig. S4. The effectiveness by prevention intervention subgroups in studies with high or moderate AMSTAR-2 quality rating

# Fig. S5. The effectiveness by prevention intervention subgroups in studies with low or critically low AMSTAR-2 quality rating

# Fig. S6. The effectiveness by outcome subgroups in studies with high or moderate AMSTAR-2 quality rating

# Fig. S7. The effectiveness by outcome subgroups in studies with low or critically low AMSTAR-2 quality rating

# Fig. S8. The effectiveness by prevention intervention subgroups in studies with high AMSTAR-2 rating

# Fig. S9. The effectiveness by prevention intervention subgroups in studies with moderate AMSTAR-2 rating

# Fig. S10. The effectiveness by prevention intervention subgroups in studies with low AMSTAR-2 rating

# Fig. S11. The effectiveness by prevention intervention subgroups in studies with critically low AMSTAR-2 rating

# Fig. S12. The effectiveness by outcome subgroups in studies with high AMSTAR-2 rating

# Fig. S13. The effectiveness by outcome subgroups in studies with moderate AMSTAR-2 rating

# Fig. S14. The effectiveness by outcome subgroups in studies with low AMSTAR-2 rating

# Fig. S15. The effectiveness by outcome subgroups in studies with critically low AMSTAR-2 rating

# Table S1. PRISMA checklist

| **Section and Topic** | **Item #** | **Checklist item** | **Location where item is reported** |
| --- | --- | --- | --- |
| **TITLE** | | |  |
| Title | 1 | Identify the report as a systematic review. | 1 |
| **ABSTRACT** | | |  |
| Abstract | 2 | See the PRISMA 2020 for Abstracts checklist. | 2 |
| **INTRODUCTION** | | |  |
| Rationale | 3 | Describe the rationale for the review in the context of existing knowledge. | 3-5 |
| Objectives | 4 | Provide an explicit statement of the objective(s) or question(s) the review addresses. | 5 |
| **METHODS** | | |  |
| Eligibility criteria | 5 | Specify the inclusion and exclusion criteria for the review and how studies were grouped for the syntheses. | 6-7, 9 |
| Information sources | 6 | Specify all databases, registers, websites, organisations, reference lists and other sources searched or consulted to identify studies. Specify the date when each source was last searched or consulted. | 6 |
| Search strategy | 7 | Present the full search strategies for all databases, registers and websites, including any filters and limits used. | 6, Table S3 |
| Selection process | 8 | Specify the methods used to decide whether a study met the inclusion criteria of the review, including how many reviewers screened each record and each report retrieved, whether they worked independently, and if applicable, details of automation tools used in the process. | 6-8, Table S4 & S5 |
| Data collection process | 9 | Specify the methods used to collect data from reports, including how many reviewers collected data from each report, whether they worked independently, any processes for obtaining or confirming data from study investigators, and if applicable, details of automation tools used in the process. | 8 |
| Data items | 10a | List and define all outcomes for which data were sought. Specify whether all results that were compatible with each outcome domain in each study were sought (e.g. for all measures, time points, analyses), and if not, the methods used to decide which results to collect. | 8, Table S6 & S7 |
|  | 10b | List and define all other variables for which data were sought (e.g. participant and intervention characteristics, funding sources). Describe any assumptions made about any missing or unclear information. | 8, Table S6 & S7 |
| Study risk of bias assessment | 11 | Specify the methods used to assess risk of bias in the included studies, including details of the tool(s) used, how many reviewers assessed each study and whether they worked independently, and if applicable, details of automation tools used in the process. | 8-9, Table S8 |
| Effect measures | 12 | Specify for each outcome the effect measure(s) (e.g. risk ratio, mean difference) used in the synthesis or presentation of results. | 10 |
| Synthesis methods | 13a | Describe the processes used to decide which studies were eligible for each synthesis (e.g. tabulating the study intervention characteristics and comparing against the planned groups for each synthesis (item #5)). | 11, Table S6 & S7 |
|  | 13b | Describe any methods required to prepare the data for presentation or synthesis, such as handling of missing summary statistics, or data conversions. | 10-11 |
|  | 13c | Describe any methods used to tabulate or visually display results of individual studies and syntheses. | 11 |
|  | 13d | Describe any methods used to synthesize results and provide a rationale for the choice(s). If meta-analysis was performed, describe the model(s), method(s) to identify the presence and extent of statistical heterogeneity, and software package(s) used. | 10-11 |
|  | 13e | Describe any methods used to explore possible causes of heterogeneity among study results (e.g. subgroup analysis, meta-regression). | 11 |
|  | 13f | Describe any sensitivity analyses conducted to assess robustness of the synthesized results. | 11 |
| Reporting bias assessment | 14 | Describe any methods used to assess risk of bias due to missing results in a synthesis (arising from reporting biases). | 8, Table S8 |
| Certainty assessment | 15 | Describe any methods used to assess certainty (or confidence) in the body of evidence for an outcome. | 9-10 |
| **RESULTS** | | |  |
| Study selection | 16a | Describe the results of the search and selection process, from the number of records identified in the search to the number of studies included in the review, ideally using a flow diagram. | 11-12, Figure 1 |
|  | 16b | Cite studies that might appear to meet the inclusion criteria, but which were excluded, and explain why they were excluded. | 7, Table S4 & S5 |
| Study characteristics | 17 | Cite each included study and present its characteristics. | 11-12, Table 1 & Table S5 |
| Risk of bias in studies | 18 | Present assessments of risk of bias for each included study. | 12-13, Figure 2 |
| Results of individual studies | 19 | For all outcomes, present, for each study: (a) summary statistics for each group (where appropriate) and (b) an effect estimate and its precision (e.g. confidence/credible interval), ideally using structured tables or plots. | 13-16, Figure 3 & 4; Fig. S1, Table S12-S15 |
| Results of syntheses | 20a | For each synthesis, briefly summarise the characteristics and risk of bias among contributing studies. | 12-13, Figure 2 |
|  | 20b | Present results of all statistical syntheses conducted. If meta-analysis was done, present for each the summary estimate and its precision (e.g. confidence/credible interval) and measures of statistical heterogeneity. If comparing groups, describe the direction of the effect. | 13-16, Figure 3 & 4; Fig. S1, Table S12-S15 |
|  | 20c | Present results of all investigations of possible causes of heterogeneity among study results. | 13-17 |
|  | 20d | Present results of all sensitivity analyses conducted to assess the robustness of the synthesized results. | 17, Fig. S4 & S5 |
| Reporting biases | 21 | Present assessments of risk of bias due to missing results (arising from reporting biases) for each synthesis assessed. | Table S6 |
| Certainty of evidence | 22 | Present assessments of certainty (or confidence) in the body of evidence for each outcome assessed. | 12-13, Table S10 |
| **DISCUSSION** | | |  |
| Discussion | 23a | Provide a general interpretation of the results in the context of other evidence. | 18-22 |
|  | 23b | Discuss any limitations of the evidence included in the review. | 22-23 |
|  | 23c | Discuss any limitations of the review processes used. | 23 |
|  | 23d | Discuss implications of the results for practice, policy, and future research. | 23-24 |
| **OTHER INFORMATION** | | |  |
| Registration and protocol | 24a | Provide registration information for the review, including register name and registration number, or state that the review was not registered. | 5 |
|  | 24b | Indicate where the review protocol can be accessed, or state that a protocol was not prepared. | 5 |
|  | 24c | Describe and explain any amendments to information provided at registration or in the protocol. | 5, Table S2 |
| Support | 25 | Describe sources of financial or non-financial support for the review, and the role of the funders or sponsors in the review. | 26 |
| Competing interests | 26 | Declare any competing interests of review authors. | 27 |
| Availability of data, code and other materials | 27 | Report which of the following are publicly available and where they can be found: template data collection forms; data extracted from included studies; data used for all analyses; analytic code; any other materials used in the review. | 26 |

**Notes:** NA: Not applicable; Source of the table (32).

# Table S2. Modifications or new additions to the original protocol

| **Section** | **Original protocol** | **Modifications or new addition** | **Rationale** |
| --- | --- | --- | --- |
| Study selection | 2.Studies involving children and adolescents aged 24 years or younger. | Studies involving bullying behaviors in children (<18 years) and adolescents, the latter was defined as individuals aged 10-24 years since it was noted that this age range aligns more closely with adolescent growth and popular understandings of this life phase, which could facilitate extended investments across diverse contexts (36). Children were defined as individuals aged <18 years (37). | For accurate characterisation of the research endpoint, we restricted the population to bullying behaviours occurring in children (<18 years) and adolescents (10-24 years). |
| Study selection | 6.Articles providing odds ratios (OR) or metrics convertible to ORs (e.g., mean, standard deviation (SD), standardized mean difference (SMD), or Cohen's d) will be included. | (f) providing Cohen's d or other measures [e.g., mean difference (MD), relative risk (RR), odds ratios (OR), Hedges' g, standardized mean difference (SMD)] that can be converted to Cohen's d, since it was widely reported in existing reviews (15, 38) | Drawing on established high-impact studies (15, 38), all non-Cohen’s d effect sizes were converted to this standardized metric. This approach was implemented to facilitate cross-study comparisons and minimize potential biases arising from the transformation of heterogeneous effect size metrics. |
| Analysis of the degree of overlap in studies | NR | The degree of overlap among primary studies was calculated using the Corrected Covered Area (CCA).  To further assess overlap and identify reviews pairs with high overlap (39), we calculated the proportion of primary studies from one review that were present in another review and excluded those with primary study overlap exceeding 40%. | To avoid double-counting and overestimation of evidence |
| Data summary | NR | Three authors (ZWL, RL, and XDQ) assessed the credibility of evidence for effect estimate reported in the meta-analysis by applying the GRADE criteria (Grading of Recommendations, Assessment, Development, and Evaluations). | To rate the confidence of effect estimates |
| Data summary | NR | Sensitivity analyses were conducted by stratifying the AMSTAR-2 quality ratings (High & Moderate VS. Low & Critically Low; and four groups separately), to evaluate the robustness of the primary findings. | To enhance the robustness of results |
| **References** | | | |
| 15. Fraguas D, Díaz-Caneja CM, Ayora M, Durán-Cutilla M, Abregú-Crespo R, Ezquiaga-Bravo I, et al. Assessment of School Anti-Bullying Interventions: A Meta-analysis of Randomized Clinical Trials. JAMA Pediatr. 2021;175(1):44-55.  36. Sawyer SM, Azzopardi PS, Wickremarathne D, Patton GC. The age of adolescence. Lancet Child Adolesc Health. 2018;2(3):223-8.  37. UNICEF. Convention on the Rights of the Child General Assembly resolution 44/25; 1989.  38. Baldwin JR, Wang B, Karwatowska L, Schoeler T, Tsaligopoulou A, Munafò MR, et al. Childhood Maltreatment and Mental Health Problems: A Systematic Review and Meta-Analysis of Quasi-Experimental Studies. Am J Psychiatry. 2023;180(2):117-26. | | | |

**Notes:** NR: Not reported.

# Table S3. Search strategy

| **Database** | **Search Strategy** |
| --- | --- |
| PubMed | **Total: 2739 913 (ZWL); 913 (RL); 913 (XDQ) After duplicate removal: 912**  (child*[Title/Abstract] OR adolescent[Title/Abstract] OR teen*[Title/Abstract] OR youth[Title/Abstract]) AND (bullying[Title/Abstract] OR anti-bullying[Title/Abstract] OR "school bullying"[Title/Abstract] OR cyberbullying[Title/Abstract] OR victimization[Title/Abstract] OR "bullying victimization"[Title/Abstract] OR cyber-victimization[Title/Abstract] OR abuse[Title/Abstract] OR violence[Title/Abstract] OR aggression[Title/Abstract]) AND (intervention[Title/Abstract] OR prevention[Title/Abstract] OR treatment[Title/Abstract] OR therapy[Title/Abstract] OR curriculum[Title/Abstract]) AND ("systematic review"[Title/Abstract] OR meta-analysis[Title/Abstract]) |
| Embase | **Total: 2841 947 (ZWL); 947 (RL); 947 (XDQ) After duplicate removal: 935**  (child*:ab,ti OR adolescent:ab,ti OR teen*:ab,ti OR youth:ab,ti) AND (bullying:ab,ti OR 'anti bullying':ab,ti OR 'school bullying':ab,ti OR cyberbullying:ab,ti OR victimization:ab,ti OR 'bullying victimization':ab,ti OR 'cyber victimization':ab,ti OR abuse:ab,ti OR violence:ab,ti OR aggression:ab,ti) AND (intervention:ab,ti OR prevention:ab,ti OR treatment:ab,ti OR therapy:ab,ti OR curriculum:ab,ti) AND ('systematic review':ab,ti OR 'meta analysis':ab,ti) |
| Web of science | **Total: 4530 1510 (ZWL); 1510 (RL); 1510 (XDQ) After duplicate removal: 1495**  AB=((child* OR adolescent OR teen* OR youth) AND (bullying OR anti-bullying OR “school bullying” OR cyberbullying OR victimization OR “bullying victimization” OR cyber-victimization OR abuse OR violence OR aggression) AND (intervention OR prevention OR treatment OR therapy OR curriculum) AND (“systematic review” OR meta-analysis)) |
| EBSCO ^#^ | **Total: 4801 1488 (ZWL); 1557 (RL); 1756 (XDQ) After duplicate removal: 1398**  AB=((child* OR adolescent OR teen* OR youth) AND (bullying OR anti-bullying OR “school bullying” OR cyberbullying OR victimization OR “bullying victimization” OR cyber-victimization OR abuse OR violence OR aggression) AND (intervention OR prevention OR treatment OR therapy OR curriculum) AND (“systematic review” OR meta-analysis)) |

**Notes:** ^#^ The EBSCO database has a function of “exact duplicates removed from the results”. Thus, the number of studies searched out by the three authors was inconsistent.

# Table S4. Examples of excluded meta-analyses

| **No** | **Year** | **Authors** | **Title** | **Journal** |
| --- | --- | --- | --- | --- |
| 1 | 2009 | Farrington D P, Ttofi M M | School-Based Programs to Reduce Bullying and Victimization | Campbell systematic reviews |
| 2 | 2007 | Ferguson C J, Miguel C S, Kilburn Jr J C,  Sanchez, P | The Effectiveness of School-Based Anti-Bullying Programs: A Meta-Analytic Review | Criminal Justice Review |
| 3 | 2019 | Gaffney H,  Ttofi M M,  Farrington D P | Evaluating the effectiveness of school-bullying prevention programs: An updated meta-analytical review | Aggression and violent behavior |
| 4 | 2019 | Huang Y,  Espelage D L, Polanin J R,  Hong, J. S | A meta-analytic review of school-based anti-bullying programs with a parent component | International journal of bullying prevention |
| 5 | 2011 | Ttofi M M, Farrington D P | Effectiveness of school-based programs to reduce bullying: A systematic and meta-analytic review | Journal of experimental criminology |
| 6 | 2021 | Gaffney H,  Ttofi M M, Farrington D P | What works in anti-bullying programs? Analysis of effective intervention components | Journal of school psychology |
| 7 | 2019 | Gaffney H,  Farrington D P,  Ttofi M M | Examining the effectiveness of school-bullying intervention programs globally: A meta-analysis | International Journal of Bullying Prevention |
| **Reasons for exclusion** | | | | |
| - Five articles from the same team ^1-5^, two of which are updated versions ^2-3^ of previous articles ^1,5^, one ^4^ used the data from ^3^. Thus, we included the newest meta-analyses [40]. - No list of articles reporting specific incorporation ^7^. - 15 of 22 included studies ^7^ were included in Gaffney et al (2021) [40]. | | | | |
| **References** | | | | |
| 1. Farrington DP and Ttofi MM (2009) School-based programs to reduce bullying and victimization. Campbell systematic reviews 5(1), i-148. 2. Gaffney H, Farrington DP and Ttofi MM (2019) Examining the effectiveness of school-bullying intervention programs globally: A meta-analysis. International Journal of Bullying Prevention 1, 14-31. 3. Gaffney H, Ttofi MM and Farrington DP (2019) Evaluating the effectiveness of school-bullying prevention programs: An updated meta-analytical review. Aggression and Violent Behavior 45, 111-133. 4. Gaffney H, Ttofi MM and Farrington DP (2021) What works in anti-bullying programs? Analysis of effective intervention components. Journal of school psychology 85, 37-56. 5. Ttofi MM and Farrington DP (2011) Effectiveness of school-based programs to reduce bullying: A systematic and meta-analytic review. Journal of experimental criminology 7, 27-56. 6. Ferguson CJ, Miguel CS, Kilburn Jr JC and Sanchez P (2007) The effectiveness of school-based anti-bullying programs: A meta-analytic review. Criminal Justice Review 32(4), 401-414. 7. Huang Y, Espelage DL, Polanin JR and Hong JS (2019) A meta-analytic review of school-based anti-bullying programs with a parent component. International Journal of Bullying Prevention 1, 32-44. | | | | |

# **Table S5.** Examples of details of one excluded and one included meta-analysis

| **Reference List** | |
| --- | --- |
| **Excluded** | **Included in the current study** |
| **Huang et al. (2019) (41)** | **Gaffney et al (2021) (40)** |
| [1] Alsaker, F. D., & Valkanover, S. (2012). The Bernese program against victimization in kindergarten and elementary school. New Directions for Youth Development, 2012(133), 15-28. [2] **Bauer, N. S., Lozano, P., & Rivara, F. P. (2007). The effectiveness of the Olweus Bullying Prevention Program in public middle schools: a controlled trial. Journal of Adolescent Health, 40(3), 266-274.** [3] **Beran, T. N., Tutty, L., & Steinrath, G. (2004). An evaluation of a bullying prevention program for elementary schools. Canadian Journal of School Psychology, 19(1-2), 99-116.** [4] **Berry, K., & Hunt, C. J. (2009). Evaluation of an intervention program for anxious adolescent boys who are bullied at school. Journal of Adolescent Health, 45(4), 376-382.** [5] Crean, H. F., & Johnson, D. B. (2013). Promoting alternative thinking strategies (PATHS) and elementary school aged children's aggression: Results from a cluster randomized trial. American Journal of Community Psychology, 52(1/2), 56-72. [6] Cross, D., Shaw, T., Hadwen, K., Cardoso, P., Slee, P., Roberts, C., Thomas L. & Barnes, A. (2016). Longitudinal impact of the cyber friendly schools program on adolescents' cyberbullying behavior. Aggressive Behavior, 42(2), 166-180. [7] Cross, D., Waters, S., Pearce, N., Shaw, T., Hall, M., Erceg, E., . . . Hamilton, G. (2012). The friendly schools friendly families programme: three-year bullying behaviour outcomes in primary school children. International Journal of Educational Research, 53, 394- 406. [8] **Domino, M. B. (2011). The Impact of Take the LEAD on School Bullying Among Middle School Youth (unpublished doctoral dissertation). Walden University.** [9] **Evers, K. E., Prochaska, J. O., Van Marter, D. F., Johnson, J. L., & Prochaska, J. M. (2007). Transtheoretical-based bullying prevention effectiveness trials in middle schools and high schools. Educational Research, 49(4), 397-414.** [10] **Fekkes, M., Pijpers, F.I.M., & Verloove-Vanhorick, S.P. (2006). Effects of antibullying school program on bullying and health complaints. Archives of Pediatrics and Adolescent Medicine, 160, 638-644.** [11] **Frey, K., Hirschstein, M.K., Snell, J. L., van Schoiack Edstrom, L., MacKenzie, E.P., & Broderick, C.J. (2005). Reducing playground bullying and supporting beliefs: an experimental trial of the steps to respect program. Developmental Psychology, 41, 479-491.** [12] **Hunt, C. (2007). The effect of an education program on attitudes and beliefs about bullying and bullying behaviour in junior secondary school students. Child and Adolescent Mental Health, 12(1), 21-26.** [13] **Joronen, K., Konu, A., Rankin, H. S., & Åstedt-Kurki, P. (2011). An evaluation of a drama program to enhance social relationships and anti-bullying at elementary school: a controlled study. Health Promotion International, 27(1), 5-14.** [14] **Kärnä, A., Voeten, M., Little, T. D., Alanen, E., Poskiparta, E., & Salmivalli, C. (2013). Effectiveness of the KiVa Antibullying program: grades 1-3 and 7-9. Journal of Educational Psychology, 105(2), 535, 551.** [15] **Kärnä, A., Voeten, M., Little, T. D., Poskiparta, E., Alanen, E., & Salmivalli, C. (2011). Going to scale: a nonrandomized nationwide trial of the KiVa antibullying program for grades 1-9. Journal of Consulting and Clinical Psychology, 79(6), 796-805.** [16] **Lewis, K. M., Schure, M. B., Bavarian, N., DuBois, D. L., Day, J., Ji, P., Silverthorn, N., Acock, A., Vuchinich, S., & Flay, B. R. (2013). Problem behavior and urban, low-income youth: A randomized controlled trial of Positive Action in Chicago. American Journal of Preventive Medicine, 44(6), 622-630.** [17] Leadbeater, B., & Sukhawathanakul, P. (2011). Multicomponent programs for reducing peer victimization in early elementary school: a longitudinal evaluation of the WITS primary program. Journal of Community Psychology, 39(5), 606-620. [18] **Pepler, D.J., Craig, W.M., O'Connell, P., Atlas, R. & Charach, A. (2004). Making a difference in bullying: evaluation of a systemic school-based program in Canada. In P.K. Smith, D. Pepler, & K. Rigby (Eds.), Bullying in schools: how successful can interventions be? (pp. 125-140). Cambridge: Cambridge University Press.** [19] Raskauskas, J. (2007). Evaluation of the Kia Kaha anti-bullying programme for students in years 5-8. Final Report to the New Zealand Police. Wellington: New Zealand Police. [20] **Salmivalli, C., Kaukiainen, A., & Voeten, M. (2005). Anti-bullying intervention: Implementation and outcome. British Journal of Educational Psychology, 75(3), 465-487.** [21] Stevens, V., De Bourdeaudhuij, I., & Van Oost, P. (2000). Bullying in Flemish schools: an evaluation of anti-bullying intervention in primary and secondary schools. British Journal of Educational Psychology, 70(2), 195-210. [22] **Wong, D. S., Cheng, C. H., Ngan, R. M., & Ma, S. K. (2011). Program effectiveness of a restorative whole-school approach for tackling school bullying in Hong Kong. International Journal of Offender Therapy and Comparative Criminology, 55(6), 846-862.** | **Selected References:**   [1] Alsaker, F. D. (2004). Bernese program against victimization to kindergarten and elementary schools. In P. K. Smith, D. Pepler & K. Rigby (Eds.), Bullying in schools: How successful can interventions be? (pp. 289-306). Cambridge University Press. [2] Alsaker, F. D., & Valkanover, S. (2001). Early diagnosis and prevention of victimization in kindergarten. In J. Juvonen & S. Graham (Eds.), Peer harassment in school (pp. 175-195). Guilford.   .......   [3] **Bauer, N. S., Lozano, P., & Rivara, F. P. (2007). The effectiveness of the Olweus bullying prevention program in public middle schools: A controlled trial. Journal of Adolescent Health, 40, 266-274.** [4] Beran, T., & Shapiro, B. (2005). Evaluation of an anti-bullying program: Student reports of knowledge and confidence to manage bullying. Canadian Journal of Education, 28(4), 700-717. [5] **Beran, T., Tutty, L., & Steinrath, G. (2004). An evaluation of a bullying prevention program for elementary schools. Canadian Journal of School Psychology, 19(1/2), 99-116.** [6] **Berry, K., & Hunt, C. J. (2009). Evaluation of an intervention program for anxious adolescent boys who are bullied at school. Journal of Adolescent Health, 45, 376-382.**   ...........   [7] **Domino, M. (2011). The impact of Take the LEAD on school bullying among middle school youth (Doctoral Dissertation). Available from ProQuest Dissertation and Theses database (UMI No. 3434870).** [8] Domino, M. (2013). Measuring the impact of an alternative approach to school bullying. Journal of School Health, 83(6), 430-437.   ...........   [9] **Evers, K. E., Poskiparta, J. O., van Marter, D. F., Johnson, J. L., & Prochaska, J. M. (2007). Transtheoretical-based bullying prevention effectiveness trials in middle schools and high schools. Educational Research, 49, 397-414.**   ...........   [10] **Fekkes, M., Pijpers, F. I. M., & Verloove-Vanhorick, P. (2006). Effects of antibullying school program on bullying and health complaints Archives of Pediatrics and Adolescent Medicine, 160(6), 638-644** [11] Fekkes, M., van de Sande, M. C. E., Gravesteijn, J. C., Pannebakker, F. D., Buijs, G. J., Diekstra, R. F. W., & Kocken, P. L. (2016). Effects of the Dutch Skills for Life program on the health behaviour, bullying, and suicidal ideation of secondary school students. Health Education, 116(1), 2-15.   ...........   [12] **Frey, K., Hirschstein, M. K., Snell, J. L., van Schoiack Edstrom, L., MacKenzie, E. P., & Broderick, C. J. (2005). Reducing playground bullying and supporting beliefs: An experimental trial of the Steps to Respect program. Developmental Psychology, 41, 479-491.**   ...........   [13] **Hunt, C. (2007). The effect of an education program on attitudes and beliefs about bullying and bullying behaviour in junior secondary school students. Child and Adolescent Mental Health, 12(1), 21-26.**   ...........   [14] **Joronen, K., Konu, A., Rankin, H. S., & Astedt-Kurki, P. (2011). An evaluation of a drama program to enhance social relationships and anti-bullying at elementary school: A controlled study. Health Promotion International, 27(1), 5-14.**   ...........   [15] **Kärnä, A., Voeten, M., Little, T. D., Alanen, E., Poskiparta, E., & Salmivalli, C. (2013). Effectiveness of the KiVa Antibullying Program: Grades 1-3 and 7-9. Journal of Educational Psychology, 105(2), 535-551.** [16] **Kärnä, A., Voeten, M., Little, T. D., Poskiparta, E., Alanen, E., & Salmivalli, C. (2011a). Going to scale: A nonrandomized nationwide trial of the KiVa antibullying Program for Grades 1-9. Journal of Consulting and Clinical Psychology, 79(6), 796-805.** [17] Kärnä, A., Voeten, M., Little, T. D., Poskiparta, E., Kaljonen, A., & Salmivalli, C. (2011b). A large-scale evaluation of the KiVa antibullying program: Grades 4-6. Child Development, 82(1), 311-330.   ...........   [18] **Lewis, K. M., Schure, M. B., Bavarian, N., DuBois, D. L., Day, J., Ji, P., Silverthorn, N., Acock, A., Vuchinich, S., & Flay, B. R. (2013). Problem behavior and urban, low-income youth: A randomized controlled trial of Positive Action in Chicago. American Journal of Preventive Medicine, 44(6), 622-630.**   ...........   [19] **Pepler, D. J., Craig, W. M., O'Connell, P., Atlas, R., & Charach, A. (2004). Making a difference in bullying: Evaluation of a systemic school-based program in Canada. In P. K. Smith, D. Pepler & K. Rigby (Eds.), Bullying in schools: How successful can interventions be? (pp. 125-140). Cambridge University Press.** [20] Polanin, M.K. (2015). Effects of cultural awareness training in conjunction with an established bullying prevention program (Doctoral Dissertation). Loyola University Chicago.   ...........   [21] **Salmivalli, C., Kaukiainen, A., & Voeten, M. (2005). Anti-bullying intervention: Implementation and outcome. British Journal of Educational Psychology, 75, 465-487.**   ...........   [22] **Wong, D. S. W., Cheng, C. H. K., Ngan, R. M. H., & Ma, S. K. (2011). Program effectiveness of a restorative whole-school approach for tackling school bullying in Hong Kong. International Journal of Offender Therapy and Comparative Criminology, 55(6), 846-862.**   ........... |

**Notes:** The bold references indicate that the same studies were included in the two separate meta-analyses. n=number of original studies.

# Table S6. Specific characteristics of included meta-analyses

| **No.** | **First author (year)** | **Name of intervention** | **Model synthesizing data** | **Publication bias** | **Study design** | **Sample of participants** | **Sample of group** | | **N** | **Year of studies** | **Journal** | **Name of outcome variable** | **Age** |
| --- | --- | --- | --- | --- | --- | --- | --- | --- | --- | --- | --- | --- | --- |
|  |  |  |  |  |  |  | **Intervention** | **Control** |  |  |  |  |  |
| 1 | Jiménez-Barbero (2016) | Anti-bullying school programs | Random effects model | Funnel-plot; Presence of publication bias in victimization and school climate; NA | RCT | 30934 | 16243 | 14691 | 14 | 2000.01.01 —2015.05.31 | Children and Youth Services Review | Bullying or school violence frequency | 7-16 |
|  |  |  |  |  |  | 14293 | 7683 | 6610 |  |  |  | Victimization frequency |  |
|  |  |  |  |  |  | 1336 | 660 | 676 |  |  |  | Attitudes favoring bullying or school violence |  |
|  |  |  |  |  |  | 11900 | 5929 | 5971 |  |  |  | Attitudes against bullying or school violence |  |
|  |  |  |  |  |  | 6106 | 2806 | 3300 |  |  |  | School climate |  |
| 2 | Chen (2021) | Parenting programs | Random effects model | Funnel plot, Begg–Mazumdar rank correlation, Egger's correlation tests; No publication bias; Trim and Fill test | RCT and pre-posttest | 46361 | - | - | 16 | —2019.3 | Trauma, Violence, & Abuse | Overall bullying | 4-15 |
|  |  |  |  |  |  |  | - | - |  |  |  | Children's depression |  |
|  |  |  |  |  |  |  | - | - |  |  |  | Positive parenting |  |
|  |  |  |  |  |  |  | - | - |  |  |  | Negative parenting |  |
| 3 | Fraguas (2021) | School anti-bullying interventions | Random effects model | Funnel Plot, fail-safe N, Egger's regression; Presence of publication bias in Overall bullying, Bullying perpetration, Attitudes that discourage bullying, Mental health problems, School climate; NA | RCT | 111659 | 46847 | 45744 | 69 | 2020.2 | JAMA Pediatrics | Overall bullying (E) | 4-17 |
|  |  |  |  |  |  |  | 11020 | 11977 |  |  |  | Overall bullying (F) |  |
|  |  |  |  |  |  |  | 43199 | 42991 |  |  |  | Bullying perpetration (E) |  |
|  |  |  |  |  |  |  | 7889 | 7993 |  |  |  | Bullying perpetration (F) |  |
|  |  |  |  |  |  |  | 37190 | 37001 |  |  |  | Bullying exposure (E) |  |
|  |  |  |  |  |  |  | 6971 | 7629 |  |  |  | Bullying exposure (F) |  |
|  |  |  |  |  |  |  | 3271 | 2472 |  |  |  | Cyberbullying (E) |  |
|  |  |  |  |  |  |  | - | - |  |  |  | Cyberbullying (F) |  |
|  |  |  |  |  |  |  | 20537 | 17778 |  |  |  | Attitudes that discourage bullying (E) |  |
|  |  |  |  |  |  |  | 5517 | 4596 |  |  |  | Attitudes that discourage bullying (F) |  |
|  |  |  |  |  |  |  | 15884 | 14037 |  |  |  | Attitudes that encourage bullying (E) |  |
|  |  |  |  |  |  |  | 3329 | 3299 |  |  |  | Attitudes that encourage bullying (F) |  |
|  |  |  |  |  |  |  | 14543 | 14649 |  |  |  | Mental health problems (E) |  |
|  |  |  |  |  |  |  | 1605 | 1621 |  |  |  | Mental health problems (F) |  |
|  |  |  |  |  |  |  | 11417 | 11995 |  |  |  | School climate (E) |  |
|  |  |  |  |  |  |  | 2647 | 2978 |  |  |  | School climate (F) |  |
| 4 | Guzman- Holst (2022) | Antibullying interventions | Random effects model | Funnel Plot; No publication bias; Duval and Tweedie trim-and-fill procedure | Randomized experiments, experimental-control comparisons with pre- and postmeasures, and other experimental-control comparisons. | 58091 | - | - | 22 | 2011-2020 | Journal of Child Psychology and Psychiatry | Overall internalizing symptoms | 4-19（10.5） |
|  |  |  |  |  |  |  | - | - |  |  |  | Depression |  |
|  |  |  |  |  |  |  | - | - |  |  |  | Anxiety |  |
| 5 | Hensums (2023) | School‑based anti‑bullying interventions | Fitting multilevel regression models | Year of publication, Location of study (by continent), Design, Reported effects; No publication bias; NA | RCT, quasi-experimental, measure before and after interventions | 39793 | - | - | 10 | —2019.1 | Prevention Science | Bullying victimization | 12.58±2.34 |
|  |  |  |  |  |  |  | - | - |  |  |  | Bullying perpetration |  |
| 6 | Kamaruddin (2023) | Game-based, skill-building, school-based, and whole-school interventions | Random-effects model with  robust variance estimation | Searched for relevant information including published and unpublished research reports and available data sets; NA; NA | RCT, quasi-experimental studies, and studies that may have assigned groups in a randomized or non-randomized way to conditions without any exclusion based on the level of assignment | 3273 | 1802 | 1471 | 4 | 1995—2022 | Frontiers in Psychiatry | Cyberbullying perpetration frequency | 8-23 |
|  |  |  |  |  |  | 2954 | 1623 | 1331 |  |  |  | Cyberbullying victimization frequency |  |
| 7 | Kennedy (2020) | Bullying prevention programs | Random effects model | This search included all types of scholarly articles; NA; NA | Experimental, quasi-experimental, non-experimental | - | - | - | 33 | 1990—2018 | Aggression and Violent Behavior | Relational bullying victimization | Under the age of 18 |
|  |  |  |  |  |  | - | - | - |  |  |  | Physical bullying victimization |  |
|  |  |  |  |  |  | - | - | - |  |  |  | Verbal bullying victimization |  |
| 8 | Lan (2022) | Anti-cyberbullying educational programs | Random effects model | Funnel plot, Rosenthal's fail-safe number, fail-safe N; No publication bias; NA | Quasi-experimental and RCTs | 31924 | - | - | 19 | —2020.3.31 | Computers in Human Behavior | Cyber aggression (S) | 11.3-16.8 |
|  |  |  |  |  |  |  | - | - |  |  |  | Cyber aggression (L) |  |
|  |  |  |  |  |  |  | - | - |  |  |  | Cyber victimization (S) |  |
|  |  |  |  |  |  |  | - | - |  |  |  | Cyber victimization (L) |  |
| 9 | Ng (2022) | Educational interventions | Random effects model | Funnel plots; Presence of publication bias in bullying victimization and perpetration; NA | RCTs | 35694 | - | - | 15 | —2019.6.30 | Trauma, Violence, & Abuse | Bullying victimization frequency | 10-18 |
|  |  |  |  |  |  |  | - | - |  |  |  | Bullying victimization frequency |  |
|  |  |  |  |  |  |  | - | - |  |  |  | Bullying perpetration frequency |  |
|  |  |  |  |  |  |  | - | - |  |  |  | Bullying perpetration frequency |  |
|  |  |  |  |  |  |  | - | - |  |  |  | Cyberbullying victimization frequency |  |
|  |  |  |  |  |  |  | - | - |  |  |  | Cyberbullying perpetration frequency |  |
| 10 | Torgal (2023) | School-based cyberbullying prevention programs | Random effects model with robust variance estimation | Not assessed | Quasi-experimental and RCTs | 2434 | - | - | 9 | —2019.8 | School Psychology Review | Cyber-bystander behavior | 10-18 |
| 11 | Wang (2023) | Parent-related interventions | Random effects model | Funnel plot, Egger's test; Presence of publication bias; Duval and Tweedie's trim and fill method | Quasi-experimental and RCTs | 15143 | 8503 | 6640 | 11 | —2021.5.27 | Trauma, Violence, & Abuse | Cyberbullying victimization frequency (E) | 10-19 |
|  |  |  |  |  |  |  | 1819 | 1518 |  |  |  | Cyberbullying victimization frequency (F) |  |
|  |  |  |  |  |  | 14716 | 7100 | 7616 |  |  |  | Cyberbullying perpetration frequency (E) |  |
|  |  |  |  |  |  |  | 1794 | 1488 |  |  |  | Cyberbullying perpetration frequency (F) |  |
| 12 | Polanin (2022) | Cyberbullying prevention and intervention programming | Multilevel, random-effects  model with robust variance estimation | Egger's test, funnel plots; No publication bias; Duval and Tweedie's trim and fill method | NR | 45371 | - | - | 50 | —2019.8.5 | Prevention Science | Cyberbullying perpetration | 13±1.73 |
|  |  |  |  |  |  |  | - | - |  |  |  | Cyberbullying victimization |  |
|  |  |  |  |  |  |  | - | - |  |  |  | Bullying perpetration frequency |  |
|  |  |  |  |  |  |  | - | - |  |  |  | Bullying victimization |  |
| 13 | Barnes (2014) | Cognitive behavioral interventions | Fixed effects model | Rosenthal's formula; No publication bias; NA | Experimental or quasi-experimental | 30309 | - | - | 25 | 1992—2012 | Aggression and Violent Behavior | Aggressive behaviors | 10-17.99 |
| 14 | Castillo-Eito (2020) | Psychosocial interventions | Three-level random-effects meta-analysis | Located grey literature; NA; NA | RCTs | 111151 | 57742 | 53409 | 95 | —2019.1 | Clinical Psychology Review | Aggression | 13.36 (mean age) |
| 15 | Harwood (2017) | Martial arts | Not assessed | Funnel Plot; No publication bias; NA | NR | 507 | - | - | 9 | 1980—2015.11 | Aggression and Violent Behavior | Problematic externalizing behavior | 6-18 |
| 16 | Hoogsteder (2015) | Individually oriented cognitive behavioral treatment | Multilevel random effects model | Fail-safe number; No publication bias; NA | Quasi-experimental | 164 | - | - | 6 | 1980—2011 | The Journal of Forensic Psychiatry & Psychology | Overall several aggressive problems | 12-18 (15.8) |
| 17 | Ouyang (2023) | Physical activity interventions | Random effects model | Egger's tests and funnel plots; No publication bias; NA | Quasi-experimental and RCTs | 2003 | 444 | 413 | 15 | —2020.6 | Aggression and Violent Behavior | Total aggression by others-report | Under the age of 18 |
|  |  |  |  |  |  |  | 807 | 780 |  |  |  | Total aggression by self-report |  |
|  |  |  |  |  |  |  | 517 | 502 |  |  |  | Physical aggression |  |
|  |  |  |  |  |  |  | 187 | 183 |  |  |  | Verbal aggression |  |
|  |  |  |  |  |  |  | 641 | 556 |  |  |  | Anger |  |
|  |  |  |  |  |  |  | 516 | 503 |  |  |  | Hostility |  |
|  |  |  |  |  |  |  | 105 | 105 |  |  |  | Indirect aggression |  |
| 18 | Yang (2023) | Sports intervention | DerSimonian-Laird random effects model or fixed effects  model | Not assessed | Quasi-experimental and RCTs | 1632 | 833 | 799 | 15 | —2022.10.12 | Peer J | Overall aggression | 7-16 |
|  |  |  |  |  |  | 1126 | 571 | 555 |  |  |  | Hostility |  |
|  |  |  |  |  |  | 1126 | 571 | 555 |  |  |  | Anger |  |
| 19 | Ye (2021) | Music-based intervention | Random effects model | Funnel plot, Egger linear regression test; Presence of publication bias in aggressive behavior; NA | Quasi-RCT and RCT | 3465 | - | - | 10 | —2020.3 | Medicine | Aggressive behavior | 8-13 |
|  |  |  |  |  |  |  | - | - |  |  |  | Self-control |  |
|  |  |  |  |  |  |  | - | - |  |  |  | Aggression propensity |  |
|  |  |  |  |  |  |  | - | - |  |  |  | Hyperactivity-impulsivity |  |
|  |  |  |  |  |  |  | - | - |  |  |  | Prosocial behavior |  |
| 20 | Jiang (2024) | Community-based programs | Random effects model | Egger's correlation tests, Begg-Mazumdar rank correlation, funnel plots; No  publication bias; NA | Quasi-experimental and RCTs | 2585 | 1386 | 1199 | 16 | 2023.7.7 | Trauma, Violence, & Abuse | Aggression | 2-18 |
| 21 | Shen (2024) | School-based anti-aggression programs | Random effects model | Funnel plots, Egger's test; No publication bias; Trim and Fill tests | Quasi-experimental and RCTs | 5124 | 3565 | 2564 | 17 | 2022.10.14 | Research on Social Work Practice | Aggressive behaviors | 3-19 |
| 22 | Chen (2023) | Digital health interventions (DHIs) | Random effects model | Funnel plots; No publication bias; Trim and fill tests | Quasi-experimental and RCTs | 55473 | - | - | 16 | 2021.1.31 | Trauma, Violence, & Abuse | Overall bullying | 7-23 |
|  |  |  |  |  |  |  | - | - |  |  |  | Cyberbullying |  |
|  |  |  |  |  |  |  | - | - |  |  |  | Bullying |  |
|  |  |  |  |  |  |  | - | - |  |  |  | Combined bullying |  |
|  |  |  |  |  |  |  | - | - |  |  |  | Cyberbullying perpetration |  |
|  |  |  |  |  |  |  | - | - |  |  |  | Bullying perpetration |  |
|  |  |  |  |  |  |  | - | - |  |  |  | Cyberbullying victimization |  |
|  |  |  |  |  |  |  | - | - |  |  |  | Bullying victimization |  |
|  |  |  |  |  |  |  | - | - |  |  |  | Cyberbullying bystander |  |
|  |  |  |  |  |  |  | - | - |  |  |  | Bullying bystander |  |
|  |  |  |  |  |  |  | - | - |  |  |  | Bullying bully–victim dual roles |  |
| 23 | Gaffney (2021) | School‐based programs | Multivariance adjustment model and random effects model | Funnel plot; No publication bias; Duval and Tweedie's trim | Quasi-experimental and RCTs | - | - | - | 100 | 2009—2016.12 | Campbell Systematic Reviews | Bullying perpetration | 4-18 |
|  |  |  |  |  |  |  | - | - |  |  |  | Bullying victimization |  |
| 24 | Gaffney (2019) | Cyberbullying intervention and prevention programs | Random effects model | Funnel plot, Rosenthal's Fail-Safe N; Presence of publication bias; Duval and Tweedie's trim | Quasi-experimental and RCTs | 36708 | - | - | 18 | —2017.12 | Aggression and Violent Behavior | Cyberbullying perpetration | 11-19 |
|  |  |  |  |  |  |  | - | - |  |  |  | Cyberbullying victimization |  |
| 25 | Merrel (2008) | School bullying intervention programs | Multiplicative variance adjustment model | Gray literature; NA; NA | RCT or quasi-experimental, age cohort | 15386 | - | - | 16 | 1980—2004 | School Psychology Quarterly | Bullying others | 5-16 |
|  |  |  |  |  |  |  | - | - |  |  |  | Positive attitude toward bullying |  |
|  |  |  |  |  |  |  | - | - |  |  |  | Being bullied |  |
|  |  |  |  |  |  |  | - | - |  |  |  | Witnessed bullying |  |
|  |  |  |  |  |  |  | - | - |  |  |  | Intervene to stop bullying |  |
|  |  |  |  |  |  |  | - | - |  |  |  | Bullies were talked to by adult |  |
|  |  |  |  |  |  |  | - | - |  |  |  | Teacher action/response |  |
|  |  |  |  |  |  |  | - | - |  |  |  | Ignore/refuse to join bullying |  |
|  |  |  |  |  |  |  | - | - |  |  |  | Reported bullying, or likely to |  |
|  |  |  |  |  |  |  | - | - |  |  |  | Sympathy for victims |  |
|  |  |  |  |  |  |  | - | - |  |  |  | Positive interactions with peers |  |
|  |  |  |  |  |  |  | - | - |  |  |  | Staff appropriate responses to bullying |  |
|  |  |  |  |  |  |  | - | - |  |  |  | Peer report Identified victims |  |
|  |  |  |  |  |  |  | - | - |  |  |  | Peer report Identified aggressors |  |
| 26 | Lee (2015) | School-based anti-bullying programs | Random effects model | Not assessed | Longitudinal cohort, RCT, controlled trial, posttest only RCT | 19619 | - | - | 13 | 1990.1—2010.1 | Journal of Child Health Care | Bullying victimization | 9-16 |
| 27 | Fossum (2008) | Psychosocial interventions | Random effects model | Not assessed | No control condition | 2512 | - | - | 65 | 1987—2005.8 | European Child & Adolescent Psychiatry | Aggressive behaviours (ES1) | 4-16 |
|  |  |  |  |  |  | 2459 | - | - |  |  |  | Aggressive behaviours (ES2) |  |
|  |  |  |  |  |  | 1593 | - | - |  |  |  | Teacher reported aggression (ES1) |  |
|  |  |  |  |  |  | 653 | - | - |  |  |  | Teacher reported aggression (ES2) |  |
|  |  |  |  |  |  | 1017 | - | - |  |  |  | Social functioning (ES1) |  |
|  |  |  |  |  |  | 670 | - | - |  |  |  | Social functioning (ES2) |  |
|  |  |  |  |  |  | 1209 | - | - |  |  |  | Parental distress (ES1) |  |
|  |  |  |  |  |  | 1169 | - | - |  |  |  | Parental distress (ES2) |  |
| 28 | Robinson (1999) | Cognitive behavior modification | Multiple measures | Rosenthal's formula; No publication bias; NA | Experimental or quasi-experimental | 1132 | - | - | 23 | 1967—1995 | Journal of Educational Psychology | Hyperactivity-Impulsivity and aggression | 5-13 |
|  |  |  |  |  |  |  | - | - |  |  |  | Hyperactivity-Impulsivity |  |
|  |  |  |  |  |  |  | - | - |  |  |  | Aggression |  |
| 29 | Smeets (2015) | Cognitive behavior therapy | Random effects model | Not assessed | RCT | 2302 | - | - | 25 | 2000.1—2013.4 | European Child & Adolescent Psychiatry | Aggressive behaviors | 10.78 |
| 30 | Tao (2021) | Mindfulness-based interventions | Random effects model | Funnel plot, Egger's regression test;No publication bias; Trim and Fill method | RCT, quasi-experiment, or single subject | 706 | - | - | 10 | —2020.6.23 | Mindfulness | Aggression | 9.85 |

**Notes:** E: End of intervention; F: Follow-up; S: Short term; L: Long term; ES1: Studies involving an untreated control; ES2: Studies involving either a treated control or no control; N: Total number of articles; NR: Not reported; NA: Not applicable.

# Table S7. Original effect sizes and related values of included meta-analyses

| **No.** | **First author (year)** | **Name of intervention** |  | **Name of outcome variable** | **n** | **Original effect size** | Cohen's d (95% CI) | **Largest effect size** | **Smallest effect size** | **I²** | **Q** |
| --- | --- | --- | --- | --- | --- | --- | --- | --- | --- | --- | --- |
| 1 | Jiménez-Barbero (2016) | Anti-bullying school programs | **－** | Bullying or school violence frequency | 14 | SMD | -0.12 (-0.17 to -0.06) | 0.01 (-0.23 to 0.25) | -0.80 (-1.40 to -0.20) | 65 | <0.001 |
|  |  |  | 🚫 | Victimization frequency | 8 | SMD | -0.09 (-0.18 to 0.01) | 0.04 (-0.22 to 0.29) | -0.80 (-1.40 to -0.20) | 74 | <0.001 |
|  |  |  | **－** | Attitudes favoring bullying or school violence | 3 | SMD | -0.18 (-0.30 to -0.06) | -0.14 (-0.25 to -0.02) | -0.37 (-0.67 to -0.08) | 4 | 2.09 |
|  |  |  | **＋** | Attitudes against bullying or school violence | 4 | SMD | 0.06 (0.03 to 0.10) | 0.15 (-0.05 to 0.36) | 0.00 (-0.22 to 0.22) | 0 | 1.5 |
|  |  |  | 🚫 | School climate | 3 | SMD | -0.03 (-0.08 to 0.02) | 0.00 (-0.07 to 0.08) | -0.09 (-0.29 to 0.11) | 0 | 1.36 |
| 2 | Chen (2021) | Parenting programs | **－** | Overall bullying | 16 | Cohen's d | 0.640 (0.239 to 1.041) | 2.613 (1.946 to 3.280) | 0.030 (-0.326 to 0.386) | 99.628 | <0.001 |
|  |  |  | 🚫 | Children's depression | 2 | Cohen's d | -0.381 (-0.060 to 0.821)^*^ | - | - | 53.607 | 0.142 |
|  |  |  | **＋** | Positive parenting | 2 | Cohen's d | 0.180 (0.036 to 0.323) | - | - | 0 | 0.389 |
|  |  |  | **－** | Negative parenting | 3 | Cohen's d | -0.195 (0.061 to 0.329)^*^ | - | - | 0 | 0.521 |
| 3 | Fraguas (2021) | School anti-bullying interventions | **－** | Overall bullying (E) | 45 | Cohen's d | -0.150 (-0.191 to -0.109) | -0.013 (-0.060 to 0.034) | -0.807 (-1.324 to -0.291) | 85.3 | <0.001 |
|  |  |  | **－** | Overall bullying (F) | 21 | Cohen's d | -0.171 (-0.243 to -0.099) | - | - | 80 | <0.001 |
|  |  |  | **－** | Bullying perpetration (E) | 35 | Cohen's d | -0.111 (-0.146 to -0.077) | - | - | 78.8 | <0.001 |
|  |  |  | **－** | Bullying perpetration (F) | 17 | Cohen's d | -0.175 (-0.276 to -0.073) | - | - | 85.9 | <0.001 |
|  |  |  | **－** | Bullying exposure (E) | 32 | Cohen's d | -0.158 (-0.225 to -0.092) | - | - | 94.1 | <0.001 |
|  |  |  | **－** | Bullying exposure (F) | 13 | Cohen's d | -0.122 (-0.173 to -0.071) | - | - | 41.3 | 0.06 |
|  |  |  | **－** | Cyberbullying (E) | 5 | Cohen's d | -0.135 (-0.201 to -0.069) | - | - | 19.7 | 0.29 |
|  |  |  | 🚫 | Cyberbullying (F) | 1 | Cohen's d | NA | - | - | - | - |
|  |  |  | **＋** | Attitudes that discourage bullying (E) | 25 | Cohen's d | 0.195 (0.145 to 0.245) | - | - | 78.4 | <0.001 |
|  |  |  | **＋** | Attitudes that discourage bullying (F) | 14 | Cohen's d | 0.143 (0.083 to 0.202) | - | - | 52.5 | 0.011 |
|  |  |  | **－** | Attitudes that encourage bullying (E) | 15 | Cohen's d | -0.115 (-0.184 to -0.046) | - | - | 85.2 | <0.001 |
|  |  |  | **－** | Attitudes that encourage bullying (F) | 7 | Cohen's d | -0.123 (-0.197 to -0.048) | - | - | 48.6 | 0.07 |
|  |  |  | **－** | Mental health problems (E) | 20 | Cohen's d | -0.205 (-0.277 to -0.133) | -0.014 (-0.097 to 0.069) | -1.410 (-2.063 to -0.757) | 83.7 | <0.001 |
|  |  |  | **－** | Mental health problems (F) | 6 | Cohen's d | -0.202 (-0.347 to -0.056) | - | - | 65.7 | 0.012 |
|  |  |  | **＋** | School climate (E) | 12 | Cohen's d | 0.070 (0.044 to 0.096) | - | - | 0 | 0.7 |
|  |  |  | **＋** | School climate (F) | 5 | Cohen's d | 0.135 (0.037 to 0.233) | - | - | 72 | 0.006 |
| 4 | Guzman- Holst (2022) | Antibullying interventions | **－** | Overall internalizing symptoms | 22 | Hedge's g | 0.06 (0.028 to 0.101) | 0.54 (0.00 to 1.08) | -0.29 (-0.74 to 0.16) | 2.2 | 0.43 |
|  |  |  | **－** | Depression | 12 | Hedge's g | 0.06 (0.014 to 0.107) | - | - | 0 | 0.011 |
|  |  |  | **－** | Anxiety | 14 | Hedge's g | 0.08 (0.011 to 0.158) | - | - | 44 | 0.024 |
| 5 | Hensums (2023) | School‑based anti‑bullying interventions | **－** | Bullying victimization | - | OR | -0.144 (-0.189 to -0.100) | - | - | - | - |
|  |  |  | **－** | Bullying perpetration | - | OR | -0.071 (-0.13 to -0.011) | - | - | - | - |
| 6 | Kamaruddin (2023) | Game-based, skill-building, school-based, and whole-school interventions | 🚫 | Cyberbullying perpetration frequency | 4 | MD | -0.154 (-0.312 to 0.005) | 0.00 (-0.02 to 0.02) | -0.14 (-0.42 to 0.14) | 41 | - |
|  |  |  | 🚫 | Cyberbullying victimization frequency | 2 | MD | -0.258 (-0.614 to 0.099) | -0.03 (-0.06 to 0.00) | -0.26 (-0.48 to -0.04) | 76 | - |
| 7 | Kennedy (2020) | Bullying prevention programs | **－** | Relational bullying victimization | 33 | OR | 0.153 (0.091 to 0.212) | 1.386 (0.408 to 2.364) | -0.713 (-1.496 to 0.070) | 89.65 | <0.001 |
|  |  |  | **－** | Physical bullying victimization | 31 | OR | 0.153 (0.096 to 0.212) | 1.742 (1.023 to 2.461) | -0.817 (-1.276 to -0.359) | 88.46 | <0.001 |
|  |  |  | 🚫 | Verbal bullying victimization | 23 | OR | 0.072 (-0.034 to 0.174) | 0.951 (0.795 to 1.107) | -0.359 (-0.678 to -0.040) | 94.83 | <0.001 |
| 8 | Lan (2022) | Anti-cyberbullying educational programs | **－** | Cyber aggression (S) | 19 | SMD | -0.220 (-0.299 to -0.140) | 0.03 (-0.09 to 0.14) | -0.98 (-1.68 to -0.28) | 81.59 | <0.001 |
|  |  |  | **－** | Cyber aggression (L) | 3 | SMD | -0.162 (-0.397 to 0.074) | 0.00 (-0.08 to 0.08) | -0.37 (-0.70 to -0.05) | 71.79 | 0.029 |
|  |  |  | **－** | Cyber victimization (S) | 16 | SMD | -0.213 (-0.303 to -0.123) | 0.09 (0.03 to 0.14) | -0.58 (-0.79 to -0.36) | 86.454 | <0.001 |
|  |  |  | **－** | Cyber victimization (L) | 3 | SMD | -0.178 (-0.360 to 0.003) | -0.08 (-0.15 to -0.002) | -0.35 (-0.62 to -0.09) | 55.05 | 0.11 |
| 9 | Ng (2022) | Educational interventions | **－** | Bullying victimization frequency | 9 | SMD | -0.18 (-0.26 to -0.10) | -0.03 (-0.38 to 0.33) | -0.46 (-0.69 to -0.24) | 29 | 0.81 |
|  |  |  | 🚫 | Bullying victimization frequency | 2 | RR | -0.014 (-0.036 to 0.008) | 0.99 (0.91 to 1.08) | 0.98 (0.92 to 1.04) | 0 | 0.19 |
|  |  |  | **－** | Bullying perpetration frequency | 9 | SMD | -0.30 (-0.44 to -0.15) | -0.08 (-0.24 to 0.09) | -0.81 (-1.03 to -0.58) | 75 | 0.19 |
|  |  |  | 🚫 | Bullying perpetration frequency | 2 | RR | -0.012 (-0.078 to 0.055) | 1.03 (0.92 to 1.16) | 0.94 (0.85 to 1.03) | 41 | <0.001 |
|  |  |  | **－** | Cyberbullying victimization frequency | 5 | SMD | -0.13 (-0.25 to -0.02) | -0.03 (-0.10 to 0.04) | -0.50 (-0.83 to -0.18) | 73 | 0.005 |
|  |  |  | **－** | Cyberbullying perpetration frequency | 5 | SMD | -0.16 (-0.29 to -0.03) | 0.00 (-0.07 to 0.07) | -0.58 (-0.91 to -0.25) | 80 | <0.001 |
| 10 | Torgal (2023) | School-based cyberbullying prevention programs | 🚫 | Cyber-bystander behavior | 9 | Hedge's g | 0.29 (-0.03 to 0.61) | - | - | 79.76 | - |
| 11 | Wang (2023) | Parent-related interventions | **－** | Cyberbullying victimization frequency (E) | 10 | SMD | -0.17 (-0.24 to -0.10) | -0.03 (-0.10 to 0.04) | -0.54 (-0.84 to -0.23) | 68 | <0.001 |
|  |  |  | **－** | Cyberbullying victimization frequency (F) | 2 | SMD | -0.09 (-0.16 to -0.02) | -0.08(-0.15 to -0.00) | -0.15 (-0.32 to -0.03) | 0 | 0.46 |
|  |  |  | **－** | Cyberbullying perpetration frequency (E) | 9 | SMD | -0.17 (-0.26 to -0.09) | 0.00 (-0.07 to 0.07) | -0.53 (-0.84 to -0.23) | 80 | <0.001 |
|  |  |  | 🚫 | Cyberbullying perpetration frequency (F) | 2 | SMD | -0.10 (-0.31 to 0.12) | 0.00 (-0.07 to 0.07) | -0.22 (-0.40 to -0.05) | 81 | 0.02 |
| 12 | Polanin (2022) | Cyberbullying prevention and intervention  programming | **－** | Cyberbullying perpetration | 44 | Hedge's g | -0.18 (-0.28 to -0.09) | - | - | 79.71 | - |
|  |  |  | **－** | Cyberbullying victimization | 39 | Hedge's g | -0.13 (-0.21 to -0.05) | - | - | 34.9 | - |
|  |  |  | **－** | Bullying perpetration frequency | 22 | Hedge's g | -0.18 (-0.28 to -0.08) | - | - | 55.2 | - |
|  |  |  | **－** | Bullying victimization | 24 | Hedge's g | -0.16 (-0.27 to -0.05) | - | - | 63.21 | - |
| 13 | Barnes (2014) | Cognitive behavioral interventions | 🚫 | Aggressive behaviors | 25 | Cohen's d | -0.14 (-3.23 to 0.62) | - | - | - | >0.05 |
| 14 | Castillo-Eito (2020) | Psychosocial interventions | **－** | Aggression | 95 | Cohen's d | 0.28 (0.17 to 0.39) | - | - | 91.17 | - |
| 15 | Harwood (2017) | Martial arts | **－** | Problematic externalizing behavior | 9 | Cohen's d | 0.65 (0.11 to 1.03) | - | - | 41.99 | >0.05 |
| 16 | Hoogsteder (2015) | Individually oriented cognitive behavioral Treatment | 🚫 | Overall several aggressive problems | 6 | Cohen's d | 1.139 (-0.06 to 3.13) | 3.13 | -0.06 | - | >0.05 |
| 17 | Ouyang (2023) | Physical activity interventions | **－** | Total aggression by others-report | 6 | Hedge's g | -0.28 (-0.55 to - 0.01) | 0.09 (-0.34 to 0.53) | -0.58 (-1.48 to 0.32) | 45.8 | 0.1 |
|  |  |  | 🚫 | Total aggression by self-report | 10 | Hedge's g | -0.32 ( -0.72 to 0.08) | 0.32 (-0.04 to 0.67) | -1.72 (-2.30 to -1.13) | 91.9 | <0.001 |
|  |  |  | 🚫 | Physical aggression | 6 | Hedge's g | -0.45 ( -1.09 to 0.20) | 0.25 (-0.11 to 0.61) | -1.98 (-2.59 to -1.36) | 94.2 | <0.001 |
|  |  |  | 🚫 | Verbal aggression | 5 | Hedge's g | -0.63 ( -2.04 to 0.78) | 0.36 (-0.17 to 0.88) | -3.58 (-4.39 to -2.77) | 97.48 | <0.001 |
|  |  |  | 🚫 | Anger | 6 | Hedge's g | -0.49 ( -1.16 to 0.17) | 0.19 (-0.34 to 0.71) | -2.23 (-2.87 to -1.59) | 95.92 | <0.001 |
|  |  |  | 🚫 | Hostility | 6 | Hedge's g | -0.33 ( -0.69 to 0.04) | 0.06 (-0.30 to 0.41) | -1.24 (-1.79 to -0.69) | 81.55 | <0.001 |
|  |  |  | 🚫 | Indirect aggression | 3 | Hedge's g | -0.30 ( -1.28 to 0.68) | 0.30 (-0.06 to 0.65) | -1.28 (-1.83 to -0.73) | 90.52 | <0.001 |
| 18 | Yang (2023) | Sports intervention | **－** | Overall aggression | 9 | SMD | -0.37 (-0.69 to -0.06) | 0.53 (-0.01 to 1.07) | -3.57 (-4.77 to -2.36) | 88 | - |
|  |  |  | **－** | Hostility | 5 | SMD | -0.29 (-0.41 to -0.17) | 0.13 (-0.40 to 0.66) | -0.36 (-0.64 to -0.09) | 0 | - |
|  |  |  | 🚫 | Anger | 5 | SMD | -0.26 (-0.56 to 0.03) | 0.75 (0.20 to 1.30) | -0.52 (-0.91 to -0.13) | 78 | - |
| 19 | Ye (2021) | Music-based intervention | **－** | Aggressive behavior | 4 | SMD | -0.99 (-1.42 to -0.56) | 0.11 (-1.04 to 1.25) | -3.22 (-4.08 to -2.35) | 94.4 | <0.001 |
|  |  |  | **＋** | Self-control | 3 | SMD | 0.56 (0.19 to 0.93) | 2.31 (1.84 to 2.79) | 0.04 (-0.04 to 0.11) | 96.5 | <0.001 |
|  |  |  | 🚫 | Aggression propensity | 2 | SMD | -0.03 (-0.15 to 0.10) | -0.01 (-0.09 to 0.07) | -0.31 (-0.87 to 0.25) | 6.3 | 0.302 |
|  |  |  | 🚫 | Hyperactivity-impulsivity | 3 | SMD | -0.02 (-0.70 to 0.66) | 1.34 (0.09 to 2.59) | -1.76 (-3.09 to -0.42) | 82.5 | <0.001 |
|  |  |  | 🚫 | Prosocial behavior | 3 | SMD | 0.03 (-0.03 to 0.09) | 0.28 (-0.15 to 0.71) | -0.05 (-0.49 to 0.38) | 2.8 | 0.379 |
| 20 | Jiang (2024) | Community-based programs | **－** | Aggression | 16 | SMD | -0.26 (-0.39 to -0.13) | 0.16 (-0.05 to 0.38) | -0.68 (-1.03 to -0.32) | 58 | 0.002 |
| 21 | Shen (2024) | School-based anti-aggression programs | **－** | Aggressive behaviors | 17 | SMD | -0.35 (-0.53 to -0.18) | 1.30 (0.70 to 1.90) | -1.94 (-2.56 to -1.32) | 89 | <0.001 |
| 22 | Chen (2023) | Digital health interventions (DHIs) | **－** | Overall bullying | 16 | Cohen's d | 0.28 (0.14 to 0.42) | 1.62 (1.38 to 1.86) | 0.02 (-0.28 to 0.33) | 97.81 | <0.001 |
|  |  |  | **－** | Cyberbullying | 13 | Cohen's d | 0.19 (0.11 to 0.27) | - | - | 72.95 | <0.001 |
|  |  |  | **－** | Bullying | 10 | Cohen's d | 0.41 (0.14 to 0.68) | - | - | 97.65 | <0.001 |
|  |  |  | **－** | Combined bullying | 2 | Cohen's d | 0.40 (0.28 to 0.52) | - | - | 0 | 0.796 |
|  |  |  | **－** | Cyberbullying perpetration | 4 | Cohen's d | 0.35 (0.12 to 0.58) | - | - | 74.3 | 0.003 |
|  |  |  | **－** | Bullying perpetration | 5 | Cohen's d | 0.30 (0.18 to 0.41) | - | - | 2.51 | 0.392 |
|  |  |  | **－** | Cyberbullying victimization | 6 | Cohen's d | 0.33 (0.18 to 0.48) | - | - | 64.99 | 0.014 |
|  |  |  | **－** | Bullying victimization | 5 | Cohen's d | 0.37 (0.24 to 0.51) | - | - | 29.71 | 0.223 |
|  |  |  | **－** | Cyberbullying bystander | 2 | Cohen's d | 0.29 (0.09 to 0.49) | - | - | 0 | 0.962 |
|  |  |  | 🚫 | Bullying bystander | 2 | Cohen's d | 0.51 (-0.21 to 1.23) | - | - | 85.24 | 0.009 |
|  |  |  | **－** | Bullying bully–victim dual roles | 2 | Cohen's d | 0.63 (0.05 to 1.21) | - | - | 63.48 | 0.098 |
| 23 | Gaffney (2021) | School‐based programs | **－** | Bullying perpetration | - | OR | 0.149 (0.119 to 0.178) | - | - | 73.716 | <0.001 |
|  |  |  | **－** | Bullying victimization | - | OR | 0.120 (0.096 to 0.149) | - | - | 77.534 | <0.001 |
| 24 | Gaffney (2019) | Cyberbullying intervention and prevention programs | **－** | Cyberbullying perpetration | 18 | OR | 0.116 (0.022 to 0.209) | - | - | 74.81 | <0.001 |
|  |  |  | **－** | Cyberbullying victimization | 18 | OR | 0.113 (0.027 to 0.201) | - | - | 72.44 | <0.001 |
| 25 | Merrel (2008) | School Bullying Intervention Programs | 🚫 | Bullying others | 8 | Cohen's d | 0.04 | - | - | - | - |
|  |  |  | 🚫 | Positive attitude toward bullying | 4 | Cohen's d | 0.15 | - | - | - | - |
|  |  |  | 🚫 | Being bullied | 10 | Cohen's d | 0.27 | - | - | - | - |
|  |  |  | 🚫 | Witnessed bullying | 3 | Cohen's d | 0.35 | - | - | - | - |
|  |  |  | 🚫 | Intervene to stop bullying | 3 | Cohen's d | 0.17 | - | - | - | - |
|  |  |  | 🚫 | Bullies were talked to by adult | 2 | Cohen's d | -0.04 | - | - | - | - |
|  |  |  | 🚫 | Teacher action/response | 3 | Cohen's d | 0.06 | - | - | - | - |
|  |  |  | 🚫 | Ignore/refuse to join bullying | 4 | Cohen's d | 0.06 | - | - | - | - |
|  |  |  | 🚫 | Reported bullying, or likely to | 4 | Cohen's d | 0.07 | - | - | - | - |
|  |  |  | 🚫 | Sympathy for victims | 2 | Cohen's d | -0.10 | - | - | - | - |
|  |  |  | 🚫 | Positive interactions with peers | 4 | Cohen's d | -0.10 | - | - | - | - |
|  |  |  | 🚫 | Staff appropriate responses to bullying | 2 | Cohen's d | 0.30 | - | - | - | - |
|  |  |  | 🚫 | Peer report Identified victims | 2 | Cohen's d | -0.03 | - | - | - | - |
|  |  |  | 🚫 | Peer report Identified aggressors | 3 | Cohen's d | 0.04 | - | - | - | - |
| 26 | Lee (2015) | School-based anti-bullying programs | **－** | Bullying victimization | 13 | Cohen's d | -0.151 (-0.201 to -0.101) | - | - | 69.7 | <0.001 |
| 27 | Fossum (2008) | Psychosocial interventions | **－** | Aggressive behaviors (ES1) | 33 | Cohen's d | 0.62 (0.49 to 0.76) | - | - | - | <0.001 |
|  |  |  | **－** | Aggressive behaviors (ES2) | 32 | Cohen's d | 0.95 (0.77 to 1.14) | - | - | - | <0.001 |
|  |  |  | **－** | Teacher reported aggression (ES1) | 20 | Cohen's d | 0.41 (0.30 to 0.52) | - | - | - | - |
|  |  |  | **－** | Teacher reported aggression (ES2) | 7 | Cohen's d | 0.63 (0.39 to 0.86) | - | - | - | - |
|  |  |  | **－** | Social functioning (ES1) | 13 | Cohen's d | 0.42 (0.27 to 0.57) | - | - | - | - |
|  |  |  | **－** | Social functioning (ES2) | 10 | Cohen's d | 0.49 (0.24 to 0.73) | - | - | - | - |
|  |  |  | **－** | Parental distress (ES1) | 16 | Cohen's d | 0.39 (0.23 to 0.57) | - | - | - | - |
|  |  |  | **－** | Parental distress (ES2) | 17 | Cohen's d | 0.47 (0.36 to 0.59) | - | - | - | - |
| 28 | Robinson (1999) | Cognitive behavior modification | 🚫 | Hyperactivity-Impulsivity and aggression | 23 | Cohen's d | 0.74 (-0.25 to 4.80) | - | - | - | >0.05 |
|  |  |  | 🚫 | Hyperactivity-Impulsivity | 17 | Cohen's d | 0.79 (-0.24 to 4.80) | - | - | - | - |
|  |  |  | 🚫 | Aggression | 12 | Cohen's d | 0.64 (-0.25 to 1.67) | - | - | - | - |
| 29 | Smeets (2015) | Cognitive behavior therapy | 🚫 | Aggressive behaviors | - | Cohen's d | 0.5 (-0.19 to 2.35) | - | - | 76 | <0.001 |
| 30 | Tao (2021) | Mindfulness-based interventions | **－** | Aggression | 10 | Hedge's g | 0.48 (0.33 to 0.63) | - | - | 6.71 | <0.001 |

**Notes:** E: End of intervention; F: Follow-up; S: Short term; L: Long term; ES1: Studies involving an untreated control; ES2: Studies involving either a treated control or no control; n: Specific number of articles of each outcome; 95% CI: 95% Confidence Interval.

**－**(including **－**)indicates that the effect was significantly beneficial.

**－**: The Cohen's d was multiplied by -1 in the forest plot showing in main text.

🚫: The effect was not statistically significant.

* The original values were -0.381 [-0.060, 0.821] and -0.195 [0.061 to 0.329] and were adjusted to 0.381 [-0.060, 0.821] and -0.195 [-0.329 to -0.061] throughout the study, respectively, which was checked with the first author via email.

# Table S8. 16-items of AMSTAR-2

| **Item** | **Question** |
| --- | --- |
| 1 | Did the research questions and inclusion criteria for the review include the components of PICO? |
| 2 | Did the report of the review contain an explicit statement that the review methods were established prior to the conduct of the review and did the report justify any significant deviations from the protocol? |
| 3 | Did the review authors explain their selection of the study designs for inclusion in the review? |
| 4 | Did the review authors use a comprehensive literature search strategy? |
| 5 | Did the review authors perform study selection in duplicate? |
| 6 | Did the review authors perform data extraction in duplicate? |
| 7 | Did the review authors provide a list of excluded studies and justify the exclusions? |
| 8 | Did the review authors describe the included studies in adequate detail? |
| 9 | Did the review authors use a satisfactory technique for assessing the risk of bias (RoB) in individual studies that were included in the review? |
| 10 | Did the review authors report on the sources of funding for the studies included in the review? |
| 11 | If meta-analysis was performed, did the review authors use appropriate methods for statistical combination of results? |
| 12 | If meta-analysis was performed, did the review authors assess the potential impact of RoB in individual studies on the results of the meta-analysis or other evidence synthesis? |
| 13 | Did the review authors account for RoB in primary studies when interpreting/discussing the results of the review? |
| 14 | Did the review authors provide a satisfactory explanation for, and discussion of, any heterogeneity observed in the results of the review? |
| 15 | If they performed quantitative synthesis did the review authors carry out an adequate investigation of publication bias (small study bias) and discuss its likely impact on the results of the review? |
| 16 | Did the review authors report any potential sources of conflict of interest, including any funding they received for conducting the review? |
| **Critical domains*** | |
| Item 2, Item 4, Item 7, Item 9, Item 11, Item 13, Item 15 | |
| **Rating overall confidence in the results of the review** | |
| **High**   - No or one non-critical weakness: the systematic review provides an accurate and comprehensive summary of the results of the available studies that address the question of interest.   **Moderate**   - More than one non-critical weakness: the systematic review has more than one weakness but no critical flaws. It may provide an accurate summary of the results of the available studies that were included in the review.   **Low**   - One critical flaw with or without non-critical weaknesses: the review has a critical flaw and may not provide an accurate and comprehensive summary of the available studies that address the question of interest.   **Critically low**   - More than one critical flaw with or without non-critical weaknesses: the review has more than one critical flaw and should not be relied on to provide an accurate and comprehensive summary of the available studies. | |

**Notes:** Multiple non-critical weaknesses may diminish confidence in the review (45).

# Table S9. Results of AMSTAR-2 quality assessment

| **No.** | **First author (year)** | **1** | **2** | **3** | **4** | **5** | **6** | **7** | **8** | **9** | **10** | **11** | **12** | **13** | **14** | **15** | **16** | **Breakdown by critical items** |
| --- | --- | --- | --- | --- | --- | --- | --- | --- | --- | --- | --- | --- | --- | --- | --- | --- | --- | --- |
| 1 | Jiménez- Barbero (2016) | Y | P | Y | Y | Y | Y | Y | Y | Y | N | Y | Y | Y | Y | Y | N | Moderate |
| 2 | Chen (2021) | Y | P | N | Y | Y | Y | Y | Y | Y | N | Y | Y | Y | Y | Y | Y | Moderate |
| 3 | Fraguas (2021) | Y | P | Y | Y | Y | Y | Y | Y | Y | N | Y | Y | Y | Y | Y | Y | High |
| 4 | Guzman- Holst (2022) | Y | P | N | Y | Y | Y | Y | Y | Y | N | Y | Y | Y | Y | Y | Y | Moderate |
| 5 | Hensums (2023) | Y | P | N | P | N | N | N | P | N | N | Y | Y | Y | Y | Y | Y | Critically low |
| 6 | Kamaruddin (2023) | Y | P | Y | Y | N | N | Y | P | Y | N | Y | Y | Y | Y | N | Y | Low |
| 7 | Kennedy (2020) | Y | P | N | Y | N | N | P | P | Y | N | Y | N | N | Y | N | N | Critically low |
| 8 | Lan (2022) | Y | P | Y | Y | Y | Y | Y | Y | Y | N | Y | Y | Y | Y | Y | Y | High |
| 9 | Ng (2022) | Y | P | Y | Y | Y | Y | Y | Y | Y | N | Y | Y | Y | Y | Y | Y | High |
| 10 | Torgal (2023) | Y | P | Y | Y | N | N | Y | P | N | N | Y | N | N | Y | N | N | Critically low |
| 11 | Wang (2023) | Y | P | N | Y | Y | Y | Y | Y | Y | N | Y | Y | Y | Y | Y | Y | Moderate |
| 12 | Polanin (2022) | Y | P | N | Y | N | N | Y | P | Y | Y | Y | Y | Y | Y | Y | Y | Moderate |
| 13 | Barnes (2014) | Y | P | N | Y | N | N | N | P | N | N | Y | N | N | Y | N | N | Critically low |
| 14 | Castillo-Eito (2020) | Y | P | Y | Y | Y | Y | Y | P | Y | N | Y | Y | Y | Y | N | Y | Low |
| 15 | Harwood (2017) | Y | P | N | P | Y | Y | N | P | Y | N | Y | Y | Y | Y | Y | Y | Low |
| 16 | Hoogsteder (2015) | Y | P | N | Y | N | N | Y | Y | Y | N | Y | Y | Y | Y | Y | N | Moderate |
| 17 | Ouyang (2023) | Y | P | N | P | Y | N | Y | Y | Y | N | Y | Y | Y | Y | Y | Y | Moderate |
| 18 | Yang (2023) | Y | P | N | P | Y | N | Y | Y | Y | N | Y | Y | Y | Y | N | Y | Low |
| 19 | Ye (2021) | Y | P | N | P | Y | Y | Y | Y | Y | N | Y | Y | Y | Y | Y | N | Moderate |
| 20 | Jiang (2024) | Y | P | Y | Y | Y | Y | Y | Y | Y | N | Y | Y | Y | Y | Y | Y | High |
| 21 | Shen (2024) | Y | P | Y | P | Y | Y | Y | Y | Y | N | Y | Y | Y | Y | Y | Y | High |
| 22 | Chen (2023) | Y | P | N | Y | Y | Y | Y | Y | Y | N | Y | Y | Y | Y | Y | Y | Moderate |
| 23 | Gaffney (2021) | Y | P | Y | Y | Y | Y | Y | Y | Y | N | Y | Y | Y | Y | Y | N | Moderate |
| 24 | Gaffney (2019) | Y | P | Y | Y | N | N | Y | Y | N | N | Y | N | N | Y | Y | N | Critically low |
| 25 | Merrel (2008) | Y | P | N | Y | N | N | N | P | N | N | Y | N | N | N | N | N | Critically low |
| 26 | Lee (2015) | Y | P | N | P | Y | N | Y | Y | N | N | Y | N | N | Y | N | N | Critically low |
| 27 | Fossum (2008) | Y | P | Y | N | N | N | P | P | N | N | Y | N | N | N | N | N | Critically low |
| 28 | Robinson (1999) | Y | P | N | P | N | N | N | Y | N | N | Y | N | N | Y | N | N | Critically low |
| 29 | Smeets (2015) | Y | P | Y | Y | N | N | N | Y | Y | N | Y | Y | Y | Y | N | Y | Critically low |
| 30 | Tao (2021) | Y | P | N | Y | Y | Y | Y | Y | Y | N | Y | Y | Y | Y | Y | N | Moderate |

**Notes:** Purple indicates yes, yellow indicates partial yes, pink indicates no; red indicates critical items.

# Table S10. GRADE assessment of the credibility of evidence

| **First author (year)** | **Outcome** | **Risk of bias** | **Inconsistency** | **Indirectness** | **Imprecision** | **Publication bias** | **Other considerations** | **Certainty** |
| --- | --- | --- | --- | --- | --- | --- | --- | --- |
| ***Intervention: Collaborative prevention intervention*** | | | | | | | | |
| **Cyberbullying intervention and prevention programs** | | | | | | | | |
| Kamaruddin (2023) | Cyberbullying perpetration | Not Serious | Not Serious | Not Serious | Serious | Serious |  | Very low |
|  | Cyberbullying victimization | Not Serious | Serious | Not Serious | Serious | Serious |  | Very low |
| Polanin (2022) | Cyberbullying perpetration | Serious | Serious | Not Serious | Not Serious | Not Serious |  | Very low |
|  | Cyberbullying victimization | Serious | Not Serious | Not Serious | Not Serious | Not Serious |  | Very low |
|  | Bullying perpetration | Serious | Serious | Not Serious | Not Serious | Not Serious |  | Very low |
|  | Bullying victimization | Serious | Serious | Not Serious | Not Serious | Not Serious |  | Very low |
| Gaffney (2019) | Cyberbullying perpetration | Serious | Serious | Serious | Serious | Serious |  | Very low |
|  | Cyberbullying victimization | Serious | Serious | Serious | Serious | Serious |  | Very low |
| **Bullying prevention programs** | | | | | | | | |
| Kennedy (2020) | Relational bullying victimization | Serious | Serious | Not Serious | Not Serious | Serious |  | Very low |
|  | Physical bullying victimization | Serious | Serious | Not Serious | Not Serious | Serious |  | Very low |
|  | Verbal bullying victimization | Serious | Serious | Not Serious | Serious | Serious |  | Very low |
| **Educational interventions** | | | | | | | | |
| Ng (2022) | Bullying victimization | Serious | Not Serious | Not Serious | Not Serious | Serious |  | Low |
|  | Bullying perpetration | Serious | Serious | Not Serious | Not Serious | Serious |  | Very low |
|  | Cyberbullying victimization | Serious | Serious | Not Serious | Not Serious | Not Serious |  | Low |
|  | Cyberbullying perpetration | Serious | Serious | Not Serious | Not Serious | Not Serious |  | Low |
| **Martial arts** |  |  |  |  |  |  |  |  |
| Harwood (2017) | Externalizing behavior | Serious | Not Serious | Not Serious | Not Serious | Not Serious | Substantial effect size | Low |
| **Physical activity interventions** | | | | | | | | |
| Ouyang (2023) | Total aggression by others-report | Not Serious | Serious | Not Serious | Not Serious | Not Serious |  | Low |
|  | Total aggression by self-report | Not Serious | Not Serious | Not Serious | Not Serious | Not Serious |  | Moderate |
|  | Physical aggression | Not Serious | Serious | Not Serious | Serious | Not Serious |  | Very low |
|  | Verbal aggression | Not Serious | Serious | Not Serious | Serious | Not Serious |  | Very low |
|  | Anger | Not Serious | Serious | Not Serious | Serious | Not Serious |  | Very low |
|  | Hostility | Not Serious | Serious | Not Serious | Serious | Not Serious |  | Very low |
|  | Indirect aggression | Not Serious | Serious | Not Serious | Serious | Not Serious |  | Very low |
| **Sports intervention** |  |  |  |  |  |  |  |  |
| Yang (2023) | Aggressive behaviors | Not Serious | Serious | Not Serious | Not Serious | Serious |  | Very low |
|  | Hostility | Not Serious | Not Serious | Not Serious | Not Serious | Serious |  | Low |
|  | Anger | Not Serious | Serious | Not Serious | Serious | Serious |  | Very low |
| **Psychosocial interventions** | | | | | | | | |
| Castillo Eito (2020) | Aggressive behaviors | Serious | Serious | Serious | Serious | Serious | Dose-response relation | Very low |
| Fossum (2008) | Aggressive behaviors | Serious | Serious | Not Serious | Not Serious | Serious | Substantial effect size | Very low |
|  | Teacher reported aggression | Serious | Serious | Not Serious | Not Serious | Serious |  | Very low |
|  | Social functioning | Serious | Serious | Not Serious | Not Serious | Serious |  | Very low |
|  | Parental distress | Serious | Serious | Not Serious | Not Serious | Serious |  | Very low |
| **Mindfulness-based interventions** | | | | | | | | |
| Tao (2021) | Aggressive behaviors | Not Serious | Not Serious | Not Serious | Not Serious | Not Serious |  | Moderate |
| ***Intervention: Community-based prevention intervention*** | | | | | | | | |
| **Community-based programs** | | | | | | | | |
| Jiang (2024) | Aggressive behaviors | Not Serious | Serious | Not Serious | Not Serious | Not Serious |  | Low |
| **Digital health interventions** | | | | | | | | |
| Chen (2023) | Overall bullying | Not Serious | Serious | Not Serious | Not Serious | Not Serious |  | Low |
|  | Cyberbullying | Not Serious | Serious | Not Serious | Not Serious | Not Serious | Dose-response relation | Moderate |
|  | Bullying | Not Serious | Serious | Not Serious | Not Serious | Not Serious |  | Low |
|  | Combined bullying | Not Serious | Not Serious | Not Serious | Not Serious | Not Serious |  | Moderate |
|  | Cyberbullying perpetration | Not Serious | Serious | Not Serious | Not Serious | Not Serious |  | Low |
|  | Bullying perpetration | Not Serious | Not Serious | Not Serious | Not Serious | Not Serious |  | Moderate |
|  | Cyberbullying victimization | Not Serious | Serious | Not Serious | Not Serious | Not Serious |  | Low |
|  | Bullying victimization | Not Serious | Not Serious | Not Serious | Not Serious | Not Serious |  | Moderate |
|  | Cyberbullying bystander | Not Serious | Not Serious | Not Serious | Not Serious | Not Serious |  | Moderate |
|  | Bullying bystander | Not Serious | Serious | Not Serious | Serious | Not Serious |  | Low |
|  | Bullying bully-victim dual roles | Not Serious | Serious | Not Serious | Not Serious | Not Serious | Substantial effect size | Moderate |
| ***Intervention: School-based intervention*** | | | | | | | | |
| **School-based anti-bullying interventions** | | | | | | | | |
| Hensums (2023) | Bullying perpetration | Not Serious | Serious | Not Serious | Not Serious | Not Serious | Dose-response relation | Moderate |
|  | Bullying victimization | Not Serious | Serious | Not Serious | Not Serious | Not Serious |  | Low |
| Guzman-Holst (2022) | Internalizing symptoms | Not Serious | Not Serious | Not Serious | Not Serious | Not Serious |  | Low |
|  | Depression symptoms | Not Serious | Not Serious | Not Serious | Not Serious | Not Serious |  | Low |
|  | Anxiety | Not Serious | Not Serious | Not Serious | Not Serious | Not Serious |  | Low |
| Fraguas (2021) | Overall bullying | Not Serious | Serious | Not Serious | Not Serious | Serious |  | Low |
|  | Bullying perpetration | Not Serious | Serious | Not Serious | Not Serious | Serious |  | Low |
|  | Bullying victimization | Not Serious | Serious | Not Serious | Not Serious | Not Serious |  | Moderate |
|  | Cyberbullying | Not Serious | Not Serious | Not Serious | Not Serious | Not Serious |  | High |
|  | Attitudes that discourage bullying | Not Serious | Serious | Not Serious | Not Serious | Serious |  | Low |
|  | Attitudes that encourage bullying | Not Serious | Serious | Not Serious | Not Serious | Not Serious |  | Moderate |
|  | Mental health problems | Not Serious | Serious | Not Serious | Not Serious | Serious |  | Low |
|  | School climate | Not Serious | Not Serious | Not Serious | Not Serious | Serious |  | Moderate |
| Jiménez-Barbero (2016) | Bullying perpetration | Not Serious | Serious | Not Serious | Not Serious | Not Serious |  | Moderate |
|  | Bullying victimization | Not Serious | Serious | Not Serious | Serious | Serious |  | Very low |
|  | Attitudes that encourage bullying | Not Serious | Not Serious | Not Serious | Not Serious | Not Serious |  | High |
|  | Attitudes that discourage bullying | Not Serious | Not Serious | Not Serious | Not Serious | Not Serious |  | High |
|  | School climate | Not Serious | Not Serious | Not Serious | Serious | Serious |  | Low |
| Lee (2015) | Bullying victimization | Serious | Serious | Serious | Serious | Serious |  | Very low |
| **School-based bullying/ cyberbullying interventions** | | | | | | | | |
| Torgal (2023) | Cyberbullying bystander | Serious | Serious | Serious | Serious | Serious |  | Very low |
| Gaffney (2021) | Bullying perpetration | Not Serious | Serious | Not Serious | Not Serious | Not Serious |  | Low |
|  | Bullying victimization | Not Serious | Serious | Not Serious | Not Serious | Not Serious |  | Low |
| Merrel (2008) | Bullying perpetration | Serious | Serious | Not Serious | Serious | Serious |  | Very low |
|  | Attitudes that encourage bullying | Serious | Serious | Not Serious | Serious | Serious |  | Very low |
|  | Bullying victimization | Serious | Serious | Not Serious | Serious | Serious |  | Very low |
|  | Bullying bystander | Serious | Serious | Not Serious | Not Serious | Serious |  | Very low |
|  | Intervene to stop bullying | Serious | Serious | Not Serious | Serious | Serious |  | Very low |
|  | Parent-child communication about bullying | Serious | Serious | Not Serious | Serious | Serious |  | Very low |
|  | Self-reported teacher action/response | Serious | Serious | Not Serious | Not Serious | Serious |  | Very low |
|  | Attitudes that discourage bullying | Serious | Serious | Not Serious | Serious | Serious |  | Very low |
|  | Reported bullying, or likely to | Serious | Serious | Not Serious | Serious | Serious |  | Very low |
|  | Empathy | Serious | Serious | Not Serious | Serious | Serious |  | Very low |
|  | Positive interactions with peers | Serious | Serious | Not Serious | Serious | Serious |  | Very low |
|  | Staff responses to bullying | Serious | Serious | Not Serious | Serious | Serious |  | Very low |
|  | Peer-reported identified victims | Serious | Serious | Not Serious | Serious | Serious |  | Very low |
|  | Peer-reported identified aggressors | Serious | Serious | Not Serious | Serious | Serious |  | Very low |
| **School-based anti-aggression programs** | | | | | | | | |
| Shen (2024) | Aggressive behaviors | Not Serious | Serious | Not Serious | Not Serious | Not Serious |  | Low |
| **Anti-cyberbullying educational programs** | | | | | | | | |
| Lan (2022) | Cyberbullying perpetration | Serious | Serious | Not Serious | Not Serious | Not Serious |  | Very low |
|  | Cyberbullying victimization | Serious | Serious | Not Serious | Not Serious | Not Serious |  | Very low |
| ***Intervention: Family-based intervention*** | | | | | | | | |
| **Parent-related interventions** | | | | | | | | |
| Chen (2021) | Overall bullying | Not Serious | Serious | Not Serious | Not Serious | Not Serious | Substantial effect size | Moderate |
|  | Depression* | Not Serious | Serious | Not Serious | Serious | Not Serious |  | Very low |
|  | Positive parenting | Not Serious | Not Serious | Not Serious | Not Serious | Not Serious |  | Moderate |
|  | Negative parenting* | Not Serious | Not Serious | Not Serious | Not Serious | Not Serious |  | Moderate |
| Wang (2023) | Cyberbullying victimization | Serious | Serious | Not Serious | Not Serious | Serious |  | Very low |
|  | Cyberbullying perpetration | Serious | Serious | Not Serious | Not Serious | Serious |  | Very low |
| ***Intervention: Healthcare intervention*** | | | | | | | | |
| **Music-based intervention** | | | | | | | | |
| Ye (2021) | Aggressive behavior | Not Serious | Serious | Not Serious | Not Serious | Serious |  | Low |
|  | Self-control | Not Serious | Serious | Not Serious | Not Serious | Not Serious | Substantial effect size | Moderate |
|  | Aggression propensity | Not Serious | Not Serious | Not Serious | Serious | Not Serious |  | Low |
|  | Hyperactivity-impulsivity | Not Serious | Serious | Not Serious | Serious | Not Serious |  | Very low |
|  | Prosocial behavior | Not Serious | Not Serious | Not Serious | Serious | Not Serious |  | Low |
| **Cognitive behavioral interventions** | | | | | | | | |
| Smeets (2015) | Aggressive behaviors | Serious | Serious | Not Serious | Serious | Serious |  | Very low |
| Hoogsteder (2015) | Aggressive behaviors | Serious | Serious | Not Serious | Serious | Not Serious |  | Very low |
| Barnes (2014) | Aggressive behaviors | Serious | Serious | Not Serious | Serious | Not Serious |  | Very low |
| Robinson (1999) | Hyperactivity-Impulsivity and aggression | Serious | Serious | Not Serious | Serious | Not Serious | Dose-response relation | Very low |
|  | Hyperactivity-Impulsivity | Serious | Serious | Not Serious | Serious | Not Serious |  | Very low |
|  | Aggressive behaviors | Serious | Serious | Not Serious | Serious | Not Serious |  | Very low |

# Table S11. Classifications of bullying prevention interventions and outcomes

| **No.** | **First author (year)** | **Name of primary prevention intervention** | **Prevention intervention** | **Sub-prevention intervention** | **Name of primary outcome** | **Name of current outcome** | |
| --- | --- | --- | --- | --- | --- | --- | --- |
|  |  |  |  |  |  | **Outcome** | **Sub-outcome** |
| 1 | Jiménez-Barbero (2016) | Anti-bullying school programs | School-based prevention intervention | School-based anti-bullying interventions | Bullying or school violence frequency | Behavior change | Bullying perpetration |
|  |  |  |  |  | Victimization frequency | Behavior change | Bullying victimization |
|  |  |  |  |  | Attitudes favoring bullying or school violence | Attitude change | Attitudes that encourage bullying |
|  |  |  |  |  | Attitudes against bullying or school violence | Attitude change | Attitudes that discourage bullying |
|  |  |  |  |  | School climate | Attitude change | School climate |
| 2 | Chen (2021) | Parenting programs | Family-based prevention intervention | Parent-related bullying/ cyberbullying interventions | Overall bullying | Behavior change | Overall bullying |
|  |  |  |  |  | Children's depression | Mental health | Depression |
|  |  |  |  |  | Positive parenting | Behavior change | Positive parenting |
|  |  |  |  |  | Negative parenting | Behavior change | Negative parenting |
| 3 | Fraguas (2021) | School anti-bullying interventions | School-based prevention intervention | School-based anti-bullying interventions | Overall bullying | Behavior change | Overall bullying |
|  |  |  |  |  | Bullying perpetration | Behavior change | Bullying perpetration |
|  |  |  |  |  | Bullying exposure | Behavior change | Bullying victimization |
|  |  |  |  |  | Cyberbullying (both perpetration and exposure) | Behavior change | Cyberbullying |
|  |  |  |  |  | Attitudes that discourage bullying | Attitude change | Attitudes that discourage bullying |
|  |  |  |  |  | Attitudes that encourage bullying | Attitude change | Attitudes that encourage bullying |
|  |  |  |  |  | Mental health problems | Mental health | Mental health problems |
|  |  |  |  |  | School climate | Attitude change | School climate |
| 4 | Guzman- Holst (2022) | Antibullying interventions | School-based prevention intervention | School-based anti-bullying interventions | Overall internalizing symptoms (Depression and anxiety) | Mental health | Internalizing symptoms |
|  |  |  |  |  | Depression | Mental health | Depression |
|  |  |  |  |  | Anxiety | Mental health | Anxiety |
| 5 | Hensums (2023) | School‑based anti‑bullying interventions | School-based prevention intervention | School-based anti-bullying interventions | Bullying victimization | Behavior change | Bullying victimization |
|  |  |  |  |  | Bullying perpetration | Behavior change | Bullying perpetration |
| 6 | Kamaruddin (2023) | Game based, skill-building, school-based, and whole-school interventions | Collaborative prevention intervention | Cyberbullying intervention and prevention programs | Cyberbullying perpetration frequency | Behavior change | Cyberbullying perpetration |
|  |  |  |  |  | Cyberbullying victimization frequency | Behavior change | Cyberbullying victimization |
| 7 | Kennedy (2020) | Bullying prevention programs | Collaborative prevention intervention | Bullying prevention programs | Relational bullying victimization | Behavior change | Relational bullying victimization |
|  |  |  |  |  | Physical bullying victimization | Behavior change | Physical bullying victimization |
|  |  |  |  |  | Verbal bullying victimization | Behavior change | Verbal bullying victimization |
| 8 | Lan (2022) | Anti-cyberbullying educational programs | School-based prevention intervention | Anti-cyberbullying educational programs | Cyber aggression | Behavior change | Cyberbullying perpetration |
|  |  |  |  |  | Cyber victimization | Behavior change | Cyberbullying victimization |
| 9 | Ng (2022) | Educational interventions | Collaborative prevention intervention | Educational interventions | Bullying victimization frequency | Behavior change | Bullying victimization |
|  |  |  |  |  | Bullying perpetration frequency | Behavior change | Bullying perpetration |
|  |  |  |  |  | Cyberbullying victimization frequency | Behavior change | Cyberbullying victimization |
|  |  |  |  |  | Cyberbullying perpetration frequency | Behavior change | Cyberbullying perpetration |
| 10 | Torgal (2023) | School-based cyberbullying prevention programs | School-based prevention intervention | School-based bullying/ cyberbullying interventions | Cyber-bystander behavior | Behavior change | Cyberbullying bystander |
| 11 | Wang (2023) | Parent-related  interventions | Family-based prevention intervention | Parent-related bullying/ cyberbullying interventions | Cyberbullying victimization frequency | Behavior change | Cyberbullying victimization |
|  |  |  |  |  | Cyberbullying perpetration frequency | Behavior change | Cyberbullying perpetration |
| 12 | Polanin (2022) | Cyberbullying prevention and intervention  programming | Collaborative prevention intervention | Cyberbullying intervention and prevention programs | Cyberbullying perpetration | Behavior change | Cyberbullying perpetration |
|  |  |  |  |  | Cyberbullying victimization | Behavior change | Cyberbullying victimization |
|  |  |  |  |  | Bullying perpetration frequency | Behavior change | Bullying perpetration |
|  |  |  |  |  | Bullying victimization | Behavior change | Bullying victimization |
| 13 | Barnes (2014) | Cognitive behavioral interventions | Healthcare prevention intervention | Cognitive behavioral interventions | Aggressive behaviors | Behavior change | Aggressive behaviors |
| 14 | Castillo-Eito (2020) | Psychosocial interventions | Collaborative prevention intervention | Psychosocial interventions | Aggression | Behavior change | Aggressive behaviors |
| 15 | Harwood (2017) | Martial arts | Collaborative prevention intervention | Martial arts | Problematic externalizing behavior (aggression, anger, and violence) | Behavior change | Externalizing behavior |
| 16 | Hoogsteder (2015) | Individually oriented Cognitive Behavioral Treatment | Healthcare prevention intervention | Cognitive behavioral interventions | Overall several aggressive problems | Behavior change | Aggressive behaviors |
| 17 | Ouyang (2023) | Physical activity interventions | Collaborative prevention intervention | Physical activity interventions | Total aggression by others-report | Behavior change | Total aggression by others-report |
|  |  |  |  |  | Total aggression by self-report | Behavior change | Total aggression by self-report |
|  |  |  |  |  | Physical aggression | Behavior change | Physical aggression |
|  |  |  |  |  | Verbal aggression | Behavior change | Verbal aggression |
|  |  |  |  |  | Anger | Mental health | Anger |
|  |  |  |  |  | Hostility | Mental health | Hostility |
|  |  |  |  |  | Indirect aggression | Behavior change | Indirect aggression |
| 18 | Yang (2023) | Sports intervention | Collaborative prevention intervention | Sports intervention | Overall aggression | Behavior change | Aggressive behaviors |
|  |  |  |  |  | Hostility | Mental health | Hostility |
|  |  |  |  |  | Anger | Mental health | Anger |
| 19 | Ye (2021) | Music-based intervention | Healthcare prevention intervention | Music-based intervention | Aggressive behavior | Behavior change | Aggressive behaviors |
|  |  |  |  |  | Self-control | Behavior change | Self-control |
|  |  |  |  |  | Aggression propensity | Behavior change | Aggression propensity |
|  |  |  |  |  | Hyperactivity-impulsivity | Mental health | Hyperactivity-impulsivity |
|  |  |  |  |  | Prosocial behavior | Behavior change | Prosocial behavior |
| 20 | Jiang (2024) | Community-based programs | Community-based prevention intervention | Community-based programs | Aggression | Behavior change | Aggressive behaviors |
| 21 | Shen (2024) | School-based programs | School-based prevention intervention | School-based anti-aggression programs | Aggressive behaviors | Behavior change | Aggressive behaviors |
| 22 | Chen (2023) | Digital health interventions (DHIs) | Community-based prevention intervention | Digital health interventions | Overall bullying | Behavior change | Overall bullying |
|  |  |  |  |  | Cyberbullying | Behavior change | Cyberbullying |
|  |  |  |  |  | Bullying | Behavior change | Bullying |
|  |  |  |  |  | Combined bullying | Behavior change | Combined bullying |
|  |  |  |  |  | Cyberbullying perpetration | Behavior change | Cyberbullying perpetration |
|  |  |  |  |  | Bullying perpetration | Behavior change | Bullying perpetration |
|  |  |  |  |  | Cyberbullying victimization | Behavior change | Cyberbullying victimization |
|  |  |  |  |  | Bullying victimization | Behavior change | Bullying victimization |
|  |  |  |  |  | Cyberbullying bystander | Behavior change | Cyberbullying bystander |
|  |  |  |  |  | Bullying bystander | Behavior change | Bullying bystander |
|  |  |  |  |  | Bullying bully–victim dual roles | Behavior change | Bullying bully–victim dual roles |
| 23 | Gaffney (2021) | School‐based programs | School-based prevention intervention | School-based bullying/ cyberbullying interventions | Bullying perpetration | Behavior change | Bullying perpetration |
|  |  |  |  |  | Bullying victimization | Behavior change | Bullying victimization |
| 24 | Gaffney (2019) | Cyberbullying intervention and prevention programs | Collaborative prevention intervention | Cyberbullying intervention and prevention programs | Cyberbullying perpetration | Behavior change | Cyberbullying perpetration |
|  |  |  |  |  | Cyberbullying victimization | Behavior change | Cyberbullying victimization |
| 25 | Merrel (2008) | School Bullying Intervention Programs | School-based prevention intervention | School-based bullying/ cyberbullying interventions | Bullying others | Behavior change | Bullying perpetration |
|  |  |  |  |  | Positive attitude toward bullying | Attitude change | Attitudes that encourage bullying |
|  |  |  |  |  | Being bullied | Behavior change | Bullying victimization |
|  |  |  |  |  | Witnessed bullying | Behavior change | Bullying bystander |
|  |  |  |  |  | Intervene to stop bullying | Behavior change | Intervene to stop bullying |
|  |  |  |  |  | Bullies were talked to by adult | Attitude change | Parent-child communication about bullying |
|  |  |  |  |  | Teacher action/response | Behavior change | Self-reported teacher action/response |
|  |  |  |  |  | Ignore/refuse to join bullying | Attitude change | Attitudes that discourage bullying |
|  |  |  |  |  | Reported bullying, or likely to | Behavior change | Reported bullying, or likely to |
|  |  |  |  |  | Sympathy for victims | Mental health | Empathy |
|  |  |  |  |  | Positive interactions with peers | Behavior change | Positive interactions with peers |
|  |  |  |  |  | Staff appropriate responses to bullying | Behavior change | Staff responses to bullying |
|  |  |  |  |  | Identified victims | Behavior change | Peer-reported identified victims |
|  |  |  |  |  | Identified aggressors | Behavior change | Peer-reported identified aggressors |
| 26 | Lee (2015) | school-based anti-bullying programs | School-based prevention intervention | School-based anti-bullying interventions | Bullying victimization | Behavior change | Bullying victimization |
| 27 | Fossum (2008) | Psychosocial interventions | Collaborative prevention intervention | Psychosocial interventions | Aggressive behaviours | Behavior change | Aggressive behaviors |
|  |  |  |  |  | Teacher reported aggression | Behavior change | Teacher reported aggression |
|  |  |  |  |  | Social functioning | Behavior change | Social functioning |
|  |  |  |  |  | Parental distress | Mental health | Parental distress |
| 28 | Robinson (1999) | Cognitive behavior modification | Healthcare prevention intervention | Cognitive behavioral interventions | Hyperactivity-Impulsivity and aggression | Behavior change | Hyperactivity-Impulsivity and aggression |
|  |  |  |  |  | Hyperactivity-Impulsivity | Mental health | Hyperactivity-Impulsivity |
|  |  |  |  |  | Aggression | Behavior change | Aggressive behaviors |
| 29 | Smeets (2015) | Cognitive behavior therapy | Healthcare prevention intervention | Cognitive behavioral interventions | Aggressive behaviors | Behavior change | Aggressive behaviors |
| 30 | Tao (2021) | Mindfulness-based interventions | Collaborative prevention intervention | Mindfulness-based interventions | Aggression | Behavior change | Aggressive behaviors |

# Table S12. The original effect measures without transformation by prevention interventions

| **First author (year)** | **Outcome** | **Number of participants** | **Number of original studies** | **Name of original effect size** | **Original effect size (95% CI)** |
| --- | --- | --- | --- | --- | --- |
| ***Prevention intervention: Collaborative*** | | | | | |
| **Cyberbullying intervention and prevention programs** | | | | | |
| Kamaruddin (2023) | Cyberbullying perpetration | 3273 | 4 | MD | -0.04(-0.10 to 0.03) |
|  | Cyberbullying victimization | 2954 | 2 |  | -0.12 (-0.34 to 0.10) |
| Polanin (2022) | Cyberbullying perpetration | NA | 44 | SMD | -0.220 (-0.299 to -0.140) |
|  | Cyberbullying victimization | NA | 39 |  | -0.162 (-0.397 to 0.074) |
|  | Bullying perpetration | NA | 22 |  | -0.213 (-0.303 to -0.123) |
|  | Bullying victimization | NA | 24 |  | -0.178 (-0.360 to 0.003) |
| Gaffney (2019) | Cyberbullying perpetration | NA | 18 | OR | 1.233 (1.04-1.46) |
|  | Cyberbullying victimization | NA | 18 |  | 1.144 (0.99-1.33) |
| **Bullying prevention programs** | |  |  |  |  |
| Kennedy (2020) | Relational bullying victimization | NA | 33 | OR | 1.32 (1.18 to 1.47) |
|  | Physical bullying victimization | NA | 31 |  | 1.14 (0.94 to 1.37) |
|  | Verbal bullying victimization | NA | 23 |  | 1.32 (1.19 to 1.47) |
| **Educational interventions** | |  |  |  |  |
| Ng (2020) | Bullying victimization | NA | 9 | SMD | -0.18 (-0.26 to -0.10) |
|  | Bullying perpetration | NA | 9 |  | -0.30 (-0.44 to -0.15) |
|  | Cyberbullying victimization | NA | 5 |  | -0.13 (-0.25 to -0.02) |
|  | Cyberbullying perpetration | NA | 5 |  | -0.16 (-0.29 to -0.03) |
| **Martial arts** |  |  |  |  |  |
| Harwood (2017) | Externalizing behavior | 507 | 9 | Cohen's d | 0.65 (0.11 to 1.03) |
| **Physical activity interventions** | |  |  |  |  |
| Ouyang (2023) | Total aggression by others-report | 857 | 6 | Hedges' g | -0.28 (-0.55 to -0.01) |
|  | Total aggression by self-report | 1587 | 10 |  | -0.32 (-0.72 to 0.08) |
|  | Physical aggression | 1019 | 6 |  | -0.45 (-1.09 to 0.20) |
|  | Verbal aggression | 370 | 5 |  | -0.63 (-2.04 to 0.78) |
|  | Anger | 1197 | 6 |  | -0.49 (-1.16 to 0.17) |
|  | Hostility | 1019 | 6 |  | -0.33 (-0.69 to 0.04) |
|  | Indirect aggression | 210 | 3 |  | -0.30 (-1.28 to 0.68) |
| **Sports intervention** |  |  |  |  |  |
| Yang (2023) | Aggressive behaviors | 1632 | 9 | SMD | -0.37 (-0.69 to -0.06) |
|  | Hostility | 1126 | 5 |  | -0.29 (-0.41 to 0.17) |
|  | Anger | 1126 | 5 |  | -0.26 (-0.56 to 0.03) |
| **Psychosocial interventions** | |  |  |  |  |
| Castillo Eito (2020) | Aggressive behaviors | 111151 | 95 | Cohen's d | 0.28 (0.17 to 0.39) |
| Fossum (2008) | Aggressive behaviors | 2512 | 33 | Cohen's d | 0.62 (0.49-0.76) |
|  | Teacher reported aggression | 1593 | 20 |  | 0.41 (0.3-0.52) |
|  | Social functioning | 1017 | 13 |  | 0.42 (0.27-0.57) |
|  | Parental distress | 1209 | 16 |  | 0.39 (0.23-0.57) |
| **Mindfulness-based interventions** | |  |  |  |  |
| Tao (2021) | Aggressive behaviors | 706 | 10 | Hedges' g | 0.48 (0.33-0.63) |
| ***Prevention intervention:Community-based*** | | | | | |
| **Community-based programs** | |  |  |  |  |
| Jiang (2024) | Aggressive behaviors | 2585 | 16 | SMD | -0.26 (-0.39 to -0.13) |
| **Digital health interventions** | |  |  |  |  |
| Chen (2023) | Overall bullying | NA | 16 | Cohen's d | 0.28 (0.14 to 0.42) |
|  | Cyberbullying | NA | 13 |  | 0.19 (0.11 to 0.27) |
|  | Bullying | NA | 10 |  | 0.41 (0.14 to 0.68) |
|  | Combined bullying | NA | 2 |  | 0.40 (0.28 to 0.52) |
|  | Cyberbullying perpetration | NA | 4 |  | 0.35 (0.12 to 0.58) |
|  | Bullying perpetration | NA | 5 |  | 0.30 (0.18 to 0.41) |
|  | Cyberbullying victimization | NA | 6 |  | 0.33 (0.18 to 0.48) |
|  | Bullying victimization | NA | 5 |  | 0.37 (0.24 to 0.51) |
|  | Cyberbullying bystander | NA | 2 |  | 0.29 (0.09 to 0.49) |
|  | Bullying bystander | NA | 2 |  | 0.51 (-0.21 to 1.23) |
|  | Bullying bully-victim dual roles | NA | 2 |  | 0.63 (0.05 to 1.21) |
| ***Prevention intervention: School-based*** | | | | | |
| **School-based anti-bullying interventions** | | | | | |
| Hensums (2023) | Bullying perpetration | NA | NA | OR | 0.77 (0.71 to 0.83) |
|  | Bullying victimization | NA | NA |  | 0.88 (0.79 to 0.98) |
| Guzman-Holst (2022) | Internalizing symptoms | NA | 22 | Hedges' g | 0.06 (0.0284 to 0.1005) |
|  | Depression symptoms | NA | 12 |  | 0.06 (0.014-0.107) |
|  | Anxiety | NA | 14 |  | 0.08 (0.11 to 0.158) |
| Fraguas (2021) | Overall bullying | 92591 | 45 | Cohen's d | -0.150 (-0.191 to -0.109) |
|  | Bullying perpetration | 86190 | 35 |  | -0.111 (-0.146 to -0.077) |
|  | Bullying victimization | 74191 | 32 |  | -0.158 (-0.225 to -0.092) |
|  | Cyberbullying | 5743 | 5 |  | -0.135 (-0.201 to -0.069) |
|  | Attitudes that discourage bullying | 38351 | 25 |  | 0.195 (0.145 to 0.245) |
|  | Attitudes that encourage bullying | 29921 | 15 |  | -0.115 (-0.184 to -0.046) |
|  | Mental health problems | 29192 | 20 |  | -0.205 (-0.277 to -0.133) |
|  | School climate | 23412 | 12 |  | 0.070 (0.044 to 0.096) |
| Jiménez-Barbero (2016) | Bullying perpetration | 30934 | 14 | SMD | -0.12 (-0.17 to -0.06) |
|  | Bullying victimization | 12493 | 8 |  | -0.09 (-0.17 to 0.00) |
|  | Attitudes that encourage bullying | 1336 | 3 |  | -0.18 (-0.30 to -0.06) |
|  | Attitudes that discourage bullying | 11900 | 4 |  | 0.06 (0.03 to 0.10) |
|  | School climate | 6106 | 3 |  | -0.03 (-0.08 to 0.02) |
| Lee (2015) | Bullying victimization | 19619 | 13 | Cohen's d | -0.151 (-0.201 to -0.101) |
| **School-based bullying/ cyberbullying interventions** | | | | | |
| Torgal (2023) | Cyberbullying bystander | 2434 | 9 | Hedges' g | 0.29 (-0.03 to 0.61) |
| Gaffney (2021) | Bullying perpetration | NA | NA | OR | 1.308 (1.239-1.380) |
|  | Bullying victimization | NA | NA |  | 1.244 (1.19-1.31) |
| Merrel (2008) | Bullying perpetration | NA | 8 | Cohen's d | 0.04 |
|  | Attitudes that encourage bullying | NA | 4 |  | 0.15 |
|  | Bullying victimization | NA | 10 |  | 0.27 |
|  | Bullying bystander | NA | 3 |  | 0.35 |
|  | Intervene to stop bullying | NA | 3 |  | 0.17 |
|  | Parent-child communication about bullying | NA | 2 |  | -0.04 |
|  | Self-reported teacher action/response | NA | 3 |  | 0.06 |
|  | Attitudes that discourage bullying | NA | 4 |  | 0.06 |
|  | Reported bullying, or likely to | NA | 4 |  | 0.07 |
|  | Empathy | NA | 2 |  | -0.10 |
|  | Positive interactions with peers | NA | 4 |  | -0.10 |
|  | Staff responses to bullying | NA | 2 |  | 0.30 |
|  | Peer-reported identified victims | NA | 2 |  | -0.03 |
|  | Peer-reported identified aggressors | NA | 3 |  | 0.04 |
| **School-based anti-aggression programs** | | | | | |
| Shen (2024) | Aggressive behaviors | 5124 | 17 | SMD | -0.35 (-0.53 to -0.18) |
| **Anti-cyberbullying educational programs** | | | | | |
| Lan (2022) | Cyberbullying perpetration | NA | 19 | SMD | -0.220 (-0.299 to -0.140) |
|  | Cyberbullying victimization | NA | 16 |  | -0.213 (-0.303 to -0.123) |
| ***Prevention intervention: Family-based*** | | | | | |
| **Parent-related interventions** | |  |  |  |  |
| Chen (2021) | Overall bullying | NA | 16 | Cohen's d | 0.64 (0.239 to 1.041) |
|  | Depression* | NA | 2 |  | -0.381 (-0.060 to 0.821) |
|  | Positive parenting | NA | 2 |  | 0.180 (0.036 to 0.323) |
|  | Negative parenting* | NA | 3 |  | -0.195 (-0.061 to -0.329) |
| Wang (2023) | Cyberbullying victimization | 15143 | 10 | SMD | -0.17 (-0.24 to -0.10) |
|  | Cyberbullying perpetration | 14716 | 9 |  | -0.17 (-0.26 to -0.09) |
| ***Prevention intervention: Healthcare*** | | | | | |
| **Music-based intervention** | |  |  |  |  |
| Ye (2021) | Aggressive behavior | NA | 4 | SMD | -0.05 (-0.19 to 0.09) |
|  | Self-control | NA | 3 |  | 0.56 (0.19 to 0.93) |
|  | Aggression propensity | NA | 2 |  | -0.03 (-0.15 to 0.10) |
|  | Hyperactivity-impulsivity | NA | 3 |  | -0.02 (-0.70 to 0.66) |
|  | Prosocial behavior | NA | 3 |  | 0.03 (-0.03 to 0.09) |
| **Cognitive behavioral interventions** | |  |  |  |  |
| Smeets (2015) | Aggressive behaviors | 2302 | 25 | SMD | 0.50 (-0.19 to 2.35) |
| Hoogsteder (2015) | Aggressive behaviors | 164 | 6 | Cohen's d | 1.139 (-0.06 to 3.13) |
| Barnes (2014) | Aggressive behaviors | 30309 | 25 | Cohen's d | -0.14 (-3.23 to 0.62) |
| Robinson (1999) | Hyperactivity-Impulsivity and aggression | NA | 23 | Cohen's d | 0.74 (-0.25 to 4.8) |
|  | Hyperactivity-Impulsivity | NA | 17 |  | 0.79 (-0.24 to 4.80) |
|  | Aggressive behaviors | NA | 12 |  | 0.64 (-0.25 to 1.67) |

**Notes:** 95% CI: 95% Confidence Interval.

# Table S13. The original effect measures without transformation by outcomes

| **First author (year)** | **Intervention** | **Number of participants** | **Number of original studies** | **Name of original effect size** | **Original effect size (95% CI)** |
| --- | --- | --- | --- | --- | --- |
| ***Outcome: Behavior change*** | | | | | |
| **Aggressive behaviors** |  |  |  |  |  |
| Ye (2021) | Music-based intervention | NA | 4 | SMD | -0.05 (-0.19 to 0.09) |
| Tao (2021) | Mindfulness-based interventions | 706 | 10 | Hedges' g | 0.48 (0.33-0.63) |
| Yang (2023) | Sports intervention | 1632 | 9 | SMD | -0.37 (-0.69 to -0.06) |
| Shen (2024) | School-based anti-aggression programs | 5124 | 17 | SMD | -0.35 (-0.53 to -0.18) |
| Castillo-Eito (2020) | Psychosocial interventions | 111151 | 95 | Cohen's d | 0.28 (0.17 to 0.39) |
| Fossum (2008) | Psychosocial interventions | 2512 | 33 | Cohen's d | 0.62 (0.49-0.76) |
| Jiang (2024) | Community-based programs | 2585 | 16 | SMD | -0.26 (-0.39 to -0.13) |
| Smeets (2015) | Cognitive behavioral interventions | 2302 | 25 | SMD | 0.50 (-0.19 to 2.35) |
| Barnes (2014) | Cognitive behavioral interventions | 30309 | 25 | Cohen's d | -0.14 (-3.23 to 0.62) |
| Hoogsteder (2015) | Cognitive behavioral interventions | 164 | 6 | Cohen's d | 1.139 (-0.06 to 3.13) |
| Robinson (1999) | Cognitive behavioral interventions | NA | 12 | Cohen's d | 0.64 (-0.25 to 1.67) |
| **Overall bullying** |  |  |  |  |  |
| Chen (2021) | Parent-related bullying/ cyberbullying interventions | NA | 16 | Cohen's d | 0.64 (0.239 to 1.041) |
| Chen (2023) | Digital health interventions | NA | 16 | Cohen's d | 0.28 (0.14 to 0.42) |
| Fraguas (2021) | School-based anti-bullying interventions | 92591 | 45 | Cohen's d | -0.150 (-0.191 to -0.109) |
| **Combined bullying** | |  |  |  |  |
| Chen (2023) | Digital health interventions | NA | 2 | Cohen's d | 0.40 (0.28 to 0.52) |
| **Bullying bully–victim dual roles** | |  |  |  |  |
| Chen (2023) | Digital health interventions | NA | 2 | Cohen's d | 0.63 (0.05 to 1.21) |
| **Verbal aggression** |  |  |  |  |  |
| Ouyang (2023) | Physical activity interventions | 370 | 5 | Hedges' g | -0.63 (-2.04 to 0.78) |
| **Bullying victimization** |  |  |  |  |  |
| Chen (2023) | Digital health interventions | NA | 5 | Cohen's d | 0.37 (0.24 to 0.51) |
| Ng (2020) | Educational interventions | NA | 9 | SMD | -0.18 (-0.26 to -0.10) |
| Polanin (2022) | Cyberbullying intervention and prevention programs | NA | 24 | SMD | -0.178 (-0.360 to 0.003) |
| Hensums (2023) | School-based anti-bullying interventions | NA | NA | OR | 0.88 (0.79 to 0.98) |
| Fraguas (2021) | School-based anti-bullying interventions | 74191 | 32 | Cohen's d | -0.158 (-0.225 to -0.092) |
| Jiménez-Barbero (2016) | School-based anti-bullying interventions | 12493 | 8 | SMD | -0.09 (-0.17 to 0.00) |
| Lee (2015) | School-based anti-bullying interventions | 19619 | 13 | Cohen's d | -0.151 (-0.201 to -0.101) |
| Gaffney (2021) | School-based bullying/ cyberbullying interventions | NA | NA | OR | 1.244 (1.19-1.31) |
| Merrel (2009) | School-based bullying/ cyberbullying interventions | NA | 10 | Cohen's d | 0.27 |
| **Physical aggression** |  |  |  |  |  |
| Ouyang (2023) | Physical activity interventions | 1019 | 6 | Hedges' g | -0.45 (-1.09 to 0.20) |
| **Bullying** |  |  |  |  |  |
| Chen (2023) | Digital health interventions | NA | 10 | Cohen's d | 0.41 (0.14 to 0.68) |
| **Cyberbullying perpetration** | |  |  |  |  |
| Chen (2023) | Digital health interventions | NA | 4 | Cohen's d | 0.35 (0.12 to 0.58) |
| Lan (2022) | Anti-cyberbullying educational programs | NA | 19 | SMD | -0.220 (-0.299 to -0.140) |
| Polanin (2022) | Cyberbullying intervention and prevention programs | NA | 44 | SMD | -0.220 (-0.299 to -0.140) |
| Kamaruddin (2023) | Cyberbullying intervention and prevention programs | 3273 | 4 | MD | -0.04(-0.10 to 0.03) |
| Gaffney (2019) | Cyberbullying intervention and prevention programs | NA | 18 | OR | 1.233 (1.04-1.46) |
| Wang (2023) | Parent-related bullying/ cyberbullying interventions | 14716 | 9 | SMD | -0.17 (-0.26 to -0.09) |
| Ng (2020) | Educational interventions | NA | 5 | SMD | -0.16 (-0.29 to -0.03) |
| **Cyberbullying victimization** | |  |  |  |  |
| Chen (2023) | Digital health interventions | NA | 6 | Cohen's d | 0.33 (0.18 to 0.48) |
| Lan (2022) | Anti-cyberbullying educational programs | NA | 16 | SMD | -0.213 (-0.303 to -0.123) |
| Wang (2023) | Parent-related bullying/ cyberbullying interventions | 15143 | 10 | SMD | -0.17 (-0.24 to -0.10) |
| Polanin (2022) | Cyberbullying intervention and prevention programs | NA | 39 | SMD | -0.162 (-0.397 to 0.074) |
| Kamaruddin (2023) | Cyberbullying intervention and prevention programs | 2954 | 2 | MD | -0.12 (-0.34 to 0.10) |
| Gaffney (2019) | Cyberbullying intervention and prevention programs | NA | 18 | OR | 1.144 (0.99-1.33) |
| Ng (2020) | Educational interventions | NA | 5 | SMD | -0.13 (-0.25 to -0.02) |
| **Total aggression by self-report** | |  |  |  |  |
| Ouyang (2023) | Physical activity interventions | 1587 | 10 | Hedges' g | -0.32 (-0.72 to 0.08) |
| **Indirect aggression** |  |  |  |  |  |
| Ouyang (2023) | Physical activity interventions | 210 | 3 | Hedges' g | -0.30 (-1.28 to 0.68) |
| **Total aggression by others-report** | |  |  |  |  |
| Ouyang (2023) | Physical activity interventions | 857 | 6 | Hedges' g | -0.28 (-0.55 to -0.01) |
| **Bullying perpetration** |  |  |  |  |  |
| Chen (2023) | Digital health interventions | NA | 5 | Cohen's d | 0.30 (0.18 to 0.41) |
| Ng (2020) | Educational interventions | NA | 9 | SMD | -0.30 (-0.44 to -0.15) |
| Polanin (2022) | Cyberbullying intervention and prevention programs | NA | 22 | SMD | -0.213 (-0.303 to -0.123) |
| Gaffney (2021) | School-based bullying/ cyberbullying interventions | NA | NA | OR | 1.308 (1.239-1.380) |
| Merrel (2008) | School-based bullying/ cyberbullying interventions | NA | 8 | Cohen's d | 0.04 |
| Hensums (2023) | School-based anti-bullying interventions | NA | NA | OR | 0.77 (0.71 to 0.83) |
| Fraguas (2021) | School-based anti-bullying interventions | 86190 | 35 | Cohen's d | -0.111 (-0.146 to -0.077) |
| Jiménez-Barbero (2016) | School-based anti-bullying interventions | 30934 | 14 | SMD | -0.12 (-0.17 to -0.06) |
| **Cyberbullying bystander** | |  |  |  |  |
| Chen (2023) | Digital health interventions | NA | 2 | Cohen's d | 0.29 (0.09 to 0.49) |
| Torgal (2023) | School-based bullying/ cyberbullying interventions | 2434 | 9 | Hedges' g | 0.29 (-0.03 to 0.61) |
| **Negative parenting** |  |  |  |  |  |
| Chen (2021) | Parent-related bullying/ cyberbullying interventions * | NA | 3 | Cohen's d | -0.195 (-0.061 to -0.329) |
| **Relational bullying victimization** | |  |  |  |  |
| Kennedy (2020) | Bullying prevention programs | NA | 33 | OR | 1.32 (1.18 to 1.47) |
| **Physical bullying victimization** | |  |  |  |  |
| Kennedy (2020) | Bullying prevention programs | NA | 31 | OR | 1.14 (0.94 to 1.37) |
| **Cyberbullying** |  |  |  |  |  |
| Chen (2023) | Digital health interventions | NA | 13 | Cohen's d | 0.19 (0.11 to 0.27) |
| Fraguas (2021) | School-based anti-bullying interventions | 5743 | 5 | Cohen's d | -0.135 (-0.201 to -0.069) |
| **Positive interactions with peers** | |  |  |  |  |
| Merrel (2008) | School-based bullying/ cyberbullying interventions | NA | 4 | Cohen's d | -0.10 |
| **Aggression propensity** |  |  |  |  |  |
| Ye (2021) | Music-based intervention | NA | 2 | SMD | -0.03 (-0.15 to 0.10) |
| **Peer-reported identified victims** | |  |  |  |  |
| Merrel (2008) | School-based bullying/ cyberbullying interventions | NA | 2 | Cohen's d | -0.03 |
| **Hyperactivity-impulsivity and aggression** | |  |  |  |  |
| Robinson (1999) | Cognitive behavioral interventions | NA | 23 | Cohen's d | 0.74 (-0.25 to 4.8) |
| **Bullying bystander** |  |  |  |  |  |
| Chen (2023) | Digital health interventions | NA | 2 | Cohen's d | 0.51 (-0.21 to 1.23) |
| Merrel (2008) | School-based bullying/ cyberbullying interventions | NA | 3 | Cohen's d | 0.35 |
| **Staff responses to bullying** | |  |  |  |  |
| Merrel (2008) | School-based bullying/ cyberbullying interventions | NA | 2 | Cohen's d | 0.30 |
| **Positive parenting** |  |  |  |  |  |
| Chen (2021) | Parent-related bullying/ cyberbullying interventions | NA | 2 | Cohen's d | 0.180 (0.036 to 0.323) |
| **Intervene to stop bullying** | |  |  |  |  |
| Merrel (2008) | School-based bullying/ cyberbullying interventions | NA | 3 | Cohen's d | 0.17 |
| **Verbal bullying victimization** | |  |  |  |  |
| Kennedy (2020) | Bullying prevention programs | NA | 23 | OR | 1.32 (1.19 to 1.47) |
| **Reported bullying, or likely to** | |  |  |  |  |
| Merrel (2008) | School-based bullying/ cyberbullying interventions | NA | 4 | Cohen's d | 0.07 |
| **Self-reported teacher action/response** | |  |  |  |  |
| Merrel (2008) | School-based bullying/ cyberbullying interventions | NA | 3 | Cohen's d | 0.06 |
| **Peer-reported identified aggressors** | |  |  |  |  |
| Merrel (2008) | School-based bullying/ cyberbullying interventions | NA | 3 | Cohen's d | 0.04 |
| **Prosocial behavior** |  |  |  |  |  |
| Ye (2021) | Music-based intervention | NA | 3 | SMD | 0.03 (-0.03 to 0.09) |
| **Externalizing behavior** |  |  |  |  |  |
| Harwood (2017) | Martial arts | 507 | 9 | Cohen's d | 0.65 (0.11 to 1.03) |
| **Self-control** |  |  |  |  |  |
| Ye (2021) | Music-based intervention | NA | 3 | SMD | 0.56 (0.19 to 0.93) |
| **Teacher reported aggression** | |  |  |  |  |
| Fossum (2008) | Psychosocial interventions | 1593 | 20 | Cohen's d | 0.41 (0.3-0.52) |
| **Social functioning** |  |  |  |  |  |
| Fossum (2008) | Psychosocial interventions | 1017 | 13 | Cohen's d | 0.42 (0.27-0.57) |
| ***Outcome: Attitude change*** | | | | | |
| Attitudes that discourage bullying | |  |  |  |  |
| Fraguas (2021) | School-based anti-bullying interventions | 38351 | 25 | Cohen's d | 0.195 (0.145 to 0.245) |
| Jiménez-Barbero (2016) | School-based anti-bullying interventions | 11900 | 4 | SMD | 0.06 (0.03 to 0.10) |
| Merrel (2008) | School-based bullying/ cyberbullying interventions | NA | 4 | Cohen's d | 0.06 |
| **Attitudes that encourage bullying** | |  |  |  |  |
| Merrel (2008) | School-based bullying/ cyberbullying interventions | NA | 4 | Cohen's d | 0.15 |
| Jiménez-Barbero (2016) | School-based anti-bullying interventions | 1336 | 3 | SMD | -0.18 (-0.30 to -0.06) |
| Fraguas (2021) | School-based anti-bullying interventions | 29921 | 15 | Cohen's d | -0.115 (-0.184 to -0.046) |
| **School climate** |  |  |  |  |  |
| Fraguas (2021) | School-based anti-bullying interventions | 23412 | 12 | Cohen's d | 0.070 (0.044 to 0.096) |
| Jiménez-Barbero (2016) | School-based anti-bullying interventions | 6106 | 3 | SMD | -0.03 (-0.08 to 0.02) |
| **Parent-child communication about bullying** | |  |  |  |  |
| Merrel (2008) | School-based bullying/ cyberbullying interventions | NA | 2 | Cohen's d | -0.04 |
| ***Outcome: Mental health*** | | | | | |
| **Anger** |  |  |  |  |  |
| Ouyang (2023) | Physical activity interventions | 1197 | 6 | Hedges' g | -0.49 (-1.16 to 0.17) |
| Yang (2023) | Sports intervention | 1126 | 5 | SMD | -0.26 (-0.56 to 0.03) |
| **Depression or depression symptom** | |  |  |  |  |
| Chen (2021) | Parent-related bullying/ cyberbullying interventions * | NA | 2 | Cohen's d | -0.381 (-0.060 to 0.821) |
| Guzman-Holst (2022) | School-based anti-bullying interventions | NA | 12 | Hedges' g | 0.06 (0.014-0.107) |
| **Hostility** |  |  |  |  |  |
| Ouyang (2023) | Physical activity interventions | 1019 | 6 | Hedges' g | -0.33 (-0.69 to 0.04) |
| Yang (2023) | Sports intervention | 1126 | 5 | SMD | -0.29 (-0.41 to 0.17) |
| **Mental health problems** |  |  |  |  |  |
| Fraguas (2021) | School-based anti-bullying interventions | 29192 | 20 | Cohen's d | -0.205 (-0.277 to -0.133) |
| **Anxiety** |  |  |  |  |  |
| Guzman-Holst (2022) | School-based anti-bullying interventions | NA | 14 | Hedges' g | 0.08 (0.11 to 0.158) |
| **Internalizing symptoms** |  |  |  |  |  |
| Guzman-Holst (2022) | School-based anti-bullying interventions | NA | 22 | Hedges' g | 0.06 (0.0284 to 0.1005) |
| **Hyperactivity-impulsivity** | |  |  |  |  |
| Ye (2021) | Music-based intervention | NA | 3 | SMD | -0.02 (-0.70 to 0.66) |
| Robinson (1999) | Cognitive behavioral interventions | NA | 17 | Cohen's d | 0.79 (-0.24 to 4.80) |
| **Empathy** |  |  |  |  |  |
| Merrel (2008) | School-based bullying/ cyberbullying interventions | NA | 2 | Cohen's d | -0.10 |
| **Parental distress** |  |  |  |  |  |
| Fossum (2008) | Psychosocial interventions | 1209 | 16 | Cohen's d | 0.39 (0.23-0.57) |

**Notes:** 95% CI: 95% Confidence Interval.

# Table S14a. Cohen's d by prevention intervention subgroup (Complete version)

| **First author (year)** | **Outcome** | **n** | **Cohen's d** | **95% CI** | |
| --- | --- | --- | --- | --- | --- |
|  |  |  |  | **Lower** | **Higher** |
| ***Prevention intervention: Collaborative*** | | | | | |
| **Cyberbullying intervention and prevention programs** | | | | | |
| Kamaruddin (2023) | Cyberbullying perpetration | 4 | -0.15 | -0.31 | 0.01 |
|  | Cyberbullying victimization | 2 | -0.26 | -0.61 | 0.10 |
| Polanin (2022) | Cyberbullying perpetration | 44 | -0.18 | -0.28 | -0.09 |
|  | Cyberbullying victimization | 39 | -0.13 | -0.21 | -0.05 |
|  | Bullying perpetration | 22 | -0.18 | -0.28 | -0.08 |
|  | Bullying victimization | 24 | -0.16 | -0.27 | -0.05 |
| Gaffney (2019) | Cyberbullying perpetration | 18 | -0.12 | -0.21 | -0.02 |
|  | Cyberbullying victimization | 18 | -0.11 | -0.20 | -0.03 |
| **Bullying prevention programs** |  |  |  |  |  |
| Kennedy (2020) | Relational bullying victimization | 33 | -0.15 | -0.21 | -0.09 |
|  | Physical bullying victimization | 31 | -0.15 | -0.21 | -0.10 |
|  | Verbal bullying victimization | 23 | 0.07 | -0.03 | 0.17 |
| **Educational interventions** |  |  |  |  |  |
| Ng (2020) | Bullying victimization | 9 | -0.18 | -0.26 | -0.10 |
|  | Bullying perpetration | 9 | -0.30 | -0.44 | -0.15 |
|  | Cyberbullying victimization | 5 | -0.13 | -0.25 | -0.02 |
|  | Cyberbullying perpetration | 5 | -0.16 | -0.29 | -0.03 |
| **Martial arts** |  |  |  |  |  |
| Harwood (2017) | Externalizing behavior | 9 | -0.65 | -1.03 | -0.11 |
| **Physical activity interventions** |  |  |  |  |  |
| Ouyang (2023) | Total aggression by others-report | 6 | -0.28 | -0.55 | -0.01 |
|  | Total aggression by self-report | 10 | -0.32 | -0.72 | 0.08 |
|  | Physical aggression | 6 | -0.45 | -1.09 | 0.20 |
|  | Verbal aggression | 5 | -0.63 | -2.04 | 0.78 |
|  | Anger | 6 | -0.49 | -1.16 | 0.17 |
|  | Hostility | 6 | -0.33 | -0.69 | 0.04 |
|  | Indirect aggression | 3 | -0.3 | -1.28 | 0.68 |
| **Sports intervention** |  |  |  |  |  |
| Yang (2023) | Aggressive behaviors | 9 | -0.37 | -0.69 | -0.06 |
|  | Hostility | 5 | -0.29 | -0.41 | -0.17 |
|  | Anger | 5 | -0.26 | -0.56 | 0.03 |
| **Psychosocial interventions** |  |  |  |  |  |
| Castillo Eito (2020) | Aggressive behaviors | 95 | -0.28 | -0.39 | -0.17 |
| Fossum (2008) | Aggressive behaviors (ES1) | 33 | -0.62 | -0.76 | -0.49 |
|  | Aggressive behaviors (ES2) | 32 | -0.95 | -1.14 | -0.77 |
|  | Teacher reported aggression (ES1) | 20 | -0.41 | -0.52 | -0.30 |
|  | Teacher reported aggression (ES2) | 7 | -0.63 | -0.86 | -0.39 |
|  | Social functioning (ES1) | 13 | -0.42 | -0.57 | -0.27 |
|  | Social functioning (ES2) | 10 | -0.49 | -0.73 | -0.24 |
|  | Parental distress (ES1) | 16 | -0.39 | -0.57 | -0.23 |
|  | Parental distress (ES2) | 17 | -0.47 | -0.59 | -0.36 |
| **Mindfulness-based interventions** |  |  |  |  |  |
| Tao (2021) | Aggressive behaviors | 10 | -0.48 | -0.63 | -0.33 |
| ***Prevention intervention: Community-based*** | | | | | |
| **Community-based programs** |  |  |  |  |  |
| Jiang (2024) | Aggressive behaviors | 16 | -0.26 | -0.39 | -0.13 |
| **Digital health interventions** |  |  |  |  |  |
| Chen (2023) | Overall bullying | 16 | -0.28 | -0.42 | -0.14 |
|  | Cyberbullying | 13 | -0.19 | -0.27 | -0.11 |
|  | Bullying | 10 | -0.41 | -0.68 | -0.14 |
|  | Combined bullying | 2 | -0.4 | -0.52 | -0.28 |
|  | Cyberbullying perpetration | 4 | -0.35 | -0.58 | -0.12 |
|  | Bullying perpetration | 5 | -0.30 | -0.41 | -0.18 |
|  | Cyberbullying victimization | 6 | -0.33 | -0.48 | -0.18 |
|  | Bullying victimization | 5 | -0.37 | -0.51 | -0.24 |
|  | Cyberbullying bystander | 2 | -0.29 | -0.49 | -0.09 |
|  | Bullying bystander | 2 | 0.51 | -0.21 | 1.23 |
|  | Bullying bully-victim dual roles | 2 | -0.63 | -1.21 | -0.05 |
| ***Prevention intervention: School-based*** | | | | | |
| **School-based anti-bullying interventions** | | | | | |
| Hensums (2023) | Bullying perpetration | NA | -0.07 | -0.13 | -0.01 |
|  | Bullying victimization | NA | -0.14 | -0.19 | -0.10 |
| Guzman-Holst (2022) | Internalizing symptoms | 22 | -0.06 | -0.10 | -0.03 |
|  | Depression symptoms | 12 | -0.06 | -0.11 | -0.01 |
|  | Anxiety | 14 | -0.08 | -0.16 | -0.01 |
| Fraguas (2021) | Overall bullying (E) | 45 | -0.15 | -0.19 | -0.11 |
|  | Overall bullying (F) | 21 | -0.17 | -0.24 | -0.10 |
|  | Bullying perpetration (E) | 35 | -0.11 | -0.15 | -0.08 |
|  | Bullying perpetration (F) | 17 | -0.18 | -0.28 | -0.07 |
|  | Bullying victimization (E) | 32 | -0.16 | -0.23 | -0.09 |
|  | Bullying victimization (F) | 13 | -0.12 | -0.17 | -0.07 |
|  | Cyberbullying | 5 | -0.14 | -0.20 | -0.07 |
|  | Attitudes that discourage bullying (E) | 25 | 0.20 | 0.15 | 0.25 |
|  | Attitudes that discourage bullying (F) | 14 | 0.14 | 0.08 | 0.20 |
|  | Attitudes that encourage bullying (E) | 15 | -0.12 | -0.18 | -0.05 |
|  | Attitudes that encourage bullying (F) | 7 | -0.12 | -0.20 | -0.05 |
|  | Mental health problems (E) | 20 | -0.21 | -0.28 | -0.13 |
|  | Mental health problems (F) | 6 | -0.20 | -0.35 | -0.06 |
|  | School climate (E) | 12 | 0.07 | 0.04 | 0.10 |
|  | School climate (F) | 5 | 0.14 | 0.04 | 0.23 |
| Jiménez-Barbero (2016) | Bullying perpetration | 14 | -0.12 | -0.17 | -0.06 |
|  | Bullying victimization | 8 | -0.09 | -0.18 | 0.01 |
|  | Attitudes that encourage bullying | 3 | -0.18 | -0.30 | -0.06 |
|  | Attitudes that discourage bullying | 4 | 0.06 | 0.03 | 0.10 |
|  | School climate | 3 | -0.03 | -0.08 | 0.02 |
| Lee (2015) | Bullying victimization | 13 | -0.15 | -0.20 | -0.10 |
| **School-based bullying/ cyberbullying interventions** | | | | | |
| Torgal (2023) | Cyberbullying bystander | 9 | 0.29 | -0.03 | 0.61 |
| Gaffney (2021) | Bullying perpetration | NA | -0.15 | -0.18 | -0.12 |
|  | Bullying victimization | NA | -0.12 | -0.15 | -0.10 |
| Merrel (2008) | Bullying perpetration | 8 | 0.04 | NA | NA |
|  | Attitudes that encourage bullying | 4 | 0.15 | NA | NA |
|  | Bullying victimization | 10 | 0.27 | NA | NA |
|  | Bullying bystander | 3 | -0.35 | NA | NA |
|  | Intervene to stop bullying | 3 | 0.17 | NA | NA |
|  | Parent-child communication about bullying | 2 | -0.04 | NA | NA |
|  | Self-reported teacher action/response | 3 | 0.06 | NA | NA |
|  | Attitudes that discourage bullying | 4 | 0.06 | NA | NA |
|  | Reported bullying, or likely to | 4 | 0.07 | NA | NA |
|  | Empathy | 2 | -0.1 | NA | NA |
|  | Positive interactions with peers | 4 | -0.1 | NA | NA |
|  | Staff responses to bullying | 2 | 0.3 | NA | NA |
|  | Peer-reported identified victims | 2 | -0.03 | NA | NA |
|  | Peer-reported identified aggressors | 3 | 0.04 | NA | NA |
| **School-based anti-aggression programs** | | | | | |
| Shen (2024) | Aggressive behaviors | 17 | -0.35 | -0.53 | -0.18 |
| **Anti-cyberbullying educational programs** | | | | | |
| Lan (2022) | Cyberbullying perpetration (S) | 19 | -0.22 | -0.30 | -0.14 |
|  | Cyberbullying perpetration (L) | 3 | -0.16 | -0.40 | 0.07 |
|  | Cyberbullying victimization (S) | 16 | -0.21 | -0.30 | -0.12 |
|  | Cyberbullying victimization (L) | 3 | -0.18 | -0.36 | 0.003 |
| ***Prevention intervention: Family-based*** | | | | | |
| **Parent-related interventions** |  |  |  |  |  |
| Chen (2021) | Overall bullying | 16 | -0.64 | -1.04 | -0.24 |
|  | Depression* | 2 | 0.38 | -0.06 | 0.82 |
|  | Positive parenting | 2 | 0.18 | 0.04 | 0.32 |
|  | Negative parenting* | 3 | -0.20 | -0.33 | -0.06 |
| Wang (2023) | Cyberbullying victimization (E) | 10 | -0.17 | -0.24 | -0.10 |
|  | Cyberbullying victimization (F) | 2 | -0.09 | -0.16 | -0.02 |
|  | Cyberbullying perpetration (E) | 9 | -0.17 | -0.26 | -0.09 |
|  | Cyberbullying perpetration (F) | 2 | -0.10 | -0.31 | 0.12 |
| ***Prevention intervention: Healthcare*** | | | | | |
| **Music-based intervention** |  |  |  |  |  |
| Ye (2021) | Aggressive behavior | 4 | -0.99 | -1.42 | -0.56 |
|  | Self-control | 3 | 0.56 | 0.19 | 0.93 |
|  | Aggression propensity | 2 | -0.03 | -0.15 | 0.10 |
|  | Hyperactivity-impulsivity | 3 | -0.02 | -0.70 | 0.66 |
|  | Prosocial behavior | 3 | 0.03 | -0.03 | 0.09 |
| **Cognitive behavioral interventions** |  |  |  |  |  |
| Smeets (2015) | Aggressive behaviors | 25 | 0.50 | -0.19 | 2.35 |
| Hoogsteder (2015) | Aggressive behaviors | 6 | 1.139 | -0.06 | 3.13 |
| Barnes (2014) | Aggressive behaviors | 25 | -0.14 | -3.23 | 0.62 |
| Robinson (1999) | Hyperactivity-Impulsivity and aggression | 23 | 0.74 | -0.25 | 4.80 |
|  | Hyperactivity-Impulsivity | 17 | 0.79 | -0.24 | 4.80 |
|  | Aggressive behaviors | 12 | 0.64 | -0.25 | 1.67 |

**Notes:** To ensure consistency, the effect sizes of the following studies were multiplied by -1, where the prevention intervention was verified as preventive and beneficial, and the original effect size values were reported as greater than 0 (54, 55): Chen (2021) (20), Guzman- Holst (2022) (61), Kennedy (2020) (62), Castillo-Eito (2020) (28), Harwood (2017) (14), Chen (2023) (63), Gaffney (2021) (40), Gaffney (2019) (64), Fossum (2008) (27), and Tao (2021) (65).

E: End of intervention; F: Follow-up; S: Short term; L: Long term; ES1: Studies involving an untreated control; ES2: Studies involving either a treated control or no control; n: Specific number of articles of each outcome; 95% CI: 95% Confidence Interval.

* The original values were -0.381 [-0.060, 0.821] and -0.195 [0.061 to 0.329] and were adjusted to 0.381 [-0.060, 0.821] and -0.195 [-0.329 to -0.061] throughout the study, respectively, which was checked with the first author via email.

# Table S14b. Cohen's d by prevention intervention subgroup (Simplified version, consistent with forest plot)

| **First author (year)** | **Outcome** | **n** | **Cohen's d** | **95% CI** | |
| --- | --- | --- | --- | --- | --- |
|  |  |  |  | **Lower** | **Higher** |
| ***Intervention: Collaborative prevention intervention*** | | | | | |
| **Cyberbullying intervention and prevention programs** | | | | | |
| Kamaruddin (2023) | Cyberbullying perpetration | 4 | -0.15 | -0.31 | 0.01 |
|  | Cyberbullying victimization | 2 | -0.26 | -0.61 | 0.10 |
| Polanin (2022) | Cyberbullying perpetration | 44 | -0.18 | -0.28 | -0.09 |
|  | Cyberbullying victimization | 39 | -0.13 | -0.21 | -0.05 |
|  | Bullying perpetration | 22 | -0.18 | -0.28 | -0.08 |
|  | Bullying victimization | 24 | -0.16 | -0.27 | -0.05 |
| Gaffney (2019) | Cyberbullying perpetration | 18 | -0.12 | -0.21 | -0.02 |
|  | Cyberbullying victimization | 18 | -0.11 | -0.20 | -0.03 |
| **Bullying prevention programs** |  |  |  |  |  |
| Kennedy (2020) | Relational bullying victimization | 33 | -0.15 | -0.21 | -0.09 |
|  | Physical bullying victimization | 31 | -0.15 | -0.21 | -0.10 |
|  | Verbal bullying victimization | 23 | 0.07 | -0.03 | 0.17 |
| **Educational interventions** |  |  |  |  |  |
| Ng (2020) | Bullying victimization | 9 | -0.18 | -0.26 | -0.10 |
|  | Bullying perpetration | 9 | -0.30 | -0.44 | -0.15 |
|  | Cyberbullying victimization | 5 | -0.13 | -0.25 | -0.02 |
|  | Cyberbullying perpetration | 5 | -0.16 | -0.29 | -0.03 |
| **Martial arts** |  |  |  |  |  |
| Harwood (2017) | Externalizing behavior | 9 | -0.65 | -1.03 | -0.11 |
| **Physical activity interventions** |  |  |  |  |  |
| Ouyang (2023) | Total aggression by others-report | 6 | -0.28 | -0.55 | -0.01 |
|  | Total aggression by self-report | 10 | -0.32 | -0.72 | 0.08 |
|  | Physical aggression | 6 | -0.45 | -1.09 | 0.20 |
|  | Verbal aggression | 5 | -0.63 | -2.04 | 0.78 |
|  | Anger | 6 | -0.49 | -1.16 | 0.17 |
|  | Hostility | 6 | -0.33 | -0.69 | 0.04 |
|  | Indirect aggression | 3 | -0.30 | -1.28 | 0.68 |
| **Sports intervention** |  |  |  |  |  |
| Yang (2023) | Aggressive behaviors | 9 | -0.37 | -0.69 | -0.06 |
|  | Hostility | 5 | -0.29 | -0.41 | -0.17 |
|  | Anger | 5 | -0.26 | -0.56 | 0.03 |
| **Psychosocial interventions** |  |  |  |  |  |
| Castillo Eito (2020) | Aggressive behaviors | 95 | -0.28 | -0.39 | -0.17 |
| Fossum (2008) | Aggressive behaviors | 33 | -0.62 | -0.76 | -0.49 |
|  | Teacher reported aggression | 20 | -0.41 | -0.52 | -0.30 |
|  | Social functioning | 13 | -0.42 | -0.57 | -0.27 |
|  | Parental distress | 16 | -0.39 | -0.57 | -0.23 |
| **Mindfulness-based interventions** |  |  |  |  |  |
| Tao (2021) | Aggressive behaviors | 10 | -0.48 | -0.63 | -0.33 |
| ***Intervention: Community-based prevention intervention*** | | | | | |
| **Community-based programs** |  |  |  |  |  |
| Jiang (2024) | Aggressive behaviors | 16 | -0.26 | -0.39 | -0.13 |
| **Digital health interventions** |  |  |  |  |  |
| Chen (2023) | Overall bullying | 16 | -0.28 | -0.42 | -0.14 |
|  | Cyberbullying | 13 | -0.19 | -0.27 | -0.11 |
|  | Bullying | 10 | -0.41 | -0.68 | -0.14 |
|  | Combined bullying | 2 | -0.40 | -0.52 | -0.28 |
|  | Cyberbullying perpetration | 4 | -0.35 | -0.58 | -0.12 |
|  | Bullying perpetration | 5 | -0.30 | -0.41 | -0.18 |
|  | Cyberbullying victimization | 6 | -0.33 | -0.48 | -0.18 |
|  | Bullying victimization | 5 | -0.37 | -0.51 | -0.24 |
|  | Cyberbullying bystander | 2 | -0.29 | -0.49 | -0.09 |
|  | Bullying bystander | 2 | 0.51 | -0.21 | 1.23 |
|  | Bullying bully-victim dual roles | 2 | -0.63 | -1.21 | -0.05 |
| ***Intervention: School-based intervention*** | | | | | |
| **School-based anti-bullying interventions** | | | | | |
| Hensums (2023) | Bullying perpetration | NA | -0.07 | -0.13 | -0.01 |
|  | Bullying victimization | NA | -0.14 | -0.19 | -0.10 |
| Guzman-Holst (2022) | Internalizing symptoms | 22 | -0.06 | -0.10 | -0.03 |
|  | Depression symptoms | 12 | -0.06 | -0.11 | -0.01 |
|  | Anxiety | 14 | -0.08 | -0.16 | -0.01 |
| Fraguas (2021) | Overall bullying | 45 | -0.15 | -0.19 | -0.11 |
|  | Bullying perpetration | 35 | -0.11 | -0.15 | -0.08 |
|  | Bullying victimization | 32 | -0.16 | -0.23 | -0.09 |
|  | Cyberbullying | 5 | -0.14 | -0.20 | -0.07 |
|  | Attitudes that discourage bullying | 25 | 0.20 | 0.15 | 0.25 |
|  | Attitudes that encourage bullying | 15 | -0.12 | -0.18 | -0.05 |
|  | Mental health problems | 20 | -0.21 | -0.28 | -0.13 |
|  | School climate | 12 | 0.07 | 0.04 | 0.10 |
| Jiménez-Barbero (2016) | Bullying perpetration | 14 | -0.12 | -0.17 | -0.06 |
|  | Bullying victimization | 8 | -0.09 | -0.18 | 0.01 |
|  | Attitudes that encourage bullying | 3 | -0.18 | -0.30 | -0.06 |
|  | Attitudes that discourage bullying | 4 | 0.06 | 0.03 | 0.10 |
|  | School climate | 3 | -0.03 | -0.08 | 0.02 |
| Lee (2015) | Bullying victimization | 13 | -0.15 | -0.20 | -0.10 |
| **School-based bullying/ cyberbullying interventions** | | | | | |
| Torgal (2023) | Cyberbullying bystander | 9 | 0.29 | -0.03 | 0.61 |
| Gaffney (2021) | Bullying perpetration | NA | -0.15 | -0.18 | -0.12 |
|  | Bullying victimization | NA | -0.12 | -0.15 | -0.10 |
| Merrel (2008) | Bullying perpetration | 8 | 0.04 | NA | NA |
|  | Attitudes that encourage bullying | 4 | 0.15 | NA | NA |
|  | Bullying victimization | 10 | 0.27 | NA | NA |
|  | Bullying bystander | 3 | -0.35 | NA | NA |
|  | Intervene to stop bullying | 3 | 0.17 | NA | NA |
|  | Parent-child communication about bullying | 2 | -0.04 | NA | NA |
|  | Self-reported teacher action/response | 3 | 0.06 | NA | NA |
|  | Attitudes that discourage bullying | 4 | 0.06 | NA | NA |
|  | Reported bullying, or likely to | 4 | 0.07 | NA | NA |
|  | Empathy | 2 | -0.10 | NA | NA |
|  | Positive interactions with peers | 4 | -0.10 | NA | NA |
|  | Staff responses to bullying | 2 | 0.30 | NA | NA |
|  | Peer-reported identified victims | 2 | -0.03 | NA | NA |
|  | Peer-reported identified aggressors | 3 | 0.04 | NA | NA |
| **School-based anti-aggression programs** | | | | | |
| Shen (2024) | Aggressive behaviors | 17 | -0.35 | -0.53 | -0.18 |
| **Anti-cyberbullying educational programs** | | | | | |
| Lan (2022) | Cyberbullying perpetration | 19 | -0.22 | -0.30 | -0.14 |
|  | Cyberbullying victimization | 16 | -0.21 | -0.30 | -0.12 |
| ***Intervention: Family-based intervention*** | | | | | |
| **Parent-related interventions** |  |  |  |  |  |
| Chen (2021) | Overall bullying | 16 | -0.64 | -1.04 | -0.24 |
|  | Depression* | 2 | 0.38 | -0.06 | 0.82 |
|  | Positive parenting | 2 | 0.18 | 0.04 | 0.32 |
|  | Negative parenting* | 3 | -0.20 | -0.33 | -0.06 |
| Wang (2023) | Cyberbullying victimization | 10 | -0.17 | -0.24 | -0.10 |
|  | Cyberbullying perpetration | 9 | -0.17 | -0.26 | -0.09 |
| ***Intervention: Healthcare intervention*** | | | | | |
| **Music-based intervention** |  |  |  |  |  |
| Ye (2021) | Aggressive behavior | 4 | -0.99 | -1.42 | -0.56 |
|  | Self-control | 3 | 0.56 | 0.19 | 0.93 |
|  | Aggression propensity | 2 | -0.03 | -0.15 | 0.10 |
|  | Hyperactivity-impulsivity | 3 | -0.02 | -0.70 | 0.66 |
|  | Prosocial behavior | 3 | 0.03 | -0.03 | 0.09 |
| **Cognitive behavioral interventions** |  |  |  |  |  |
| Smeets (2015) | Aggressive behaviors | 25 | 0.50 | -0.19 | 2.35 |
| Hoogsteder (2015) | Aggressive behaviors | 6 | 1.14 | -0.06 | 3.13 |
| Barnes (2014) | Aggressive behaviors | 25 | -0.14 | -3.23 | 0.62 |
| Robinson (1999) | Hyperactivity-Impulsivity and aggression | 23 | 0.74 | -0.25 | 4.80 |
|  | Hyperactivity-Impulsivity | 17 | 0.79 | -0.24 | 4.80 |
|  | Aggressive behaviors | 12 | 0.64 | -0.25 | 1.67 |

**Notes:** n: Specific number of articles of each outcome; 95% CI: 95% Confidence Interval.

* The original values were -0.381 [-0.060, 0.821] and -0.195 [0.061 to 0.329] and were adjusted to 0.381 [-0.060, 0.821] and -0.195 [-0.329 to -0.061] throughout the study, respectively, which was checked with the first author via email.

To ensure consistency, the effect sizes of the following studies were multiplied by -1, where the prevention intervention was verified as preventive and beneficial, and the original effect size values were reported as greater than 0 (54, 55): Chen (2021) (20), Guzman- Holst (2022) (61), Kennedy (2020) (62), Castillo-Eito (2020) (28), Harwood (2017) (14), Chen (2023) (63), Gaffney (2021) (40), Gaffney (2019) (64), Fossum (2008) (27), and Tao (2021) (65).

# Table S15a. Cohen's d by outcome subgroup (Complete version)

| **First author (year)** | **Intervention** | **n** | **Cohen's d** | **95% CI** | |
| --- | --- | --- | --- | --- | --- |
|  |  |  |  | **Lower** | **Higher** |
| ***Outcome: Behavior change*** | | | | | |
| **Aggressive behaviors** |  |  |  |  |  |
| Ye (2021) | Music-based intervention | 4 | -0.99 | -1.42 | -0.56 |
| Tao (2021) | Mindfulness-based interventions | 10 | -0.48 | -0.63 | -0.33 |
| Yang (2023) | Sports intervention | 9 | -0.37 | -0.69 | -0.06 |
| Shen (2024) | School-based anti-aggression programs | 17 | -0.35 | -0.53 | -0.18 |
| Castillo-Eito (2020) | Psychosocial interventions | 95 | -0.28 | -0.39 | -0.17 |
| Fossum (2008) (ES1) | Psychosocial interventions | 33 | -0.62 | -0.76 | -0.49 |
| Fossum (2008) (ES2) | Psychosocial interventions | 32 | -0.95 | -1.14 | -0.77 |
| Jiang (2024) | Community-based programs | 16 | -0.26 | -0.39 | -0.13 |
| Smeets (2015) | Cognitive behavioral interventions | 25 | 0.50 | -0.19 | 2.35 |
| Barnes (2014) | Cognitive behavioral interventions | 25 | -0.14 | -3.23 | 0.62 |
| Hoogsteder (2015) | Cognitive behavioral interventions | 6 | 1.139 | -0.06 | 3.13 |
| Robinson (1999) | Cognitive behavioral interventions | 12 | 0.64 | -0.25 | 1.67 |
| **Overall bullying** |  |  |  |  |  |
| Chen (2021) | Parent-related bullying/ cyberbullying interventions | 16 | -0.64 | -1.04 | -0.24 |
| Chen (2023) | Digital health interventions | 16 | -0.28 | -0.42 | -0.14 |
| Fraguas (2021) (E) | School-based anti-bullying interventions | 45 | -0.15 | -0.19 | -0.11 |
| Fraguas (2021) (F) | School-based anti-bullying interventions | 21 | -0.17 | -0.24 | -0.10 |
| **Combined bullying** |  |  |  |  |  |
| Chen (2023) | Digital health interventions | 2 | -0.4 | -0.52 | -0.28 |
| **Bullying bully–victim dual roles** | |  |  |  |  |
| Chen (2023) | Digital health interventions | 2 | -0.63 | -1.21 | -0.05 |
| **Combined bullying** |  |  |  |  |  |
| Chen (2023) | Digital health interventions | 2 | -0.4 | -0.52 | -0.28 |
| **Verbal aggression** |  |  |  |  |  |
| Ouyang (2023) | Physical activity interventions | 5 | -0.63 | -2.04 | 0.78 |
| **Bullying victimization** |  |  |  |  |  |
| Chen (2023) | Digital health interventions | 5 | -0.37 | -0.51 | -0.24 |
| Ng (2020) | Educational interventions | 9 | -0.18 | -0.26 | -0.10 |
| Polanin (2022) | Cyberbullying intervention and prevention programs | 24 | -0.16 | -0.27 | -0.05 |
| Hensums (2023) | School-based anti-bullying interventions | NA | -0.14 | -0.19 | -0.10 |
| Fraguas (2021) (E) | School-based anti-bullying interventions | 32 | -0.16 | -0.23 | -0.09 |
| Fraguas (2021) (F) | School-based anti-bullying interventions | 13 | -0.12 | -0.17 | -0.07 |
| Jiménez-Barbero (2016) | School-based anti-bullying interventions | 8 | -0.09 | -0.18 | 0.01 |
| Lee (2015) | School-based anti-bullying interventions | 13 | -0.15 | -0.20 | -0.10 |
| Gaffney (2021) | School-based bullying/ cyberbullying interventions | NA | -0.12 | -0.10 | -0.15 |
| Merrel (2009) | School-based bullying/ cyberbullying interventions | 10 | 0.27 | NA | NA |
| **Physical aggression** |  |  |  |  |  |
| Ouyang (2023) | Physical activity interventions | 6 | -0.45 | -1.09 | 0.20 |
| **Bullying** |  |  |  |  |  |
| Chen (2023) | Digital health interventions | 10 | -0.41 | -0.68 | -0.14 |
| **Cyberbullying perpetration** |  |  |  |  |  |
| Chen (2023) | Digital health interventions | 4 | -0.35 | -0.58 | -0.12 |
| Lan (2022) (S) | Anti-cyberbullying educational programs | 19 | -0.22 | -0.30 | -0.14 |
| Lan (2022) (L) | Anti-cyberbullying educational programs | 3 | -0.16 | -0.40 | 0.07 |
| Polanin (2022) | Cyberbullying intervention and prevention programs | 44 | -0.18 | -0.28 | -0.09 |
| Kamaruddin (2023) | Cyberbullying intervention and prevention programs | 4 | -0.15 | -0.31 | 0.01 |
| Gaffney (2019) | Cyberbullying intervention and prevention programs | 18 | -0.12 | -0.21 | -0.02 |
| Wang (2023) (E) | Parent-related bullying/ cyberbullying interventions | 9 | -0.17 | -0.26 | -0.09 |
| Wang (2023) (F) | Parent-related bullying/ cyberbullying interventions | 2 | -0.10 | -0.31 | 0.12 |
| Ng (2020) | Educational interventions | 5 | -0.16 | -0.29 | -0.03 |
| **Cyberbullying victimization** |  |  |  |  |  |
| Chen (2023) | Digital health interventions | 6 | -0.33 | -0.48 | -0.18 |
| Lan (2022) (S) | Anti-cyberbullying educational programs | 16 | -0.21 | -0.30 | -0.12 |
| Lan (2022) (L) | Anti-cyberbullying educational programs | 3 | -0.18 | -0.36 | 0.003 |
| Wang (2023) (E) | Parent-related bullying/ cyberbullying interventions | 10 | -0.17 | -0.24 | -0.10 |
| Wang (2023) (F) | Parent-related bullying/ cyberbullying interventions | 2 | -0.09 | -0.16 | -0.02 |
| Polanin (2022) | Cyberbullying intervention and prevention programs | 39 | -0.13 | -0.21 | -0.05 |
| Kamaruddin (2023) | Cyberbullying intervention and prevention programs | 2 | -0.26 | -0.61 | 0.10 |
| Gaffney (2019) | Cyberbullying intervention and prevention programs | 18 | -0.11 | -0.20 | -0.03 |
| Ng (2020) | Educational interventions | 5 | -0.13 | -0.25 | -0.02 |
| **Total aggression by self-report** | |  |  |  |  |
| Ouyang (2023) | Physical activity interventions | 10 | -0.32 | -0.72 | 0.08 |
| **Indirect aggression** |  |  |  |  |  |
| Ouyang (2023) | Physical activity interventions | 3 | -0.30 | -1.28 | 0.68 |
| **Total aggression by others-report** | |  |  |  |  |
| Ouyang (2023) | Physical activity interventions | 6 | -0.28 | -0.55 | -0.01 |
| **Bullying perpetration** |  |  |  |  |  |
| Chen (2023) | Digital health interventions | 5 | -0.3 | -0.41 | -0.18 |
| Ng (2020) | Educational interventions | 9 | -0.3 | -0.44 | -0.15 |
| Polanin (2022) | Cyberbullying intervention and prevention programs | 22 | -0.18 | -0.28 | -0.08 |
| Gaffney (2021) | School-based bullying/ cyberbullying interventions | NA | -0.15 | -0.12 | -0.18 |
| Merrel (2008) | School-based bullying/ cyberbullying interventions | 8 | 0.04 | NA | NA |
| Hensums (2023) | School-based anti-bullying interventions | NA | -0.07 | -0.13 | -0.01 |
| Fraguas (2021) (E) | School-based anti-bullying interventions | 35 | -0.11 | -0.15 | -0.08 |
| Fraguas (2021) (F) | School-based anti-bullying interventions | 17 | -0.18 | -0.28 | -0.07 |
| Jiménez-Barbero (2016) | School-based anti-bullying interventions | 14 | -0.12 | -0.17 | -0.06 |
| **Cyberbullying bystander** |  |  |  |  |  |
| Chen (2023) | Digital health interventions | 2 | -0.29 | -0.49 | -0.09 |
| Torgal (2023) | School-based bullying/ cyberbullying interventions | 9 | 0.29 | -0.03 | 0.61 |
| **Negative parenting** |  |  |  |  |  |
| Chen (2021) | Parent-related bullying/ cyberbullying interventions* | 3 | -0.20 | -0.33 | -0.06 |
| **Relational bullying victimization** | |  |  |  |  |
| Kennedy (2020) | Bullying prevention programs | 33 | -0.15 | -0.21 | -0.09 |
| **Physical bullying victimization** | |  |  |  |  |
| Kennedy (2020) | Bullying prevention programs | 31 | -0.15 | -0.21 | -0.10 |
| **Cyberbullying** |  |  |  |  |  |
| Chen (2023) | Digital health interventions | 13 | -0.19 | -0.27 | -0.11 |
| Fraguas (2021) | School-based anti-bullying interventions | 5 | -0.14 | -0.20 | -0.07 |
| **Positive interactions with peers** | |  |  |  |  |
| Merrel (2008) | School-based bullying/ cyberbullying interventions | 4 | -0.10 | NA | NA |
| **Aggression propensity** |  |  |  |  |  |
| Ye (2021) | Music-based intervention | 2 | -0.03 | -0.15 | 0.10 |
| **Peer-reported identified victims** | |  |  |  |  |
| Merrel (2008) | School-based bullying/ cyberbullying interventions | 2 | -0.03 | NA | NA |
| **Hyperactivity-impulsivity and aggression** | |  |  |  |  |
| Robinson (1999) | Cognitive behavioral interventions | 23 | 0.74 | -0.25 | 4.80 |
| **Bullying bystander** |  |  |  |  |  |
| Chen (2023) | Digital health interventions | 2 | 0.51 | -0.21 | 1.23 |
| Merrel (2008) | School-based bullying/ cyberbullying interventions | 3 | -0.35 | NA | NA |
| **Staff responses to bullying** | |  |  |  |  |
| Merrel (2008) | School-based bullying/ cyberbullying interventions | 2 | 0.30 | NA | NA |
| **Positive parenting** |  |  |  |  |  |
| Chen (2021) | Parent-related bullying/ cyberbullying interventions | 2 | 0.18 | 0.04 | 0.32 |
| **Intervene to stop bullying** |  |  |  |  |  |
| Merrel (2008) | School-based bullying/ cyberbullying interventions | 3 | 0.17 | NA | NA |
| **Verbal bullying victimization** | |  |  |  |  |
| Kennedy (2020) | Bullying prevention programs | 23 | 0.07 | -0.03 | 0.17 |
| **Reported bullying, or likely to** | |  |  |  |  |
| Merrel (2008) | School-based bullying/ cyberbullying interventions | 4 | 0.07 | NA | NA |
| **Self-reported teacher action/response** | |  |  |  |  |
| Merrel (2008) | School-based bullying/ cyberbullying interventions | 3 | 0.06 | NA | NA |
| **Peer-reported identified aggressors** | |  |  |  |  |
| Merrel (2008) | School-based bullying/ cyberbullying interventions | 3 | 0.04 | NA | NA |
| **Prosocial behavior** |  |  |  |  |  |
| Ye (2021) | Music-based intervention | 3 | 0.03 | -0.03 | 0.09 |
| **Externalizing behavior** |  |  |  |  |  |
| Harwood (2017) | Martial arts | 9 | -0.65 | -1.03 | -0.11 |
| **Self-control** |  |  |  |  |  |
| Ye (2021) | Music-based intervention | 3 | 0.56 | 0.19 | 0.93 |
| **Teacher reported aggression** |  |  |  |  |  |
| Fossum (2008) (ES1) | Psychosocial interventions | 20 | -0.41 | -0.52 | -0.30 |
| Fossum (2008) (ES2) | Psychosocial interventions | 7 | -0.63 | -0.86 | -0.39 |
| **Social functioning** |  |  |  |  |  |
| Fossum (2008) (ES1) | Psychosocial interventions | 13 | -0.42 | -0.57 | -0.27 |
| Fossum (2008) (ES2) | Psychosocial interventions | 10 | -0.49 | -0.73 | -0.24 |
| ***Outcome: Attitude change*** | | | | | |
| Attitudes that discourage bullying | |  |  |  |  |
| Fraguas (2021) (E) | School-based anti-bullying interventions | 25 | 0.20 | 0.15 | 0.25 |
| Fraguas (2021) (F) | School-based anti-bullying interventions | 14 | 0.14 | 0.08 | 0.20 |
| Jiménez-Barbero (2016) | School-based anti-bullying interventions | 4 | 0.06 | 0.03 | 0.10 |
| Merrel (2008) | School-based bullying/ cyberbullying interventions | 4 | 0.06 | NA | NA |
| **Attitudes that encourage bullying** | |  |  |  |  |
| Merrel (2008) | School-based bullying/ cyberbullying interventions | 4 | 0.15 | NA | NA |
| Jiménez-Barbero (2016) | School-based anti-bullying interventions | 3 | -0.18 | -0.30 | -0.06 |
| Fraguas (2021) (E) | School-based anti-bullying interventions | 15 | -0.12 | -0.18 | -0.05 |
| Fraguas (2021) (F) | School-based anti-bullying interventions | 7 | -0.12 | -0.20 | -0.05 |
| **School climate** |  |  |  |  |  |
| Fraguas (2021) (E) | School-based anti-bullying interventions | 12 | 0.07 | 0.04 | 0.10 |
| Fraguas (2021) (F) | School-based anti-bullying interventions | 5 | 0.14 | 0.04 | 0.23 |
| Jiménez-Barbero (2016) | School-based anti-bullying interventions | 3 | -0.03 | -0.08 | 0.02 |
| **Parent-child communication about bullying** | |  |  |  |  |
| Merrel (2008) | School-based bullying/ cyberbullying interventions | 2 | -0.04 | NA | NA |
| ***Outcome: Mental health*** | | | | | |
| **Anger** |  |  |  |  |  |
| Ouyang (2023) | Physical activity interventions | 6 | -0.49 | -1.16 | 0.17 |
| Yang (2023) | Sports intervention | 5 | -0.26 | -0.56 | 0.03 |
| **Depression or depression symptom** | |  |  |  |  |
| Chen (2021) | Parent-related bullying/ cyberbullying interventions * | 2 | 0.38 | -0.06 | 0.82 |
| Guzman-Holst (2022) | School-based anti-bullying interventions | 12 | -0.06 | -0.01 | -0.11 |
| **Hostility** |  |  |  |  |  |
| Ouyang (2023) | Physical activity interventions | 6 | -0.33 | -0.69 | 0.04 |
| Yang (2023) | Sports intervention | 5 | -0.29 | -0.41 | -0.17 |
| **Mental health problems** |  |  |  |  |  |
| Fraguas (2021) (E) | School-based anti-bullying interventions | 20 | -0.21 | -0.28 | -0.13 |
| Fraguas (2021) (F) | School-based anti-bullying interventions | 6 | -0.20 | -0.35 | -0.06 |
| **Anxiety** |  |  |  |  |  |
| Guzman-Holst (2022) | School-based anti-bullying interventions | 14 | -0.08 | -0.16 | -0.01 |
| **Internalizing symptoms** |  |  |  |  |  |
| Guzman-Holst (2022) | School-based anti-bullying interventions | 22 | -0.06 | -0.10 | -0.03 |
| **Hyperactivity-impulsivity** |  |  |  |  |  |
| Ye (2021) | Music-based intervention | 3 | -0.02 | -0.70 | 0.66 |
| Robinson (1999) | Cognitive behavioral interventions | 17 | 0.79 | -0.24 | 4.80 |
| **Empathy** |  |  |  |  |  |
| Merrel (2008) | School-based bullying/ cyberbullying interventions | 2 | -0.10 | NA | NA |
| **Parental distress** |  |  |  |  |  |
| Fossum (2008) (ES1) | Psychosocial interventions | 16 | -0.39 | -0.57 | -0.23 |
| Fossum (2008) (ES2) | Psychosocial interventions | 17 | -0.47 | -0.59 | -0.36 |

**Notes:** E: End of intervention; F: Follow-up; S: Short term; L: Long term; ES1: Studies involving an untreated control; ES2: Studies involving either a treated control or no control; n: Specific number of articles of each outcome; 95% CI: 95% Confidence Interval.

* The original values were -0.381 [-0.060, 0.821] and -0.195 [0.061 to 0.329] and were adjusted to 0.381 [-0.060, 0.821] and -0.195 [-0.329 to -0.061] throughout the study, respectively, which was checked with the first author via email.

To ensure consistency, the effect sizes of the following studies were multiplied by -1, where the prevention intervention was verified as preventive and beneficial, and the original effect size values were reported as greater than 0 (54, 55): Chen (2021) (20), Guzman- Holst (2022) (61), Kennedy (2020) (62), Castillo-Eito (2020) (28), Harwood (2017) (14), Chen (2023) (63), Gaffney (2021) (40), Gaffney (2019) (64), Fossum (2008) (27), and Tao (2021) (65).

# Table S15b. Cohen's d by outcome subgroup (Simplified version, consistent with forest plot)

| **First author (year)** | **Intervention** | **n** | **Cohen's d** | **95% CI** | |
| --- | --- | --- | --- | --- | --- |
|  |  |  |  | **Lower** | **Higher** |
| ***Outcome: Behavior change*** | | | | | |
| **Aggressive behaviors** |  |  |  |  |  |
| Ye (2021) | Music-based intervention | 4 | -0.99 | -1.42 | -0.56 |
| Tao (2021) | Mindfulness-based interventions | 10 | -0.48 | -0.63 | -0.33 |
| Yang (2023) | Sports intervention | 9 | -0.37 | -0.69 | -0.06 |
| Shen (2024) | School-based anti-aggression programs | 17 | -0.35 | -0.53 | -0.18 |
| Castillo-Eito (2020) | Psychosocial interventions | 95 | -0.28 | -0.39 | -0.17 |
| Fossum (2008) | Psychosocial interventions | 33 | -0.62 | -0.76 | -0.49 |
| Jiang (2024) | Community-based programs | 16 | -0.26 | -0.39 | -0.13 |
| Smeets (2015) | Cognitive behavioral interventions | 25 | 0.50 | -0.19 | 2.35 |
| Barnes (2014) | Cognitive behavioral interventions | 25 | -0.14 | -3.23 | 0.62 |
| Hoogsteder (2015) | Cognitive behavioral interventions | 6 | 1.139 | -0.06 | 3.13 |
| Robinson (1999) | Cognitive behavioral interventions | 12 | 0.64 | -0.25 | 1.67 |
| **Overall bullying** |  |  |  |  |  |
| Chen (2021) | Parent-related bullying/ cyberbullying interventions | 16 | -0.64 | -1.04 | -0.24 |
| Chen (2023) | Digital health interventions | 16 | -0.28 | -0.42 | -0.14 |
| Fraguas (2021) | School-based anti-bullying interventions | 45 | -0.15 | -0.19 | -0.11 |
| **Combined bullying** | |  |  |  |  |
| Chen (2023) | Digital health interventions | 2 | -0.40 | -0.52 | -0.28 |
| **Bullying bully–victim dual roles** | |  |  |  |  |
| Chen (2023) | Digital health interventions | 2 | -0.63 | -1.21 | -0.05 |
| **Verbal aggression** |  |  |  |  |  |
| Ouyang (2023) | Physical activity interventions | 5 | -0.63 | -2.04 | 0.78 |
| **Bullying victimization** |  |  |  |  |  |
| Chen (2023) | Digital health interventions | 5 | -0.37 | -0.51 | -0.24 |
| Ng (2020) | Educational interventions | 9 | -0.18 | -0.26 | -0.10 |
| Polanin (2022) | Cyberbullying intervention and prevention programs | 24 | -0.16 | -0.27 | -0.05 |
| Hensums (2023) | School-based anti-bullying interventions | NA | -0.14 | -0.19 | -0.10 |
| Fraguas (2021) | School-based anti-bullying interventions | 32 | -0.16 | -0.23 | -0.09 |
| Jiménez-Barbero (2016) | School-based anti-bullying interventions | 8 | -0.09 | -0.18 | 0.01 |
| Lee (2015) | School-based anti-bullying interventions | 13 | -0.15 | -0.20 | -0.10 |
| Gaffney (2021) | School-based bullying/ cyberbullying interventions | NA | -0.12 | -0.10 | -0.15 |
| Merrel (2009) | School-based bullying/ cyberbullying interventions | 10 | 0.27 | NA | NA |
| **Physical aggression** |  |  |  |  |  |
| Ouyang (2023) | Physical activity interventions | 6 | -0.45 | -1.09 | 0.20 |
| **Bullying** |  |  |  |  |  |
| Chen (2023) | Digital health interventions | 10 | -0.41 | -0.68 | -0.14 |
| **Cyberbullying perpetration** |  |  |  |  |  |
| Chen (2023) | Digital health interventions | 4 | -0.35 | -0.58 | -0.12 |
| Lan (2022) | Anti-cyberbullying educational programs | 19 | -0.22 | -0.30 | -0.14 |
| Polanin (2022) | Cyberbullying intervention and prevention programs | 44 | -0.18 | -0.28 | -0.09 |
| Kamaruddin (2023) | Cyberbullying intervention and prevention programs | 4 | -0.15 | -0.31 | 0.01 |
| Gaffney (2019) | Cyberbullying intervention and prevention programs | 18 | -0.12 | -0.21 | -0.02 |
| Wang (2023) | Parent-related bullying/ cyberbullying interventions | 9 | -0.17 | -0.26 | -0.09 |
| Ng (2020) | Educational interventions | 5 | -0.16 | -0.29 | -0.03 |
| **Cyberbullying victimization** |  |  |  |  |  |
| Chen (2023) | Digital health interventions | 6 | -0.33 | -0.48 | -0.18 |
| Lan (2022) | Anti-cyberbullying educational programs | 16 | -0.21 | -0.30 | -0.12 |
| Wang (2023) | Parent-related bullying/ cyberbullying interventions | 10 | -0.17 | -0.24 | -0.10 |
| Polanin (2022) | Cyberbullying intervention and prevention programs | 39 | -0.13 | -0.21 | -0.05 |
| Kamaruddin (2023) | Cyberbullying intervention and prevention programs | 2 | -0.26 | -0.61 | 0.10 |
| Gaffney (2019) | Cyberbullying intervention and prevention programs | 18 | -0.11 | -0.20 | -0.03 |
| Ng (2020) | Educational interventions | 5 | -0.13 | -0.25 | -0.02 |
| **Total aggression by self-report** | |  |  |  |  |
| Ouyang (2023) | Physical activity interventions | 10 | -0.32 | -0.72 | 0.08 |
| **Indirect aggression** |  |  |  |  |  |
| Ouyang (2023) | Physical activity interventions | 3 | -0.30 | -1.28 | 0.68 |
| **Total aggression by others-report** | |  |  |  |  |
| Ouyang (2023) | Physical activity interventions | 6 | -0.28 | -0.55 | -0.01 |
| **Bullying perpetration** |  |  |  |  |  |
| Chen (2023) | Digital health interventions | 5 | -0.30 | -0.41 | -0.18 |
| Ng (2020) | Educational interventions | 9 | -0.30 | -0.44 | -0.15 |
| Polanin (2022) | Cyberbullying intervention and prevention programs | 22 | -0.18 | -0.28 | -0.08 |
| Gaffney (2021) | School-based bullying/ cyberbullying interventions | NA | -0.15 | -0.12 | -0.18 |
| Merrel (2008) | School-based bullying/ cyberbullying interventions | 8 | 0.04 | NA | NA |
| Hensums (2023) | School-based anti-bullying interventions | NA | -0.07 | -0.13 | -0.01 |
| Fraguas (2021) | School-based anti-bullying interventions | 35 | -0.11 | -0.15 | -0.08 |
| Jiménez-Barbero (2016) | School-based anti-bullying interventions | 14 | -0.12 | -0.17 | -0.06 |
| **Cyberbullying bystander** |  |  |  |  |  |
| Chen (2023) | Digital health interventions | 2 | -0.29 | -0.49 | -0.09 |
| Torgal (2023) | School-based bullying/ cyberbullying interventions | 9 | 0.29 | -0.03 | 0.61 |
| **Negative parenting** |  |  |  |  |  |
| Chen (2021) | Parent-related bullying/ cyberbullying interventions * | 3 | -0.20 | -0.33 | -0.06 |
| **Relational bullying victimization** | |  |  |  |  |
| Kennedy (2020) | Bullying prevention programs | 33 | -0.15 | -0.21 | -0.09 |
| **Physical bullying victimization** | |  |  |  |  |
| Kennedy (2020) | Bullying prevention programs | 31 | -0.15 | -0.21 | -0.10 |
| **Cyberbullying** |  |  |  |  |  |
| Chen (2023) | Digital health interventions | 13 | -0.19 | -0.27 | -0.11 |
| Fraguas (2021) | School-based anti-bullying interventions | 5 | -0.14 | -0.20 | -0.07 |
| **Positive interactions with peers** | |  |  |  |  |
| Merrel (2008) | School-based bullying/ cyberbullying interventions | 4 | -0.10 | NA | NA |
| **Aggression propensity** |  |  |  |  |  |
| Ye (2021) | Music-based intervention | 2 | -0.03 | -0.15 | 0.10 |
| **Peer-reported identified victims** | |  |  |  |  |
| Merrel (2008) | School-based bullying/ cyberbullying interventions | 2 | -0.03 | NA | NA |
| **Hyperactivity-impulsivity and aggression** | |  |  |  |  |
| Robinson (1999) | Cognitive behavioral interventions | 23 | 0.74 | -0.25 | 4.80 |
| **Bullying bystander** |  |  |  |  |  |
| Chen (2023) | Digital health interventions | 2 | 0.51 | -0.21 | 1.23 |
| Merrel (2008) | School-based bullying/ cyberbullying interventions | 3 | -0.35 | NA | NA |
| **Staff responses to bullying** | |  |  |  |  |
| Merrel (2008) | School-based bullying/ cyberbullying interventions | 2 | 0.30 | NA | NA |
| **Positive parenting** |  |  |  |  |  |
| Chen (2021) | Parent-related bullying/ cyberbullying interventions | 2 | 0.18 | 0.04 | 0.32 |
| **Intervene to stop bullying** |  |  |  |  |  |
| Merrel (2008) | School-based bullying/ cyberbullying interventions | 3 | 0.17 | NA | NA |
| **Verbal bullying victimization** | |  |  |  |  |
| Kennedy (2020) | Bullying prevention programs | 23 | 0.07 | -0.03 | 0.17 |
| **Reported bullying, or likely to** | |  |  |  |  |
| Merrel (2008) | School-based bullying/ cyberbullying interventions | 4 | 0.07 | NA | NA |
| **Self-reported teacher action/response** | |  |  |  |  |
| Merrel (2008) | School-based bullying/ cyberbullying interventions | 3 | 0.06 | NA | NA |
| **Peer-reported identified aggressors** | |  |  |  |  |
| Merrel (2008) | School-based bullying/ cyberbullying interventions | 3 | 0.04 | NA | NA |
| **Prosocial behavior** |  |  |  |  |  |
| Ye (2021) | Music-based intervention | 3 | 0.03 | -0.03 | 0.09 |
| **Externalizing behavior** |  |  |  |  |  |
| Harwood (2017) | Martial arts | 9 | -0.65 | -1.03 | -0.11 |
| **Self-control** |  |  |  |  |  |
| Ye (2021) | Music-based intervention | 3 | 0.56 | 0.19 | 0.93 |
| **Teacher reported aggression** |  |  |  |  |  |
| Fossum (2008) | Psychosocial interventions | 20 | -0.41 | -0.52 | -0.30 |
| **Social functioning** |  |  |  |  |  |
| Fossum (2008) | Psychosocial interventions | 13 | -0.42 | -0.57 | -0.27 |
| ***Outcome: Attitude change*** | | | | | |
| Attitudes that discourage bullying | |  |  |  |  |
| Fraguas (2021) | School-based anti-bullying interventions | 25 | 0.20 | 0.15 | 0.25 |
| Jiménez-Barbero (2016) | School-based anti-bullying interventions | 4 | 0.06 | 0.03 | 0.10 |
| Merrel (2008) | School-based bullying/ cyberbullying interventions | 4 | 0.06 | NA | NA |
| **Attitudes that encourage bullying** | |  |  |  |  |
| Merrel (2008) | School-based bullying/ cyberbullying interventions | 4 | 0.15 | NA | NA |
| Jiménez-Barbero (2016) | School-based anti-bullying interventions | 3 | -0.18 | -0.30 | -0.06 |
| Fraguas (2021) | School-based anti-bullying interventions | 15 | -0.12 | -0.18 | -0.05 |
| **School climate** |  |  |  |  |  |
| Fraguas (2021) | School-based anti-bullying interventions | 12 | 0.07 | 0.04 | 0.10 |
| Jiménez-Barbero (2016) | School-based anti-bullying interventions | 3 | -0.03 | -0.08 | 0.02 |
| **Parent-child communication about bullying** | |  |  |  |  |
| Merrel (2008) | School-based bullying/ cyberbullying interventions | 2 | -0.04 | NA | NA |
| ***Outcome: Mental health*** | | | | | |
| **Anger** |  |  |  |  |  |
| Ouyang (2023) | Physical activity interventions | 6 | -0.49 | -1.16 | 0.17 |
| Yang (2023) | Sports intervention | 5 | -0.26 | -0.56 | 0.03 |
| **Depression or depression symptom** | |  |  |  |  |
| Chen (2021) | Parent-related bullying/ cyberbullying interventions * | 2 | 0.38 | -0.06 | 0.82 |
| Guzman-Holst (2022) | School-based anti-bullying interventions | 12 | -0.06 | -0.01 | -0.11 |
| **Hostility** |  |  |  |  |  |
| Ouyang (2023) | Physical activity interventions | 6 | -0.33 | -0.69 | 0.04 |
| Yang (2023) | Sports intervention | 5 | -0.29 | -0.41 | -0.17 |
| **Mental health problems** |  |  |  |  |  |
| Fraguas (2021) | School-based anti-bullying interventions | 20 | -0.21 | -0.28 | -0.13 |
| **Anxiety** |  |  |  |  |  |
| Guzman-Holst (2022) | School-based anti-bullying interventions | 14 | -0.08 | -0.16 | -0.01 |
| **Internalizing symptoms** |  |  |  |  |  |
| Guzman-Holst (2022) | School-based anti-bullying interventions | 22 | -0.06 | -0.10 | -0.03 |
| **Hyperactivity-impulsivity** |  |  |  |  |  |
| Ye (2021) | Music-based intervention | 3 | -0.02 | -0.70 | 0.66 |
| Robinson (1999) | Cognitive behavioral interventions | 17 | 0.79 | -0.24 | 4.80 |
| **Empathy** |  |  |  |  |  |
| Merrel (2008) | School-based bullying/ cyberbullying interventions | 2 | -0.10 | NA | NA |
| **Parental distress** |  |  |  |  |  |
| Fossum (2008) | Psychosocial interventions | 16 | -0.39 | -0.57 | -0.23 |

**Notes:** n: Specific number of articles of each outcome; 95% CI: 95% Confidence Interval.

* The original values were -0.381 [-0.060, 0.821] and -0.195 [0.061 to 0.329] and were adjusted to 0.381 [-0.060, 0.821] and -0.195 [-0.329 to -0.061] throughout the study, respectively, which was checked with the first author via email.

To ensure consistency, the effect sizes of the following studies were multiplied by -1, where the prevention intervention was verified as preventive and beneficial, and the original effect size values were reported as greater than 0 (54, 55): Chen (2021) (20), Guzman- Holst (2022) (61), Kennedy (2020) (62), Castillo-Eito (2020) (28), Harwood (2017) (14), Chen (2023) (63), Gaffney (2021) (40), Gaffney (2019) (64), Fossum (2008) (27), and Tao (2021) (65).
